# Tracing socioeconomic change and emerging elites in 4^th^–3^rd^ century BCE: Metalwork from the Amber Road Corridor

**DOI:** 10.1371/journal.pone.0352885

**Published:** 2026-09-02

**Authors:** Alžběta Danielisová, Daniel Bursák, Samuel Kertés, Ivan Čižmář, Hana Čižmářová, Jana Čižmářová, Tomáš Mangel, Ladislav Strnad, Jakub Trubač

**Affiliations:** 1 Institute of Archaeology of the Czech Academy of Sciences, Prague, Czech Republic; 2 Ústav Archeologické Památkové Péče Brno, Brno, Czech Republic; 3 Moravian Museum, Brno, Czech Republic; 4 University Hradec Kralove, Hradec Králové, Czech Republic; 5 Faculty of Sciences, Charles University, Prague, Czech Republic; Universidad de Sevilla, SPAIN

## Abstract

Leaded bronze ornaments of the La Tène period provide a key lens on how technological innovation intersected with social and economic change in Iron Age Central Europe. In the 4^th^–3^rd^ centuries BCE, the Middle Danube formed a major communication corridor between the Balkans and the Transalpine zone. Copper-alloy personal ornaments became central to changing expressions of identity and status, while also reflecting shifts in alloy recipes, raw-material procurement, and craft organisation. A major 3^rd^-century BCE development was the widespread adoption of leaded bronze, well suited to casting high-relief “Plastic-Style” ornamentation. It remains debated whether this innovation was introduced primarily through emergent agglomeration sites or developed within established local communities embedded in regional networks. We address this question through lead isotope and trace-element analyses of copper-alloy ornaments from 4^th^–3^rd^ century BCE cemeteries in Bohemia and Moravia, compared with material from the open industrial agglomeration of Němčice nad Hanou. Lead isotope ratios are used to assess metal provenance, while trace-element compositions characterise alloying and casting practices. These data are integrated with chronological, typological, spatial, and decorative evidence, using KDE-based matching, conventional isotopic plots, and multivariate trace-element analysis. The results show a diachronic shift from heterogeneous 4^th^-century BCE alloying to more standardised leaded bronze recipes in the 3^rd^ century BCE. This technological regime combined improved castability, reduced tin demand through lead addition, and the visual demands of Plastic-Style ornamentation, producing a prestige-coded but widely circulating “bulk luxury” commodity. Lead isotope signatures indicate increasingly structured supply chains, including recurrent use of lead sources consistent with central and western German deposits. Overlaps between cemetery assemblages and Němčice suggest that agglomerations scaled and integrated innovations already present in community-based production rather than initiating them. We argue that rural cemetery communities were active arenas of metallurgical innovation, and that 3^rd^-century BCE agglomerations functioned as accelerators of locally rooted technological and social trends.

## Introduction

As a key geographical hub in Iron Age Central Europe, the Middle Danube region exemplifies how certain areas played a pivotal role in facilitating cross-regional social and economic networks. During the 4^th^ century BCE, material culture—shaped by influences from the Middle Rhine, the Swiss Plateau, and the Bohemian lowlands [1]—disseminated across territories associated with the La Tène cultural complex [2]. By the 3^rd^ century BCE, the direction of influence had shifted with influences believed to originate in the southeastern La Tène zone, in contact with Thracian and Hellenistic civilisations, spread westward [3,4], introducing new decorative motifs, technological practices, and economic strategies. Throughout this process, the Danube functioned as a critical communication corridor, enabling long-distance cultural transmission between the Balkans and the Transalpine zone.

The societal transformations of the 3^rd^ century BCE deeply affected community organisation, ritual expression, and material culture. Archaeological evidence from La Tène period sites in this region reflects a marked reconfiguration of settlement patterns, technological practices, and social structures [1, 5]. Of particular interest are the rise of material-rich, open agglomerations with concentrations of specialised production, which have become central to discussions surrounding Iron Age complexity, centralisation, and the emergence of early industrial economies [6]. Notable examples include Roseldorf and Haselbach in Lower Austria [7,8], Němčice nad Hanou in central Moravia [9,10], and Nowa Cerekwia in Upper Silesia [11]. These agglomerations coexisted, at least for a time, with rural flat cemeteries established in the beginning of the 4^th^ century BCE, which represent a continuation of earlier settlement patterns and a trajectory of steady social and economic development. These cemeteries offer a unique window into community-level practices and the dynamics of technological and social change that ultimately gave rise to these large-scale settlements.

These dynamics are particularly evident in the evolving use of copper alloys. Personal ornaments such as jewellery, reflecting shifting stylistic trends, not only served as markers of identity and status but also acted as vehicles for technological change. A major innovation of the 3^rd^ century BCE was the increasing use of **leaded bronze**, a ternary alloy of copper, tin, and lead [5]. While copper-tin alloys had long been established in European metallurgical traditions, the systematic incorporation of lead represented a significant shift in both technological practice and material preference. Leaded bronze allowed greater fluidity in casting and was particularly suited to producing high-relief, plastic forms for decorative elements of personal attires [12–14], such as bracelets, anklets, torcs, belt sets etc., found largely in La Tène burial contexts. The distinctive visual and technical quality of this metalwork found its most expressive manifestation in what is known as the ‘**Plastic-Style**’—an artistic expression that became the most widespread style of La Tène art across Europe, synthesising indigenous and Hellenistic influences into dynamic, voluminous forms [15–18]. This technological and stylistic transition coincides with broader cultural and economic changes traditionally attributed to intensified interaction with Thracian and Hellenistic production centres. Increased mobility between southeastern and central Europe, particularly through the Carpathian Basin, has been proposed as a driver of both metallurgical innovation and stylistic convergence [3,19]. However, the extent to which these developments represented the diffusion of external techniques, rather than local adaptation or invention, remains underexplored. Recent scholarship has prioritised agglomeration sites as the engines of socio-economic transformation, yet the present study proposes a revised perspective: that key technological innovations and their social consequences were already emerging within communities in rural settlements, visible in funerary contexts prior to their consolidation in centralised production hubs.

Leaded bronze thus serves as a critical material lens through which to investigate broader processes of socio-economic transformations in the La Tène world. This study addresses whether the adoption of leaded bronze in Bohemian and Moravian cemeteries reflects a diffusion of innovation from southeastern Europe or a regionally distinct trajectory of technological change. We examine to what extent alloying practices were influenced by long-distance trade, elite-controlled workshops, or community-based knowledge systems, and how these intersected with emerging social patterns in access to resources and production technologies.

**Fig 1:**
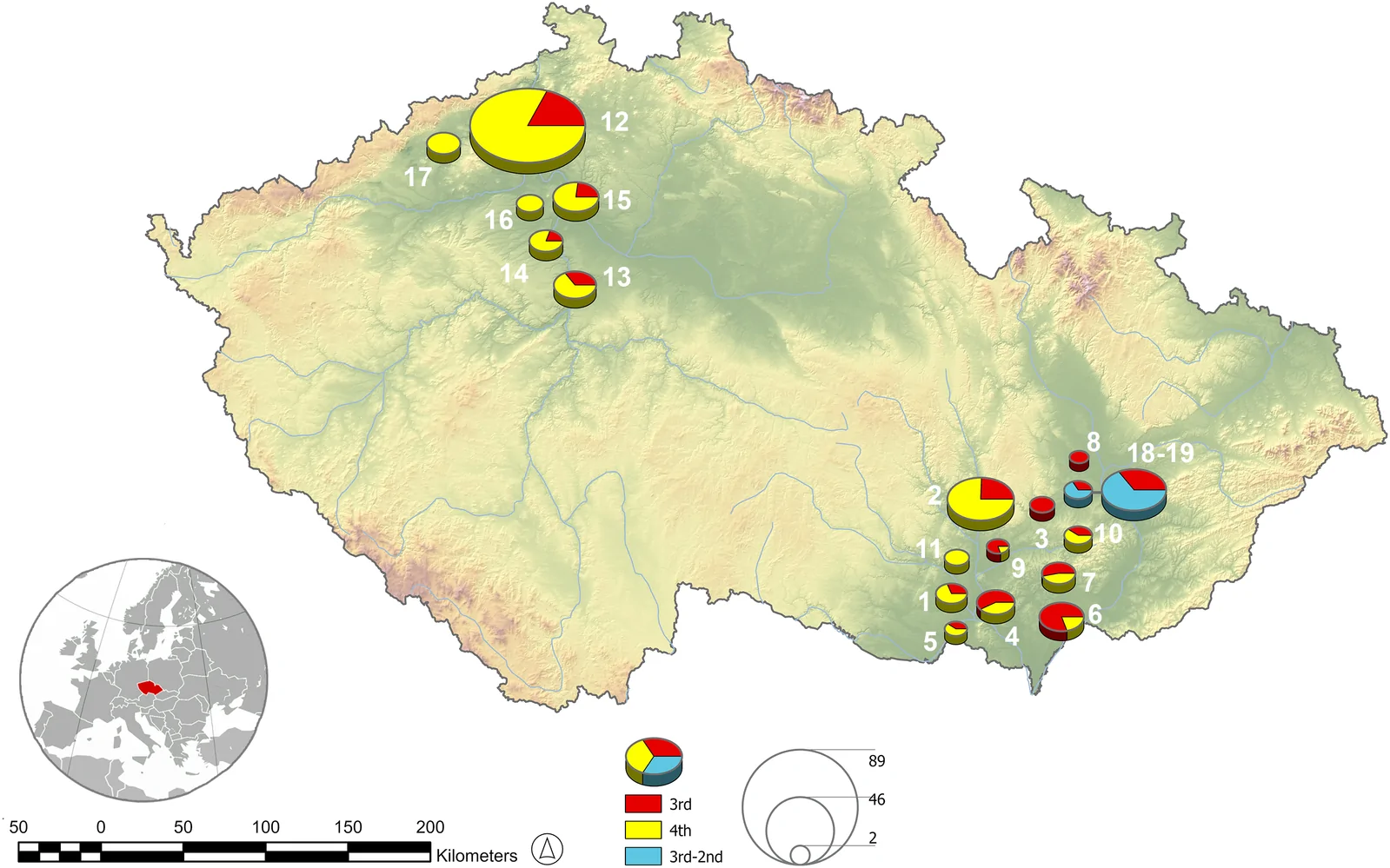
Study area with sites with analysed bronze assemblages. The numbering of sites refers to numbers in Table 1. Legend: colours in pie charts are categories according to dating of the objects. Pie chart sizes are normalised by the total number of samples per site (cf. Table 1), (source maps: the Europe inset downloaded from wikimedia commons, CC BY-SA 4.0; map – created in ArcGIS Pro, background: DMR 5G Map by ČUZK, 2026, www.cuzk.gov.cz, CC-BY 4.0; graphics by A.D.).

To investigate these questions, we apply a combination of **lead isotopic provenance** and **element concentration analyses** [1,5,20,21] to a substantial assemblage of copper-alloy artefacts, primarily drawn from 4^th^–3^rd^ century BCE funerary contexts. The sites under study, situated in the Bohemian and Moravian lowlands, are compared with material from the agglomeration of Němčice nad Hanou (Fig 1), which serves as a reference point for an idea of centralised production. Lead isotope ratios are used to trace the geological origin of the copper-alloy components, particularly copper and lead, while trace element analysis additionally provides data also on alloy composition and casting practices. Particular attention is given to diachronic trends, internal variability in alloy types, and technological and raw material overlap between funerary and agglomeration contexts. By foregrounding cemeteries as active arenas of technological innovation and social change, this study re-evaluates their role in processes of economic transformation typically associated with agglomeration sites. Ultimately, the goal of this research is to understand how the spread of technological innovations— particularly the adoption of leaded bronze —contributed to the rise of socio-political complexity in Iron Age Central Europe.

## Materials and methods

### Materials

#### Sample selection.

**Fig 2:**
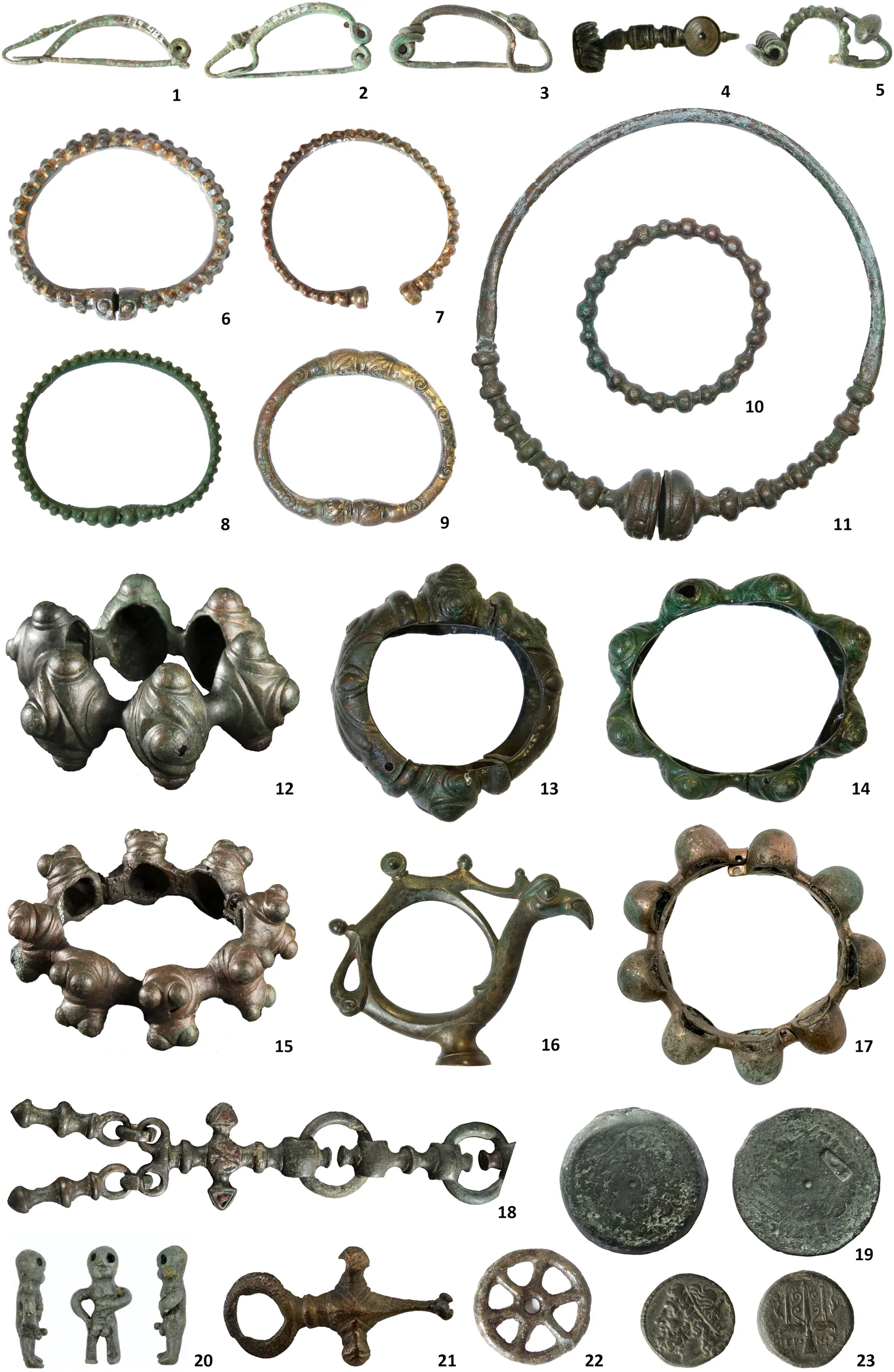
La Tène copper alloys from the 4^th^ - 2^nd^ century BCE from the study area. 1-11 - 4^th^ century BCE, 12-18 - 3^rd^ century BCE (12-15 Plastic-Style jewellery), 20-22 – agglomeration of Němčice, 19, 23 – Mediterranean coins from the agglomeration (photo: D. Bursák, graphics: A. Danielisová).

This case study focuses on personal, decorative, and other small copper-alloy objects from La Tène cemeteries from the Czech Republic, dated to the 4^th^ and 3^rd^ centuries BCE (Fig 2). The selection of 399 samples (S1 Table) was based on the chronological determination, social context, and relevance to investigated technological processes. The assemblage included 153 samples from Moravian cemeteries, 177 samples from similar sites in Bohemia, and 69 samples from the agglomeration of Němčice nad Hanou (Table 1) which included also 30 samples of imported copper-alloy coins from the Mediterranean area, from the collections of the Moravian Museum in Brno, Czech Republic. No permits were required for the described study, which complied with all relevant regulations. All samples were archaeologically contextualised in terms of their location, typology, and chronological classification.

**Table 1:** Sample distribution by century (4^th^, 3^rd^, 3^rd^–2^nd^ BCE), region (Bohemia/Moravia), and site type. The ID numbers refer to numbers in Fig 1.

ID	SITE	region	type	Number of objects (by century BCE)				long	lat
				4^th^	3^rd^	3^rd^-2^nd^	total		
1	**Blučina**	Moravia	cemetery	3	2	0	5	16.65928	49.05354
2	**Brno – Maloměřice**	Moravia	cemetery	34	11	0	45	16.64574	49.21459
3	**Holubice**	Moravia	cemetery	9	4	0	13	16.80751	49.1764
4	**Hustopeče**	Moravia	cemetery	8	11	0	19	16.72802	48.94865
5	**Lovčičky**	Moravia	cemetery	6	4	0	10	16.85383	49.07043
6	**Mistřín**	Moravia	cemetery	5	20	0	25	17.08731	48.97349
7	**Nechvalín**	Moravia	cemetery	7	8	0	15	17.07102	49.05291
8	**Němčice nad Hanou**	Moravia	cemetery	0	2	0	2	17.18273	49.3563
9	**Ponětovice**	Moravia	cemetery	1	4	0	5	16.73965	49.14938
10	**Pustiměřské Prusy**	Moravia	cemetery	0	7	0	7	17.0184	49.29812
11	**Tikovice**	Moravia	cemetery	7	0	0	7	16.51578	49.10923
12	**Prosmyky**	Bohemia	cemetery	73	16	0	89	14.08557	50.51255
13	**Prague**	Bohemia	cemetery	15	8	0	23	14.43341	50.07619
14	**Mlčechvosty**	Bohemia	cemetery	9	0	0	9	14.32287	50.31947
15	**Vliněves**	Bohemia	cemetery	20	6	0	26	14.43826	50.3711
16	**Zeměchy**	Bohemia	cemetery	12	3	0	15	14.27559	50.23051
17	**Jenišův Újezd**	Bohemia	cemetery	15	0	0	15	13.71697	50.57089
18	**Němčice nad Hanou**	Moravia	agglomeration	0	15	28	43	17.18866	49.35107
19	**Němčice nad Hanou-coins**	Moravia	agglomeration	0	20	40	10	17.18866	49.35107

Dating of samples within the Central European La Tène period relative chronology [22] was used as follows:

- **4**^**th**^ **century BCE** (410 - 300/290):
  - LT B1a (410–390 BCE)
  - LT B1 (400–350 BCE)
  - LT B2a (350–300 BCE)
- **3**^**rd**^ **century BCE** (300/290–200/180 BCE):
  - LT B2b (300–260 BCE)
  - LTB2b-C1 (260–230 BCE)
  - LT C1 (230–200/180 BCE)
- **2**^**nd**^ **century BCE** (200/180–120 BCE)
  - LT C2 (200/180–120 BCE)

### Methods

To ensure the integrity of the samples, material was extracted from the metal core, avoiding corrosion layers [23]. Between 30 and 50 mg of alloy was collected for analysis, following standard ICP-MS protocols, including dissolution in HNO3 and Pb separation. Analytical methods included NEPTUNE Plus MC-ICP-MS for lead isotope analysis and ICP-MS for chemical composition studies, to investigate the artefacts’ composition and potential provenance. A smaller subset of samples was tested using both ICP-MS and NAA.

The isotopic and element concentration analyses were performed using Neptune MC-ICP-MS (Thermo-Fisher Scientific). The external precision for the MC-ICP-MS measurements of ^206^Pb/^204^Pb and ^207^Pb/^204^Pb ratios are below 0.006 (2SD) and of ^208^Pb/^204^Pb below 0.01 (2SD), respectively, as determined from long-term reproducibility of SRM NIST 981.

### Data evaluation

#### Data structure and patterning.

First, the datasets were analysed for elemental correlations using standard correlation matrices and ternary graphs. This analysis highlighted affinities to specific elements or element combinations associated with distinct types of copper ores: Sb+As+Ag (tetrahedrite-fahlore type ores), Zn (polymetallic galena ores), and Co + Ni (chalcopyrite ores).

To detect internal data structure, trace element chemistry was used to allocate data to sub-groups by applying multivariate statistical methods (S1 Text). After basic correlation analysis, a **principal component analysis (PCA)** was applied to trace element compositions to detect group-specific combinations of minor and trace elements following the method used in Danielisová et al. [1]. As previously tested, only provenance-indicative elements (Co, Ni, Zn, As, Ag, Sb, Pb and Bi) were included in the analysis. Lead was also excluded, as it represented a deliberate alloying addition rather than a mineral-derived component. All variables were log-transformed prior to analysis to reduce the influence of scale differences as values often spanned up to an order of magnitude. The PCA was run twice: first on the selected trace elements together with Pb isotopes, and then on the trace elements alone (S1 Text). **K-means clustering analysis** [24] was subsequently applied to the factor scores derived from the PCA to evaluate potential groupings.

Datasets were subjected to PCA in various combinations to identify chronological, geographical, or other possible trends. The analysis first included all samples from cemeteries in the dataset, followed by PCA runs focused on geographical versus chronological trends. Following that, it was refined to focus on 3^rd^ century BCE Moravian cemeteries and the agglomeration of Němčice, limiting the dataset to samples considered the most likely recipients of non-local objects or technological trends. Resulting patterns of alignment or differentiation in the use of raw materials, specifically copper and lead, were expected to provide insights into the extent of technological networks or the degree of potential individualistic production between communities.

#### Provenance analysis.

The lead isotope database of ore fields (LIDOF), comprising 12 741 individually analysed ore samples (S2 Table), serves as a comparative dataset for **lead isotopes-based provenance analysis** and is detailed in S2 Text. This database was compiled from published literature and publicly available sources, including OXALID [25] and TerraLID [26,27]. All samples include data for the three fundamental isotope ratios: ^206^Pb/^204^Pb, ^207^Pb/^204^Pb, and ^208^Pb/^204^Pb. LIDOF covers Europe, North Africa, and the Middle East, aligning with the geographical scope of this study. The ores were hierarchically categorised into three levels. The first level is based on the association with specific orogenies. The second categorisation involves affiliation with particular ore horizons or mountain ranges. The third level provides the most detailed geographical differentiation, reflecting the level of detail found in the original publications or databases.

The provenance of the samples was in the first step assessed using lead model age analysis, which integrates chronometric and geochemical data from the geological background to reflect the tectonic ages of the lead within the samples [28]. This method enables a basic distribution analysis of sample origins without needing comparative Pb isotopic datasets of particular deposits. This way, the potential absence of comparable data is compensated for by the geological determination of samples. For this purpose, the Pb Model Age “T”, the “mu” (μ) constant, and kappa coefficient (“Th/U (κ)”) for each sample were calculated using Stacey and Kramer’s two-stage Pb evolution algorithm [29], provided by the Lead Isotope Database (GlobaLID) [27].

The following provenance analysis was based on several consecutive steps. The first step involves categorising the data according to the Model Age calculations [29]. Due to the large volume of data, a non-parametric statistical evaluation was performed using kernel density estimations (KDE) and overlap score evaluations as the main analytical tool [30,31]. For a more detailed inspection, we conducted a comparison of the analysed assemblages, categorised by century, with individual geological units. This was done using traditional biplots, which compare data distribution and basic data trend lines in a 2D space. These comparisons are available in S2 Text.

All statistical evaluations were conducted in the RStudio environment and Python 3 (https://cran.rstudio.com, www.python.org). The individual codes are available in Supplementary Information 5 (S1 Code). The estimation of the most probable deposit of origin was carried out using a model pipeline consisting of several steps. Kernel density estimation (KDE) parameters were defined as follows: the estimation was performed on three isotopic ratios—^206^Pb/^204^Pb, ^207^Pb/^204^Pb, and ^208^Pb/^204^Pb. For each artefact, the KDE was integrated within a defined range around its isotopic values (±0.01 for ^206^Pb/^204^Pb and ^207^Pb/^204^Pb, ±0.03 for ^208^Pb/^204^Pb) [31]. This integration yielded probability scores that quantify the alignment between the isotopic composition of each artefact and the reference dataset [32]. Regions were then filtered using a predefined probability threshold of 10% for all three isotopic ratios [30]. This step served to exclude regions with weak or inconsistent matches. For regions exceeding the threshold, the geometric means of the three probabilities were calculated to highlight isotopic coherence. The geometric mean was preferred over the arithmetic mean due to its robustness in capturing consistency across isotopic ratios [33]. The region with the highest geometric mean probability was identified as the most likely source of the sample.

To facilitate a more meaningful interpretation of the results, the dataset was divided based on lead content. The resulting values were subsequently divided into two categories: samples with lead contents up to 5 wt.% (presumed to originate from non-galena copper ores) and those with lead contents exceeding 5 wt.% (likely associated with lead ores). This distinction is crucial, as it reflects two fundamentally different provenance objectives—copper versus lead ore sources—each requiring separate interpretative approaches.

## Results

### Chemical concentrations

**Fig 3:**
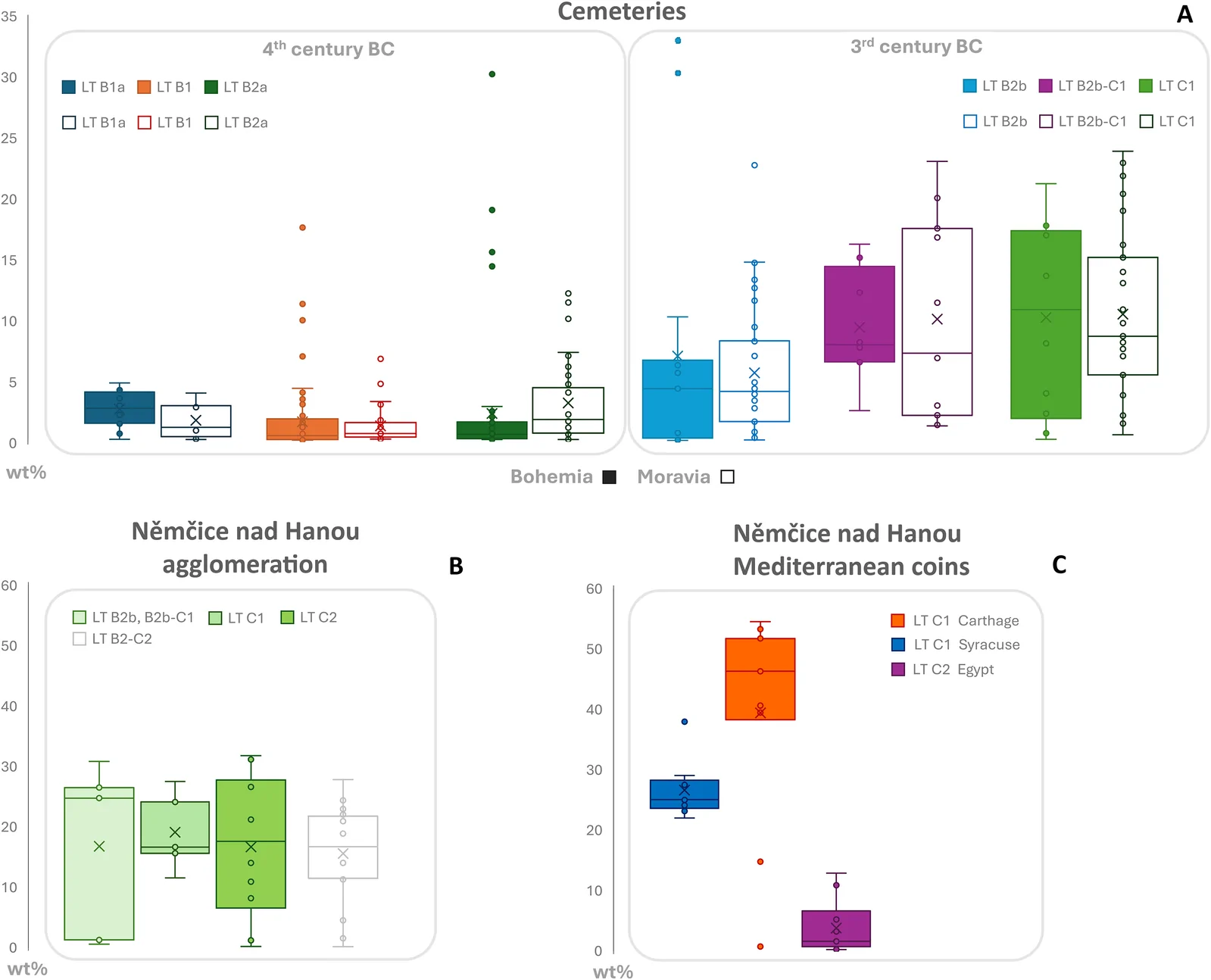
Evolution of the lead content in the bronze alloy of the personal objects in burials through the 4^th^ and 3^rd^ centuries BCE in Bohemia and Moravia (A; filled plots and symbols - Bohemia; open plots and symbols - Moravia), in the agglomeration of Němčice (B-C); and in the imported Mediterranean coins (C).

The results of the elemental analysis of the samples are presented in S1 Table. A distinct trend in the proportions of the **main alloying elements** (copper, tin, and lead) is observed in the gradual incorporation of lead into copper-tin alloys—a practice seen only in outlying samples in the later part of the 4^th^ century BCE, but becoming more systematic in the 3^rd^ century BCE [5]. The emergence of leaded bronze technology appears to have become more widespread by the very beginning of the 3^rd^ century BCE in Moravia (Fig 3.A), where a progressive increase in lead content in the alloy is evident over time. In contrast, the adoption of leaded bronze technology in Bohemia seems to have lagged behind Moravia. Highly leaded objects found in Bohemian graves were more likely imported rather than indicative of local technological expertise. However, by the second third of the 3^rd^ century BCE, the practice of casting bronze jewellery with added lead had become a well-established technological process within the study area.

The lead content of the alloys from the agglomeration at Němčice nad Hanou (Fig 3.B) was generally observed to be higher than that of objects from the cemeteries. However, no chronological trends were identified throughout the 3^rd^ century BCE, including the 2^nd^-century BCE phase, which is not contemporary with the cemeteries. The lead content of Mediterranean coins (Fig 3.C) varies: it is negligible in Ptolemaic coins, comparable to the production at the agglomeration in Syracusan coins, and significantly higher in Carthaginian coins, where the lead content regularly exceeds 40 wt%.

**Fig 4:**
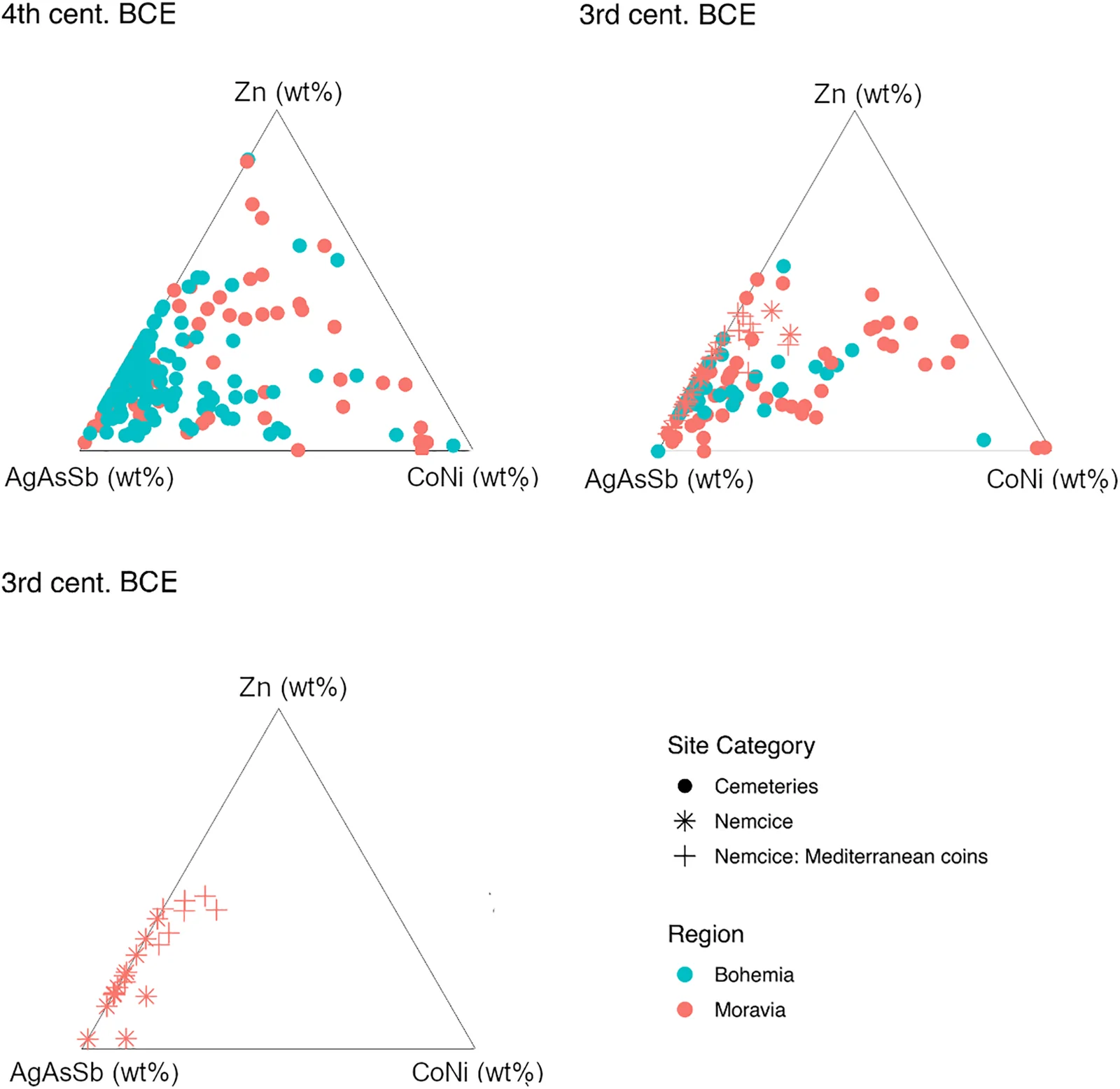
Ternary diagrams showing the relative proportions of selected trace-element groups in copper-alloy artefacts from the 4^th^ century BCE, the 3^rd^ century BCE, and the 3^rd^–2^nd^ century BCE. The ternary coordinates are defined as Σ(Ag + As + Sb) (wt.%), Σ(Co + Ni) (wt.%), and Zn (wt.%). Colours indicate region and symbols indicate site category.

In terms of classifying trace element contents based on the characteristics of potential ores, the data were assembled in several correlation matrices (Tables S1–S4 in S1 Text) and subsequently visualised in a ternary system consisting of Co, and Ni, typical for chalcopyrite ores, Ag, As, and Sb for fahlore-type ores, and Zn for galena ores (Fig 4). It is evident that the profile of the production in the 4^th^ century BCE is more heterogeneous, indicating diverse mineral sourcing, compared to the more systematic and less heterogeneous profile of the alloys in the 3^rd^ century BCE. When examining individual sites within this ternary system (Figs. 50 and 51 in S1 Text), several specific patterns emerge, such as the homogeneity of assemblages from Vliněves site in the 4^th^ century BCE or Nechvalín site in the 3^rd^ century BCE. Conversely, sites like Brno-Maloměřice, Holubice and Mlčechvosty in the 4^th^ century BCE, and Holubice, Pustiměřské Prusy, Mistřín and to some extent Němčice nad Hanou agglomeration in the 3^rd^ century BCE, tend to exhibit significant non-specificity (i.e., they are not distinctly characteristic for any mineral ore type within the ternary system).

The **PCA analysis** was run on five data matrices: a) cemeteries in the 4^th^ and the 3^rd^ century BC from Bohemia and Moravia, b) cemeteries in the 4^th^ century BCE from Bohemia and Moravia, c) cemeteries in the 3^rd^ century BCE from Bohemia and Moravia, d) Moravian cemeteries and the agglomeration of Němčice nad Hanou in the 3^rd^ century BCE, and e) Němčice nad Hanou agglomeration and Mediterranean coins in the 3^rd^ and the 2^nd^ centuries BCE. Presented here are results of runs performed on selected log-transformed trace elements. Detailed protocols on all PCA runs are provided in S1 Text.

**Fig 5:**
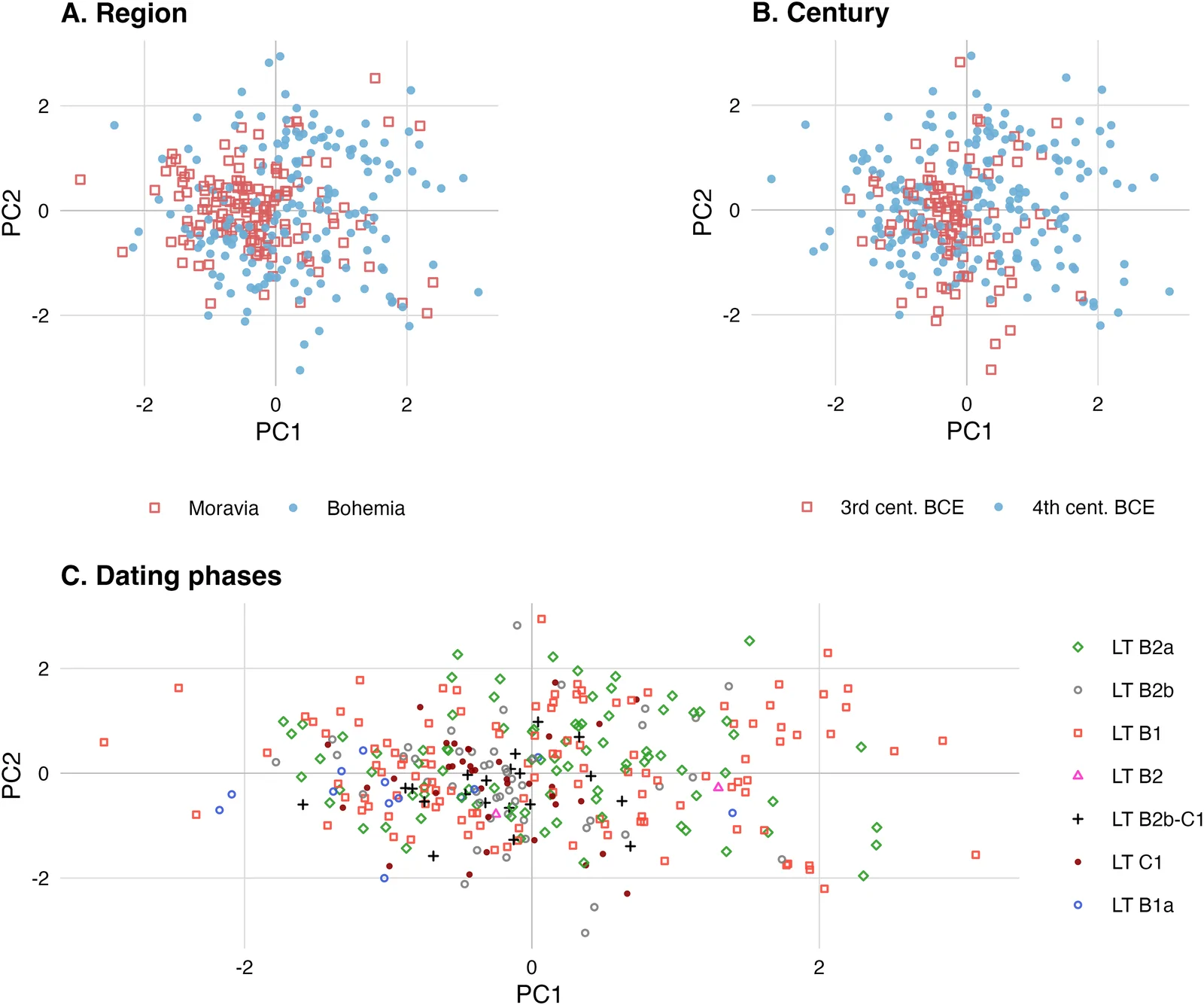
Results of the PCA analysis of the complete dataset showing basic data patterning. A – between the two main regions, B – between the two main periods, C – selected chronological phases. Site-based PCA plots for the complete dataset are provided as separate figures in Supplementary Information 2.

**Fig 6:**
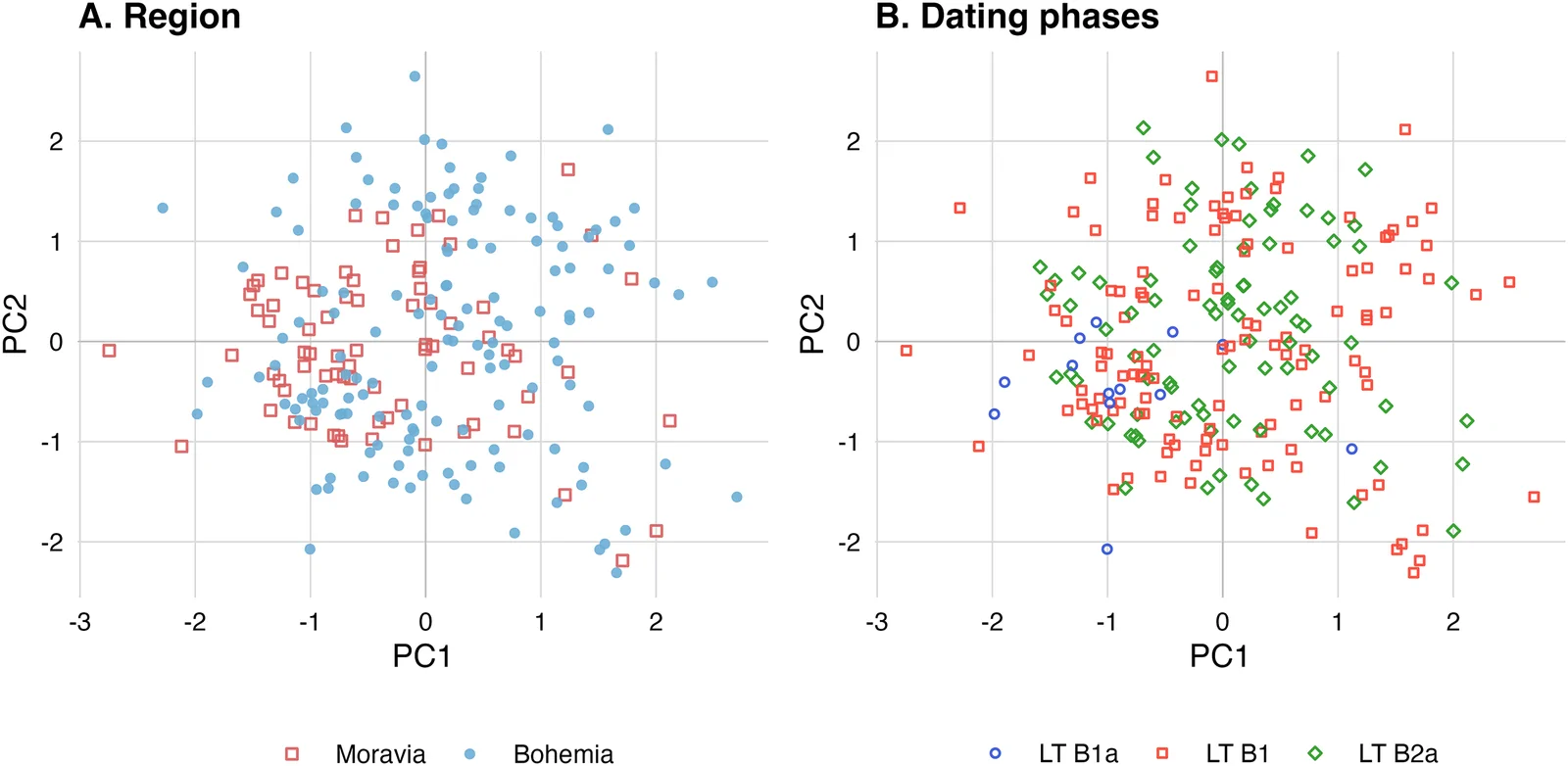
Results of the PCA analysis of the cemeteries in the 4^th^ century BCE in Bohemia and Moravia. A – pattern for the two main regions. B – selected chronological phases (LT B1a, LT B1, LT B2a). Site-based PCA plots for the 4^th^-century BCE cemetery assemblages are provided separately in Supplementary Fig. 52 in S1 Text.

**Fig 7:**
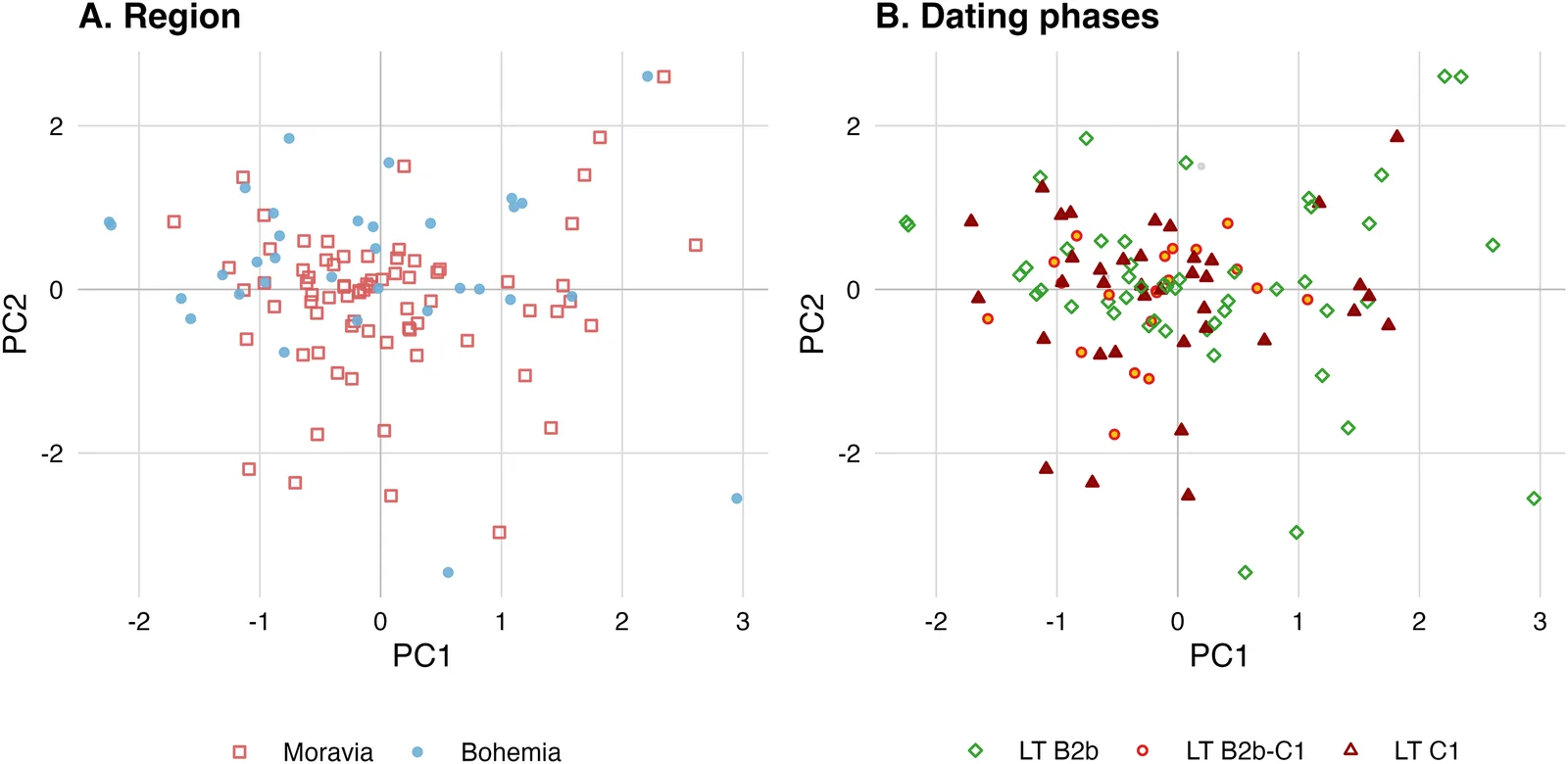
Results of the PCA analysis of the cemeteries in the 3^rd^ century BCE in Bohemia and Moravia. A – pattern for the two main regions. B – selected chronological phases (LT B2b, LT B2b-C1, LT C1). Site-based PCA plots for the 3^rd^-century BCE cemetery assemblages are provided separately in Supplementary Fig. 53 in S1 Text.

a) Analysis of the whole dataset brought forth the general information about the character of copper alloys and their differences between chronological period and geographical regions. The intermediate PC1 and PC2 values show diverse trace element profiles. Overall, the pattern points to the differences between Bohemia and Moravia (Fig 5.A), the latter showing a tendency toward a more clustered pattern. The clustering pattern is more evident also towards the 3^rd^ century BCE (Fig 5.B), especially in phases LT B2b-C1 and LT C1 (Fig 5.C). Convergent and divergent trends among individual sites from both regions are shown in separate site-based PCA Supplementary Fig. 52 and Fig 53 in S1 Text; this tendency becomes more apparent when smaller and more focused datasets were tested. Overall, the dispersion as opposed to shrinkage of samples in the PCA space suggest likely blended or regionally variable input in earlier phases, pointing to widely distributed production networks or non-specialised trade exchange developing into evidence of more homogeneous production towards the later phases.

b) The pattern for the 4^th^ century BCE in both regions shows the moderate data patterning throughout (Fig 6), possibly representing transitional, mixed or non-distinctive sources (Fig 6.B). The most pronounced differences are visible at the regional level: Moravian samples are virtually absent from the upper-right quadrant of the plot (Fig 6.A), while site-level variability is shown separately in Supplementary Fig. 52 in S1 Text. It is apparent the common features of copper alloy recipes between the large sites, such as Prosmyky and Brno-Maloměřice compared to comparatively smaller communities such as cemeteries in central Bohemia, while Moravian smaller cemeteries have the tendency of clustering along the main distribution of Brno-Maloměrice. Several samples from Prosmyky in the similar space as occupied by other Bohemian cemeteries show likely mixing of different production circles. Overal, however, the production in the earlier phase shows general unspecificity pointing to local procurement and manufacturing strategies characteristic for individual local regions that tentatively interconnect several sites together. The two larger communities, Prosmyky and Brno-Maloměřice, and their overlap possibly shows the wide distribution of supplying networks and high degree of interconnectedness visible at the scale of larger communities.
c) In the 3^rd^ century BCE, the difference between the two regions becomes most apparent. There are comparatively less samples from Bohemia, but it is apparent that the production remains in the traditional line of sources characteristic by Ag, Zn, and Bi, while in Moravia, the previous tendency towards high PCA scores for Ni, Sb, Co, and As remains similar (Fig 7.A). The clustering of Moravian sites within the moderate PCA results is lead by later sites, such as Mistřín, Hustopeče, and Brno-Maloměřice, but a very strong overlap is still apparent between the Moravian sites, specifically Nechvalín, and the site of Prosmyky in Bohemia, in that period represented only by several graves.

Divergent tendencies of individual sites, such as Ponětovice, Pustiměřské Prusy, Holubice, and the Bohemian sites of Jinonice and Vliněves, are shown separately in Supplementary Fig. 53 in S1 Text. As for dating, the PCA distribution appears to contract in the later phases represented in the figure, especially LT B2b-C1 and LT C1 (Fig 7.B).

**Fig 8:**
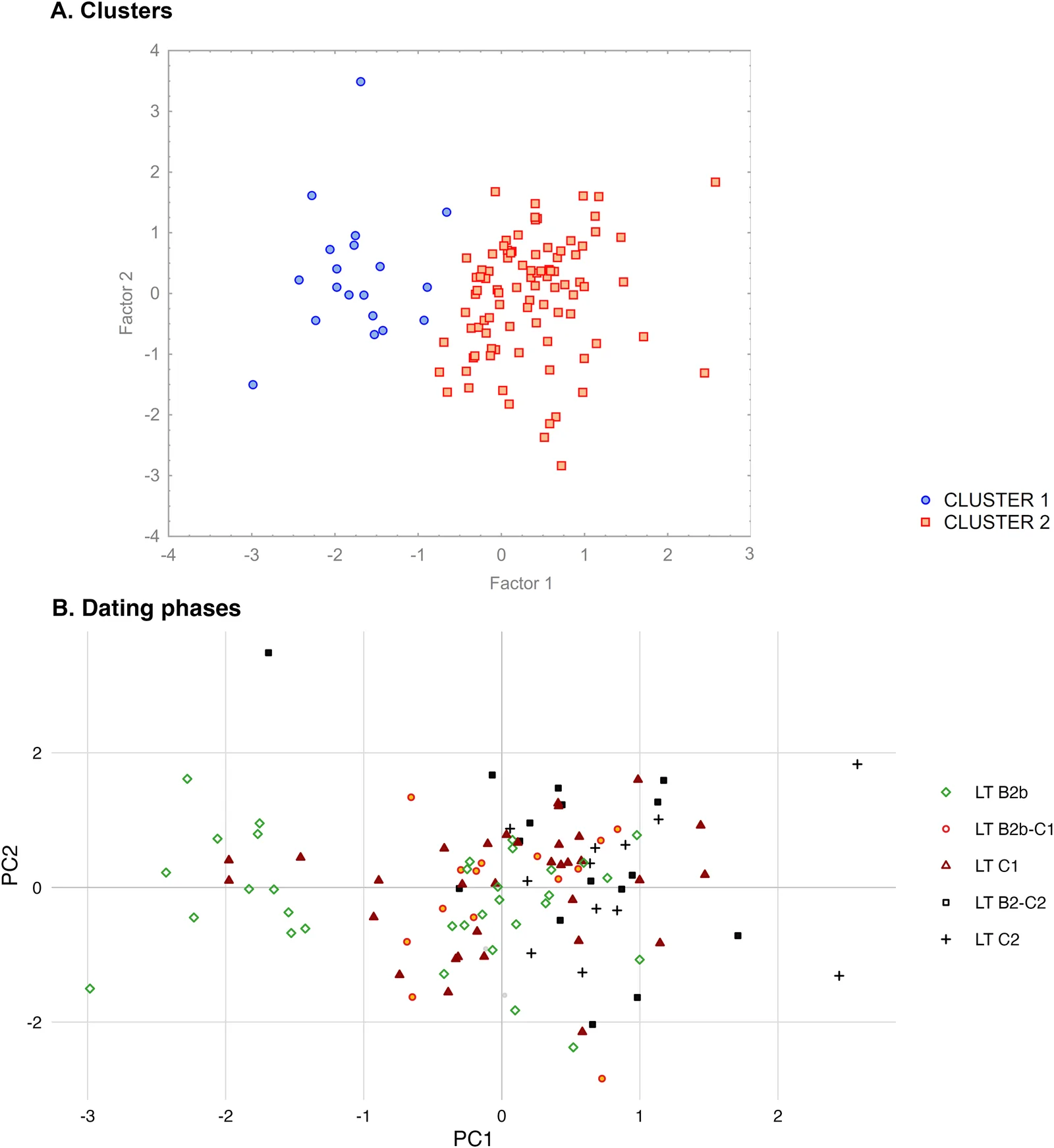
Results of the PCA and K-means clustering analyses for the cemeteries and the agglomeration of Němčice nad Hanou in the 3^rd^–2^nd^ centuries BCE. A – K-means clustering result based on PCA factor scores. B – PCA results for selected chronological phases (LT B2b, LT B2b-C1, LT C1, LT B2-C2, LT C2). Site-based and grave-level patterning is provided separately in Figs. 37–42 in S1 Text.

**Fig 9:**
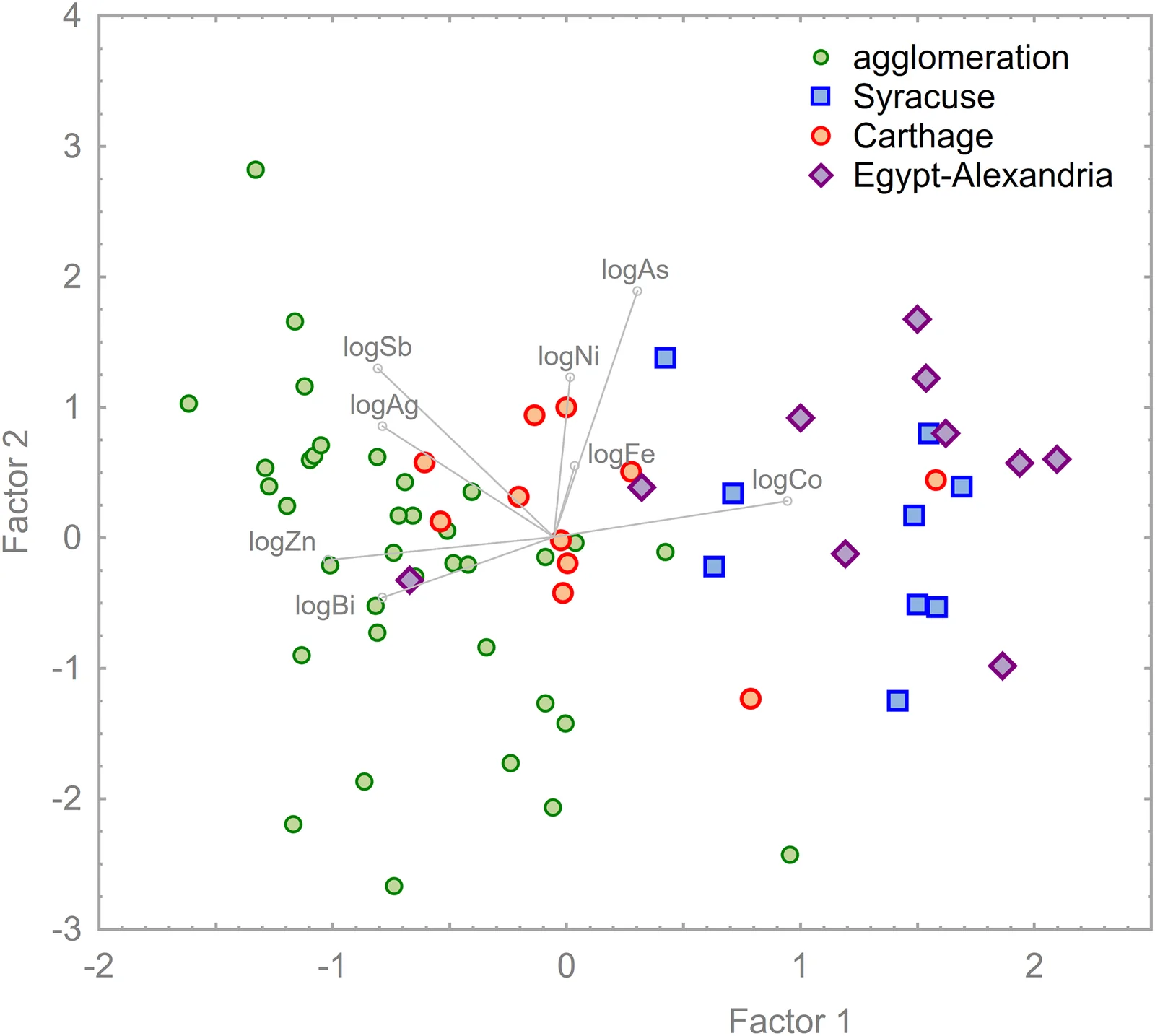
Results of the PCA analysis for the agglomeration of Němčice nad Hanou and Mediterranean coins.

d) Groupings performed for burials and the agglomeration in the 3^rd^ century BCE in Moravia identified similar significant trace elements and their correlations (Fig 8). Most of the variability along one axis is explained by the negative correlations of Ag, As, Sb, and Bi with the positive correlations of Zn and Ni. The second axis is characterised by the correlation of Co and Fe, although this relationship is less pronounced. The data are visually separated into two groups, a distinction further validated by K-means clustering (Fig 8.A), which identified two clusters as the optimal number. Within these two clusters, several patterns emerged: i) samples from the agglomeration were exclusively concentrated in cluster 2; ii) cluster 2 encompassed all dating phases, including the latest production phase occurring solely at the agglomeration, whereas cluster 1 intriguingly included only graves from the LT B2b and LT C1 phases, omitting the intermediate LT B2b-C1 phase (Fig 8.B); iii) site-based patterning is shown separately in Figs. 37–42 in S1 Text; and iv) when the resolution was refined to the level of individual graves, a clearer pattern emerged: samples in cluster 1 came from various sites (Pustiměřské Prusy, Mistřín, Brno-Maloměřice, Ponětovice, Hustopeče, Němčice nad Hanou burials and Holubice), whereas cluster 2 predominantly included samples from the same cemeteries—particularly those where sampling from multiple graves was possible (Figs. 37–42 in S1 Text).

e) The results for the analysis of the agglomeration and the Mediterranean coins indicate that the copper alloy production at the agglomeration and the imported coins did not share the same material characteristics (Fig 9). While trace elements such as As and Co are associated with the imported coins, the alloys produced at the agglomeration are characterised by Ni, Sb, Ag, and Bi. These elements are typical of the La Tène period copper industry and suggest that the Němčice agglomeration likely utilised the same copper sources as most workshops producing jewellery found in cemeteries. Some Němčice products overlap with the distribution of values for the Mediterranean coins, but a clear distinction exists between the two production circles.

### Lead Isotopes

The lead isotope (LI) systematics of studied artefacts is provided in the Supplementary Information (S2 Text) and can be summarised as follows. The value ranges for the isotopic ratios across all samples are ^206^Pb/^204^Pb: 18.14–18.83; ^207^Pb/^204^Pb: 15.55–15.68 and ^208^Pb/^204^Pb: 38.08–38.87. Due to the wide temporal range of the data and the focus of the study, we primarily consider the chronological and spatial context.

**Fig 10:**
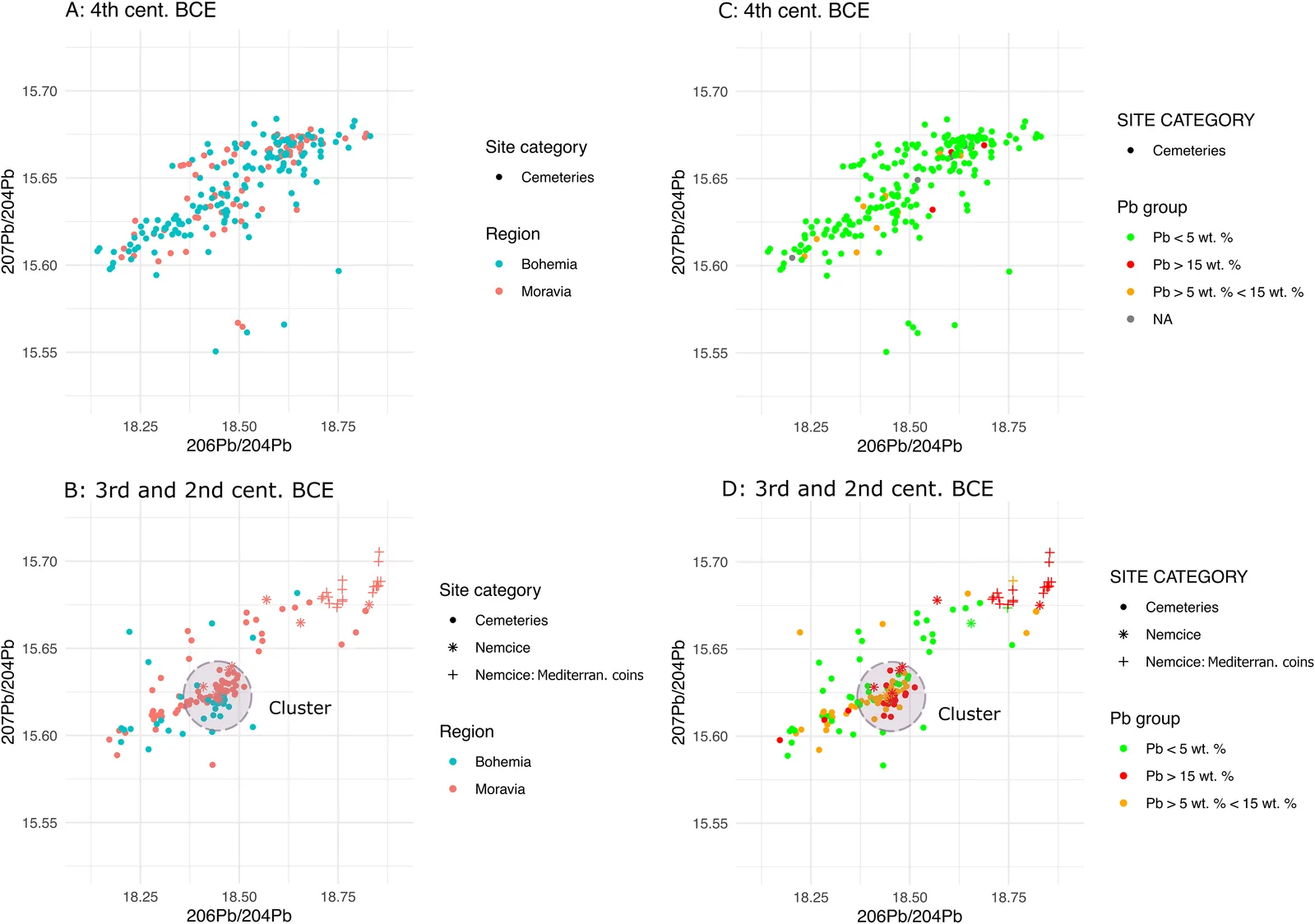
Lead isotope (^207^Pb/^204^Pb vs. ^206^Pb/^204^Pb) systematics of the studied artefacts. A–B: Lead isotope ratios for the 4^th^ (A) and 3^rd^–2^nd^ (B) centuries BCE. C–D: Same plots, with colour scale indicating lead content in wt.% of the analysed alloys (<5%, > 15%, intermediate values in between). Symbol shapes distinguish site categories (cemeteries, Němčice, and Mediterranean coins from Němčice). Purple-highlighted fields mark LI cluster discussed in the Results section.

In the chronological phases corresponding to the 4^th^ century BCE (Fig 10.A), any specific patterns in data distribution are not observed; rather, a natural trend from less radiogenic to more radiogenic values was recorded. The two regions, Moravia and Bohemia, follow more or less the same pattern. The only slightly noticeable cluster of data falls within the range of approximately ^206^Pb/^204^Pb 18.5–18.7 and ^207^Pb/^204^Pb 15.66–15.68 in the more radiogenic range suggesting older deposits of Variscan age. By the 3^rd^ century BCE (Fig 10.B), specific trends emerge. A narrower trend line among the less radiogenic values, which become more dominant, culminating in a specific LI cluster (“Cluster” in Fig 10:B) at ^206^Pb/^204^Pb 18.4–18.55 and ^207^Pb/^204^Pb 15.61–15.63 can be observed. Above a ^207^Pb/^204^Pb value of ca 15.64, the lead isotope systematics is more or less similar to that of the 4^th^ century BCE (Fig 10.A). This LI cluster is to a large extent filled with samples dated to later two thirds of the 3^rd^ century BCE.

It is evident that in the 3^rd^ century BCE, the lead isotope systematics is significantly influenced by the lead content in the artefacts, markedly exceeding the threshold of 5 wt.% (Fig 10.C-D). Beyond this level, the alloy was deliberately leaded, and the lead isotopic signatures thus correspond to lead-bearing ores. Artefacts outside the LI cluster and a trend line of the 3^rd^ century BCE only rarely exhibit lead contents exceeding 5 wt. %. Notable exceptions are Mediterranean coins, particularly those originating from Carthage or Syracuse (Fig 3.C).

**Fig 11:**
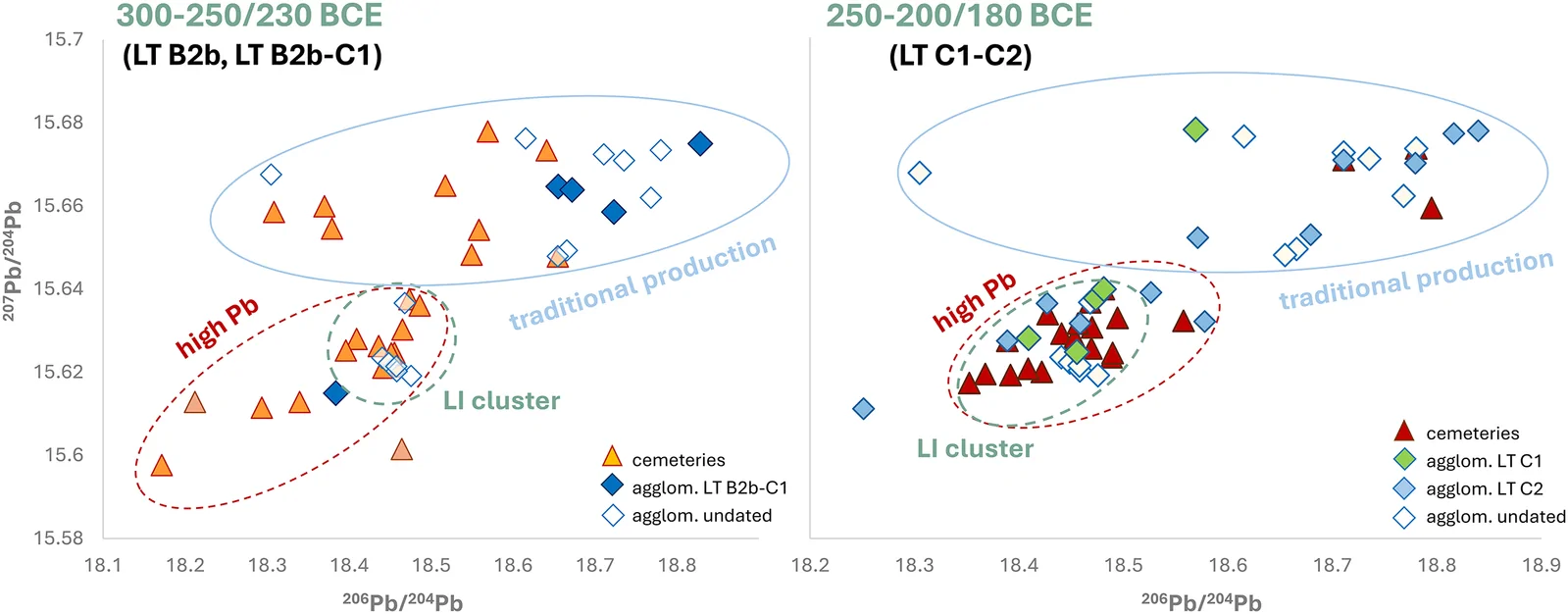
Dynamics of technological innovations at Moravian cemeteries and the agglomeration of Němčice nad Hanou. It is apparent that the agglomeration largely adopted a new source of lead (“LI cluster”) only in the second half of the 3^rd^ century BCE.

Across sites, the LI ratios organise into specific groups, pointing to differences in material provenance and source homogeneity. In the 4^th^ century BCE (Fig. 8–10 in S2 Text), large datasets such as Brno-Maloměřice, Prosmyky, and Jenišův Újezd exhibit significant heterogeneity, possibly caused by a mixed supply of raw materials. In the following century (Fig. 8, 11, 12 in S2 Text), these large datasets display much tighter clustering within the field of the LI cluster. Interestingly, these larger sites gradually exhibit greater homogeneity compared to smaller datasets from sites like Hustopeče, Holubice, Vliněves and Prague. The LI cluster appears to be more typical for Moravian sites, while the only Bohemian cemetery sharing this pattern is Prosmyky. In this respect, Moravia appears to be more isotopically unified than Bohemia.

Another phenomenon observed in the 3^rd^ century BCE is that only the datasets from Němčice nad Hanou agglomeration and Mistřín cemetery substantially exceed the distinguishing ^206^Pb/^204^Pb value of 18.55—a threshold that defines the LI cluster. These sites, along with a few samples from Holubice and Blučina, appear notably more heterogeneous in terms of lead isotope composition (Fig. 8, 11, 12 in S2 Text). In Němčice nad Hanou agglomeration, the earliest LT B2b–C1 finds fall outside of LI cluster, whereas the later LT C1 artefacts align with this isotopic field (Fig 11).

**Fig 12:**
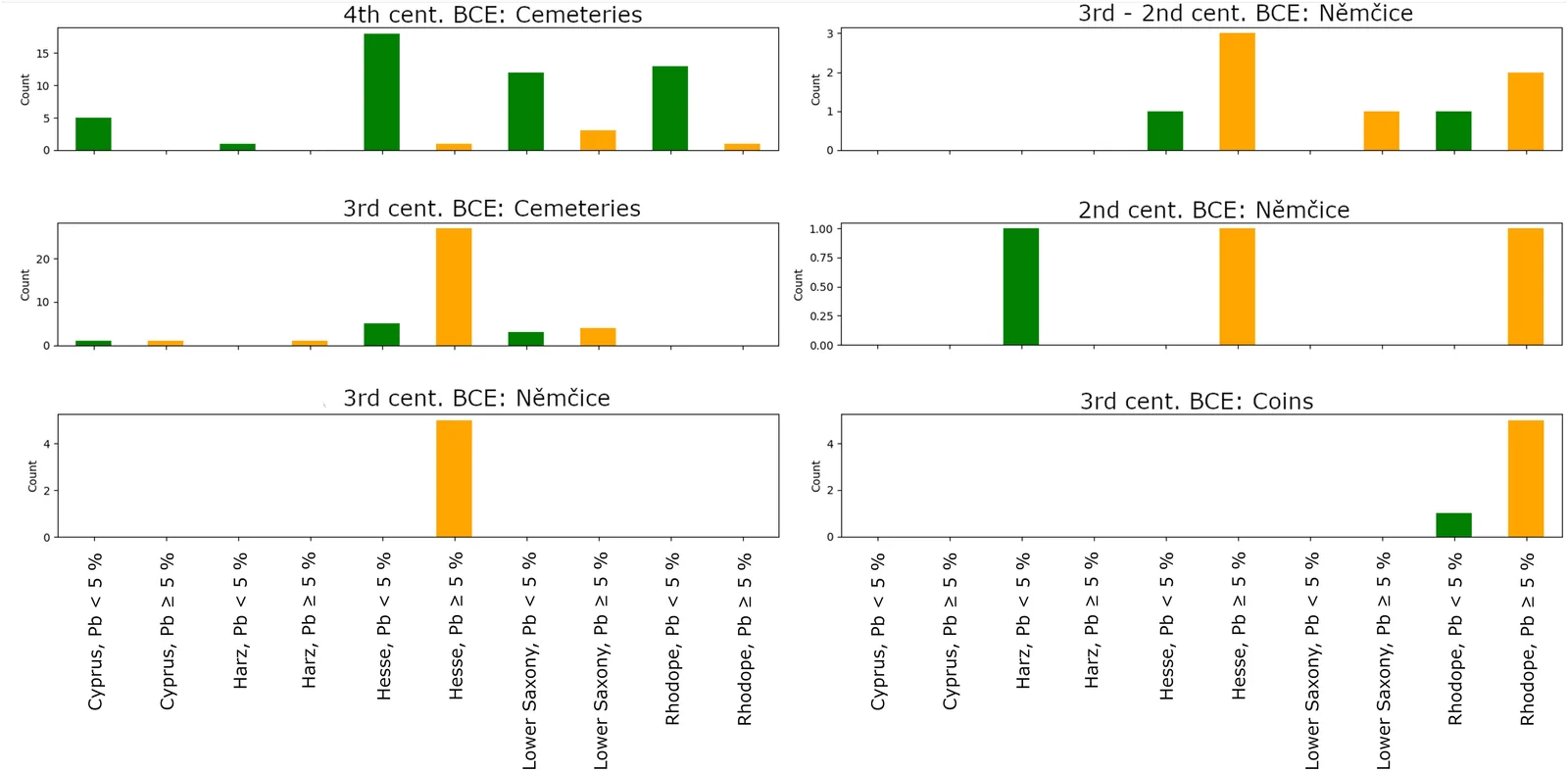
KDE based provenance analysis results with respect of the Pb amount in alloy. Green: Pb < 5 wt. %.; orange: Pb > 5 wt. %.

**Fig 13:**
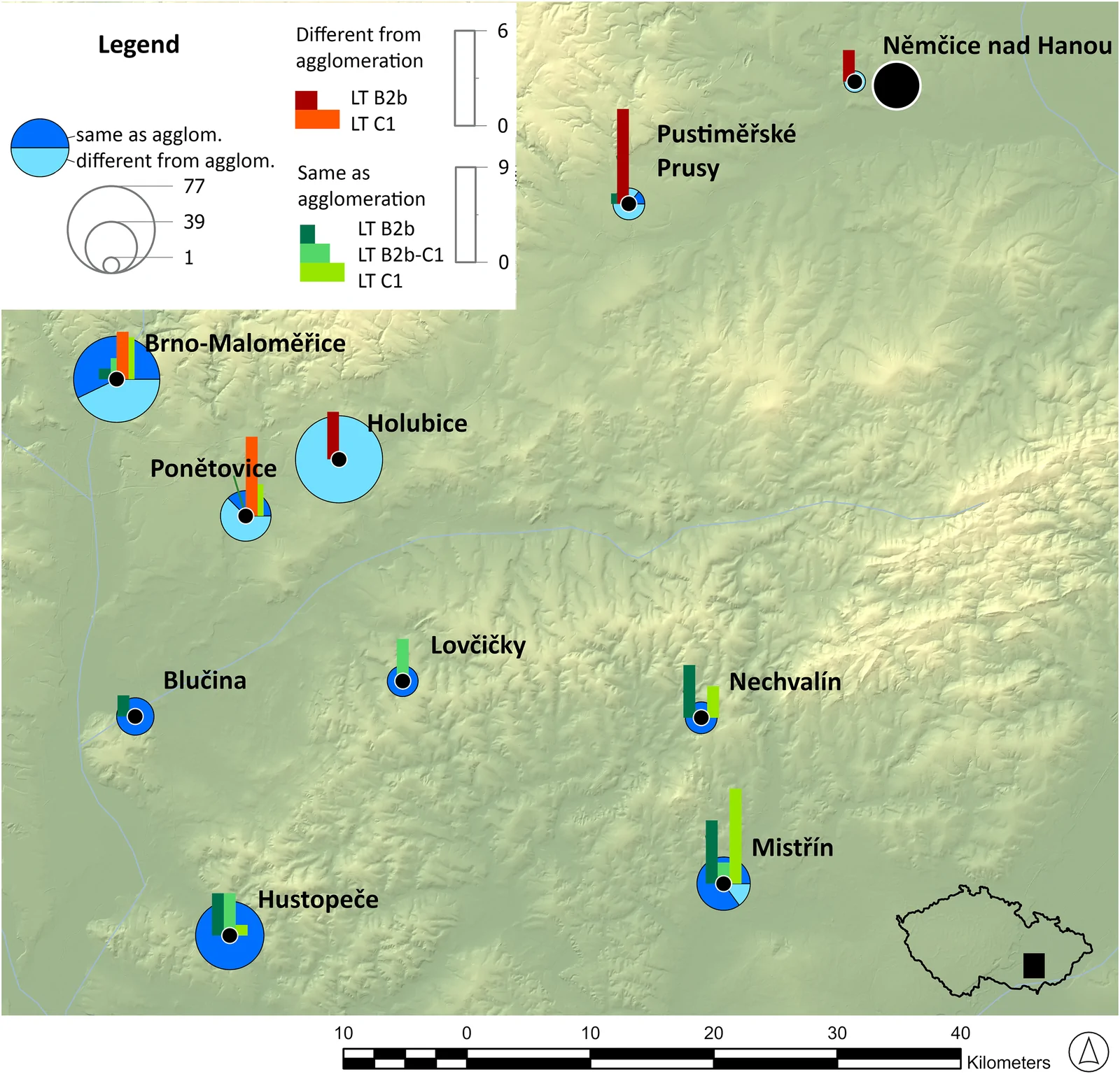
Regional patterns in the emergence of different alloy recipes, reflecting the adoption of new technological trends by cemetery communities (map created in ArcGIS Pro, background: DMR 5G Map by ČUZK, 2026, www.cuzk.gov.cz, CC-BY 4.0; graphics by A.D.).

Using the provenance model described in the methods section, following results were obtained, where individual datasets exceeded the set probability threshold of 10% for all three lead isotope ratios with ^204^Pb (Fig 12). This analysis was applied to all individually tested data subsets based on selected categories (cemeteries, the Němčice agglomeration, and Mediterranean coins, each chronologically categorised where possible). The provenance model found 87 positive matches with available ore deposits database from all 339 inspected items (ca 25% of total). For all non-Mediterranean artifacts, the highest level of consistency was observed with deposits in the Harz Mountains. Cemeteries from the 4^th^ century BCE exhibit higher source heterogeneity with only 38 matches with defined ore regions (17% of 4^th^ century BCE samples), with 34 cases linked to deposits in the Harz (18 cases), the Balkan/Rhodopes (14 cases) and Cyprus (6 cases). In the 3^rd^ century BCE, the situation became more focused, with 28 samples matching comparable ore datasets (c. 20% of 3^rd^-century BCE samples). Almost all of these matches point to Variscan ore provinces in central Europe, with 26 cases compatible with ore fields in the Harz Mountains and only two observations consistent with Cypriot deposits, although a more detailed inspection of traditional overlap plots with individual ore regions shows that several sub-provinces of the Rhenish Massif fulfil similarly strict compatibility criteria as well (Fig. 19a and 19b in S2 Text). The narrower geochemical definition of the 3^rd^-century BCE assemblages is also supported by the trace-element analysis and by the internal structure of the data. For the Němčice agglomeration, the 3^rd^-century (including the 3^rd^–2^nd^-century) material already shows a mixed but clearly Harz-dominated provenance: seven samples match Harz-type ore fields and three fall within Rhodope-type fields. The 2^nd^-century BCE phase adds one further match to each of these provinces, so that both Harz and Rhodope-related signatures are present throughout the later history of the site. However, phase-wise, detailed analysis of lead-isotope systematics from Němčice agglomeration reveals a more complex, internally differentiated artefact provenance with distinct trends. These fine-scale patterns are not captured at this level by the provenance model and are discussed in detail in the Supplementary Information (Fig. 13 and 14 in S2 Text). Rhodope-type signatures still form the single largest group (6 of 12 coins), but the updated database also points to additional Mediterranean sources in the Betic Cordillera (5 cases) and the Apennines (1 case). Overall, it is evident that in the 3^rd^ century BCE, it is possible to provenance more objects than in the preceding century. These results suggest that during the 4^th^ century BCE, metal resources exhibit a greater tendency toward source mixing. In contrast, production in the 3^rd^ century BCE reflects a more streamlined process, more likely utilising primary sources from the Rhenohercynian Fold Belt. It is evident that a significant share of the data, specifically 75%, remains without a clear provenance.

## Discussion

### Topography of technological innovations

Our results reveal a pronounced diachronic shift in the production of copper‑alloy artefacts between the 4^th^ and 3^rd^ centuries BCE across the regions studied in Central Europe. Assemblages dated to the 4^th^ century BCE exhibit wide dispersion in both lead isotopes and trace-element space. Regionally, Moravia tracks more clearly toward compositional structure in the 3^rd^ century BCE than Bohemia, perhaps reflecting earlier or stronger engagement with transforming networks that re-organised alloying and procurement of raw materials.

At the agglomeration of Němčice, early artefacts (phases LTB2b, LTB2b-C1) bear more radiogenic signatures aligned with the Alpine/Carpathian field; later LT C1 items align with the 3^rd^-century LI cluster observed at cemeteries, reflecting a transition from distinct, possibly mixed supply arrangements to the broader regional consolidation centred on a dominant lead source. The sequence is consistent with the defining third-century lead signature appearing first outside the agglomeration (Fig 11).

This diachronic consolidation is evident not only across regions but also within individual cemeteries (Figs 8, 11). Additional patterning emerges when the PCA and K-means clustering results are mapped geographically (Fig 13). Sites with a chemical composition distinct from the agglomeration at Němčice nad Hanou (K-means cluster 1) are predominantly located in the northern part of the study area, along the presumed south-north communication route. Furthermore, the earliest examples of this category (Němčice nad Hanou burials, Pustiměřské Prusy, Holubice, dating to the early 3^rd^ century BCE) are situated in the northeastern portion of this distribution, while the most recent burials (Brno-Maloměřice and Ponětovice, from the second half of the 3^rd^ century BCE) are found in the western part of the study area. In contrast, cemeteries containing materials similar to the later production at the Němčice agglomeration (K-means cluster 2) are mainly concentrated in the South Moravian lowlands and the southern part of the study area, although individual burials extend northward. One notable exception to the otherwise distinct geographical separation of clusters is Brno-Maloměřice (and to a little extent also Ponětovice), which features graves from both clusters and all chronological phases, which is possibly caused by the character and the size of the site. It means that communities represented by K-means cluster 2 were likely part of a more centralised manufacturing system, sharing access to common raw materials and technologies, with a distribution focused in the South Moravian lowlands. In contrast, K-means cluster 1 may reflect a more decentralised mode of production or reliance on different sources, evident in its spread along the presumed south–north communication axis. The presence of both clusters across all phases at Brno-Maloměřice suggests it served as a contact zone or transit hub where distinct traditions intersected and cultural flexibility was expressed. Already in the LT B2b phase, several sites show assemblages aligned with new technological trends, whereas broader production still combines these innovations with traditional lead sources. Within Bohemia, burials at the cemetery of Prosmyky is the clearest regional, albeit isolated, expression of these progressive trends (Fig. 8, 11, 12 in S2 Text). By the later 3^rd^ century BCE, communities represented in the cemeteries had largely abandoned the traditional sources and shifted to one dominant lead source (Figs 11-13). These differences could reflect the variable timing and degree of integration into shared supply networks, the weight of local recycling, or differential mobility regimes. Importantly, such heterogeneity/homogeneity is mirrored across isotopes and trace elements, strengthening the inference that genuine differences in access and practice rather than statistical noise are being observed.

Production at the agglomeration adopted this major source as well, although it continued in parallel to draw on lead from a more radiogenic isotopic zone, largely abandoned by cemeteries by this time (Fig 11), likely indicating continued mixed inputs or stronger effects of recycling and local mediation. As mentioned above, in the earliest phase at Němčice nad Hanou agglomeration, the adoption of leaded alloys is not apparent; rather, leaded bronze production expanded later as metallurgical production scaled up to meet the increased demand. This timing is evidence of an emerging central-place role consolidating throughput—storage, brokerage, and high-volume casting—after the shift was already underway in surrounding communities. The results challenge a simple model in which agglomeration sites originate technological change later seen in rural communities. Instead, funerary contexts, representing broader community practices, register the transition from flexible, mixed 4^th^-century production to more structured 3^rd^-century alloying, with agglomerations subsequently amplifying and scaling these shifting regimes. It should be noted that the production spectra of the cemetery communities and the agglomeration largely overlapped. In both contexts, copper-alloy metalworking was primarily directed towards the manufacture of jewellery, personal ornaments, and decorative objects more broadly. The earlier phases of production at the agglomeration provide evidence for the casting of elaborate costume components, including decorative chain belts, which are also represented in contemporary burials. Thus, although differences between funerary and settlement contexts may complicate direct comparison of the technologies employed, the same categories of objects occur in both environments. Even in cases where contemporaneous cemetery jewellery does not fully mirror agglomeration outputs, the results imply partially overlapping but distinct workshop networks and procurement channels. Glass production and coinage, hallmarks of later agglomeration specialisation [6] postdate the alloying transition documented in cemeteries already about one or two generations earlier.

The disparities between Bohemia and Moravia are best explained by their geographic positions and shifts in the orientation and organisation of local networks. During this period, the Amber Road and Middle Danube corridors rose in importance, while Bohemia—lying farther from these routes—functioned largely as a transitory zone linking distant raw-material sources to the west. Only larger communities organised around centralised hubs, such as Prosmyky, were able to track and adopt emerging innovations. The same socio-economic pattern appears in other domains, notably diet: gains in agricultural efficiency were concentrated in the Middle Danube area and were taken up only by selected large communities in Bohemia [34].

### Provenance of the raw materials

Provenance analysis with very large comparative datasets risks uneven spatial representation of ore provinces and an illusion of objectivity if matches are taken at face value. Three confounding processes merit consideration. First, recycling can re-average signatures; however, the tight 3^rd^-century clustering across both isotopes and trace elements is difficult to produce through recycling alone and instead suggests consistent inputs and recipes. Second, mobility might seed non-local signals in otherwise local assemblages; yet the systematic nature of 3^rd^-century convergence (and the weak Mediterranean copper-alloy signal) implies that long-distance movements did not dominate the procurement underpinning mass leaded bronze production. Third, sampling intensity varies by site; therefore, the aim in this study was to refrain from over-interpreting single outliers and to emphasise patterns replicated across multiple cemeteries and contexts. The approach presented in this study mitigated these issues by combining a KDE match/no-match framework with traditional bivariate distribution analysis and by cross-checking isotopic inferences against trace-element behaviour and archaeological context [1,5,20]. This triangulation increases confidence in (a) a multi-source, flexible 4^th^-century regime and (b) a 3^rd^-century consolidation around Rhenohercynian ore provinces for the lead component—while acknowledging inevitable blurring from recycling and mixing. However, it is essential to take into account that the model does not provide a single definitive explanation of provenance but primarily serves as a tool for working with the vast comparative database available today.

The comparisons of assemblages against geological fields show two principal isotopic “domains” (Fig. 15a–17c in S2 Text). Less radiogenic signatures (≈^206^Pb/^204^Pb < 18.55) overlap with nearby Transalpine provinces (Bohemian Massif, Rhenish Massif, Harz Mountains) and typically present lower As–Sb–Ag with a mild propensity for Zn. More radiogenic signatures (≈^206^Pb/^204^Pb > 18.55) overlap with younger Alpine/Carpathian belts (Balkan/Rhodopes; Valais; Apuseni/Slovakia) and often show higher Ag and Sb, consistent with a fahlore-type component. These relationships parallel previously observed clusters in the 4^th^ century BCE Duchcov hoard (Cluster 3 ≈ Bohemian Massif/Erzgebirge; Cluster 1 ≈ western Alps) [1], suggesting that the earlier craftsmen drew on multiple ore inputs, signalling mixed resources, flexible alloying, and a lack of consistent supplies or recipes—conditions consistent with small-scale and locally mediated production, aligning with the prevailing (often implicit) model of Iron Age organisation of the production. The emergent pattern in the 3^rd^ century BCE, especially observed in Moravian cemeteries and the Prosmyky site in Bohemia, includes a characteristic reliance on a major consistent lead source (LI cluster). In geological terms, the source regions of the LI cluster can be situated within the Rhenohercynian Fold Belt, in particular parts of the Rhenish Massif and the Harz Mountains. This Variscan fold-and-thrust belt is dominated by Devonian–Carboniferous sedimentary successions, within which late Variscan to Permian hydrothermal circulation produced significant Pb–Zn–Ag vein and stratiform mineralisation (regional overviews: [35,36]). A detailed comparison of artefact Pb isotope compositions with these ore fields indicates the closest matches with hydrothermal vein systems in the Taunus segment of the Rhenish Massif (recorded as “Hesse” in our dataset) and with deposits in the Harz (Fig. 19a and 19b in S2 Text) [35,37–42]. Previous Pb isotope analyses of rouelles (spoked wheel amulets), beads and other La Tène-period lead objects from the Wallendorf site show that their compositions fall within the Variscan and post-Variscan deposits of the Rhenish Massif, leading to the assumption that Roman exploitation of Eifel lead largely continued an already established Celtic mining tradition [35]. Taken together with results in this study, these data are consistent with a long-lived lead-supply system centred on these polymetallic deposits, plausibly operating from at least the 3^rd^ century BCE and persisting into the 1^st^ century BCE and the early Roman period, thereby extending and strengthening the hypothesis of pre-Roman Pb procurement. At present, there is still no direct evidence for Iron Age extraction of the Pb–Zn–Cu sources in the region of Rhenish Massif and Harz. Nevertheless, iron-smelting furnaces are documented in the neighbouring Siegerland region for La Tène B2 (middle of the 3^rd^ century BCE), together with tentative indications of copper smelting [43]. Further evidence for intensive Iron Age exploitation in the Rhenish Massif is linked to the salt economy: at the eastern margin of the Taunus, the large salt-evaporation complex at Bad Nauheim documents sustained salt production from at least the 3^rd^ century BCE, i.e., contemporaneous with the lead procurement regime considered here [44]. If we reconsider the potential long-distance connections linking these mining and salt-producing zones to Bohemia, salt again offers an obvious candidate vector: earlier work on La Tène communication routes has envisaged salt being brought to Bohemia from Saxony, usually hypothetically located in the Halle/eastern Harz region [45,46], even though explicit archaeological evidence for salt production in central Germany between the 4^th^ and 1^st^ centuries BCE is largely lacking [47]. In light of the new isotopic and archaeological evidence presented here, it is suggested that, in addition to the salt transportation, the same corridors may also have channelled Pb-bearing materials derived primarily from Rhenish Massif deposits, providing a new hypothetical linkage between Bohemia and the Rhenish Massif (with a possible, though less likely, contribution from the eastern Harz region). If we complement the archaeometric signal with the currently documented pattern of extractive and industrial activity, the balance of probability between these alternatives favours the above discussed deposits in the Rhenish Massif rather than the Harz as the principal Pb source.

Despite increased contact with the Hellenistic world, copper-alloy data show only sparse matches to Greek/Macedonian provinces, and those are mostly confined to 4^th^-century items (e.g., few Balkan/Rhodopes matches). In geological terms, the “Rhodope” matches identified by the provenance model point to the Tertiary polymetallic Pb–Zn–Ag (±Cu ± Au) ore systems of the Thrace–Rhodope belt—hydrothermal vein, replacement and epithermal deposits hosted in the metamorphic core complex of the Rhodope and Serbomacedonian massifs [27,28,48–51]. Mediterranean coins display isotopic signals distinct from southern copper-alloy production, however, there is no evidence of them being used as a material source intended for recycling (Fig 9). In other words, the widespread 3^rd^-century adoption of leaded bronze in Central Europe is not anchored in Mediterranean metal flows; rather, Transalpine ore fields, especially the Rhenohercynian Fold Belt, dominate the provenance of the lead component that enabled the increased production of the Plastic-Style jewellery. This observation matters for broader debates on technological transmission. If the mechanism had been import-driven through Mediterranean artefacts, earlier objects would prominently carry Mediterranean signatures. Instead, present dataset favours a model in which stylistic ideas and certain technical concepts travelled widely, but the material substrate (ores) for Central European mass production derived predominantly from Transalpine sources.

### Socio-economic and cultural implications of emergent leaded bronze production

Our results reveal that the selection of leaded alloys was a deliberate and selective process, employed when the jewellery shape and decorative requirements necessitated a material with specific properties. In contrast, more common items, such as brooches, do not show evidence of leaded alloys. This indicates that bronze workshops had mastered multiple techniques and that craftsmen made intentional material choices based on the functional and aesthetic demands of the fabrication process. Consolidation around few lead sources and alloy recipes implies organised access and control of supply/distribution for specific markets, connected with the onset of the popularity of Plastic-Style jewellery. Lead’s lower price relative to tin [14] reduced unit costs for large, voluminous ornaments, enabling a surge in robust jewellery forms in the course of the 3^rd^ century BCE. The result is a compelling fit between alloy properties, cost structure, and stylistic demand. From the visual perspective, the new, attractive jewellery made in the Plastic-Style was significantly more robust and likely functioned as an important status symbol of the new, economically and perhaps even politically more established and self-confident Iron Age society of the 3^rd^ century BCE. The idea of a new artistic style and crafting probably originated in the Mediterranean, while the material used for these artistic manifestations had a local origin, independent of the Mediterranean world. Gradually, with the growing demand for new jewellery designs made from new materials, this development became fertile ground for social stimulation. The demand for new materials largely increased in volume which in turn created an opportunity to be seized by individuals or groups connected to long-distance socio-economic networks, possibly fostered by previous military campaigns. Archaeological evidence points to local warrior elites, possibly the heirs of 4^th^-century BCE non-centralised, rural based society, as the most likely enforcers of the new economic opportunities that has given rise to influx of new, much more conspicuous bronze ornaments.

We interpret the new leaded bronze jewellery (and, shortly thereafter, also newly introduced glass, and re-introduced amber ornaments) as archetypal “*bulk luxury commodities*” that encode prestige through form and style, yet are sufficiently affordable, thanks to standardised inputs and scalable craft, to diffuse broadly [52–54]. Archaeometrically, 4^th^-century bronzes read as heterogeneous, locally mediated, and weakly coordinated utilitarian goods; by contrast, 3^rd^-century leaded bronzes combine (i) prestige coding (Plastic-Style and its characteristic bulkiness), (ii) concentrated sourcing (narrow Pb isotope field, and similar alloy recipes), and (iii) standardised distribution enabled by cost advantages—all the hallmarks of bulk luxury goods.

Several comparative analogies strengthen this reading. First, Bohemian sapropelite jewellery [55] represented a locally abundant material entering wide networks via distinctive, fashionable forms, similarly fostering elite brokerage over the flow and distribution rather than exclusive control of rare inputs. Together with early leaded bronze ornaments, it is one of the earliest archaeologically visible indicators of this process, rooted in locally or regionally available, non-Mediterranean resources. Second, the late 3^rd^ century BCE spread of glass ornaments expresses analogous standardisation and regional integration of a commodity with both exotic connotations and wide availability [56]. Rolland’s analysis of Late Iron Age glassmaking shows that production was closely tied to rising demand from newly emerging economic elites – the *homines novi* of the later 2^nd^ century BCE – highlighting the transformative power of bulk-luxury goods [57].Third, although amber is not systematically attested within the cemetery communities examined here, it appears to have gained renewed popularity through central places, as recently confirmed by evidence for amber-working at agglomerations such as Němčice nad Hanou [10]. Amber therefore represents a useful example of a bulk-luxury commodity curated through organised networks of supply, production, and distribution. Raw amber was imported from northern source regions and transformed into desirable finished products in workshops located at Middle Danube agglomerations. This pattern was later continued and further developed within the trade and production systems of the oppida [58]. The renewed prominence of amber may thus reflect an intensification of connections along the north–south axis, represented by the Amber Road, which complemented the already developed west–east routes used for the movement of metals, salt, and other commodities. Finally, coinage made of precious metals is another example of a comparably standardised system that emerged during the 3^rd^ century BCE, reflecting broader shifts toward technological cohesion, material integration, and economic coordination, however based on different networks of sourcing and production [6]. These bulk-luxury goods formed an intermediary category: costly enough to mark prestige yet widely accessible, combining aesthetic capital with broad reach and thus enabling new modes of distribution, regional market consolidation, and the emergence of new elite configurations.

Central places, including Němčice nad Hanou, are best viewed as throughput hubs within an already changing landscape, scaling up production, buffering supply fluctuations, and exporting recognisable stylistic packages across regional networks. Operating as gateway nodes on long-distance routes, these sites used control over raw-material access, transit points, storage, and brokerage to turn flows of leaded bronze and, later, glass, amber and precious metals for coinage into stable rents and market power. As prestige-coded but widely accessible goods scaled up, the focus of elites shifted from monopolising rare materials to coordinating flows, crafting, and distribution at volume. This elite input in consolidating the production and distribution flows of bulk luxury goods was tightly connected with social cohesion, cultural identity, and symbolic expression, reconfiguring thus how we understand Iron Age markets and, in a broader sense, geopolitical integration of the whole regions. Crucially, at the onset of this process, when leaded bronze first entered the markets, agglomerations had not yet formed. Instead, relatively independent communities, represented by their cemeteries, secured high-volume, provenance-specific goods, while the later rise of agglomerations appears to have increased the efficiency and liquidity of these flows. Communities around cemeteries were not passive repositories of elite fashion; rather, they registered early, community-wide shifts in alloying and sourcing that preceded – and then fed into – agglomeration-based scaling. This perspective helps explain why cemetery assemblages partially overlapped with agglomeration inventories: their workshops were connected but not identical, and their procurement was integrated yet diversified. In turn, the cemetery record captures early adoption among households and kin groups, while central places optimised the production only about a generation later. This model aligns with later developments in the 2^nd^ and 1^st^ centuries BCE when late La Tène and early Germanic polities, based again chiefly in Bohemia, capitalised on extensive external supplies, as evidenced by connections to material flows from Hispania [59,60], or later making use of Roman-supplied raw materials to produce their own prestige goods [21]. This highlights durable Central European capacity to translate external connections into standardised, high-demand commodities.

## Conclusion

Material analysis of personal objects offers a unique opportunity to provide multi-faceted evidence on technological change and its social consequences. Archaeological contextualisation, encompassing chronological, typological, and geographical details, along with decoration styles, plays a crucial role in the informed interpretation of geochemical data, which reveals material composition, provenance, and technological aspects of the objects. The data patterns derived from these analyses are invaluable for identifying and interpreting societal evolution, particularly through the lens of socio-cultural expression. In our case, they show how technologies and know-how spread along developed socio‑commercial links between communities, embedded in increasingly complex forms of social organisation and likely coordinated, at least in part, by originally rural elites.

The widespread adoption of leaded bronze—facilitating the mass production of ornamentation within the category of bulk luxury—reflects a shift from dispersed, decentralised craft to more centralised, economically embedded regimes of resource control. This reorganisation contributed to the emergence of new social elites, no longer defined primarily by martial symbolism but by their access to materials, production capacity, and distribution. Comparable dynamics can be observed in the early implementation of other strategic materials, such as glass and precious metals.

Geochemically, this transformation is visible in the move from heterogeneous, multi‑source 4^th^‑century alloys to the more clustered, deliberately leaded bronzes of the 3^rd^ century BCE. Isotopic data and PCA analysis indicate that most 3^rd^-century ornaments cluster within a narrow, less radiogenic lead-isotope field, consistent with Rhenohercynian ore provinces. In contrast, more “external” inputs—such as Mediterranean coin-derived lead recorded in the Němčice nad Hanou assemblage—plot as a separate group, suggesting they were not recycled at scale. Within this transition, rural funerary assemblages register the new regime earliest: communities linked by the Amber Road corridor share increasingly similar alloy recipes, while agglomerations such as Němčice nad Hanou subsequently concentrate and amplify these trends rather than initiating them. The analysis presented here thus repositions communities of rural cemeteries as key agents of technological innovation and bulk‑luxury circulation in the Middle Danube area, with central places operating as throughput hubs in an already transformed economic landscape, amplifying these community-level innovations by concentrating production, storage, and distribution.

Alltogether, the geochemical, technological, and contextual strands converge on a model in which the 3^rd^ century BCE marks a decisive move from locally flexible metallurgy to regionally coordinated regimes. Leaded bronze, by aligning performance (castability), cost (lead cheaper than tin), and fashion (Plastic-Style), created a commodity category with unusual social power: a prestige-coded but mass-circulating good. This, in turn, fostered (i) more predictable supply chains for key inputs (notably Rhenohercynian Fold Belt lead), (ii) standardised craft routines, and (iii) market formation around recognisable styles—each of which reinforced new forms of elite authority rooted in systematically managing flows rather than focusing on rarities. In this sense, leaded bronze jewellery represents one of the earliest archaeologically visible, and now analytically well documented, manifestations of the politically and economically transformative force of bulk-luxury commodities in Iron Age Central Europe.

Within this frame, Middle Danube area, and more specifically Moravia, appears as an early and dynamic participant: leaded bronze technology is visible already by the early 3^rd^ century BCE, and the whole region later becomes a crucible for other “cohesion” technologies (coinage, glass), even if the alloying standardisation detected in cemeteries was not originally agglomeration driven. The cemetery/agglomeration sequencing that is documented here thus refines how the emergence of Central European industrial open agglomerations is narrated: rather than singular engines of innovation, they are accelerators and integrators of trends seeded originally within communities.

Methodologically, the synthesis of KDE-based matching, conventional isotopic plots, trace-element structure, and archaeological context allows us to separate broad, repeated patterns from site-specific noise and to resist simplistic import-driven models of technological change. Substantively, the results speak to how materials and recipes participate in building markets and reshaping authority during the later part of the Iron Age.

## Supporting information

S1 Text Supplementary Information 2: PCA and K-means cluster analyses. (DOCX)

S2 Text Supplementary Information 3: Isotopic analysis. (DOCX)

S1 Table Analytical dataset. (XLSX)

S2 Table Lead Isotope Database of Ore Fields. (XLSX)

S1 Code Statistical evaluation workflow of lead isotopic data. (PDF)
