# Supplementary material for: Tracing socioeconomic change and emerging elites in 4th–3rd century BCE: Metalwork from the Amber Road Corridor
Source: PLoS One. 2026 Sep 2;21(9):e0352885. doi: 10.1371/journal.pone.0352885 (PMC13537607; doi:10.1371/journal.pone.0352885)
Supplement: Supplementary file 1 [file pone.0352885.s005.pdf]

```
"""
Python implementation of a kernel density–based lead isotope provenance model
after De Ceuster & Degryse (2020, Archaeometry).
For each mining district (n ≥ 20), kernel density estimates are constructed for
206Pb/204Pb, 207Pb/204Pb and 208Pb/204Pb ratios. The probability density is
integrated over a fixed interval around each artefact value. Relative
probabilities are compared across regions and combined using the geometric
mean to identify the most probable source region within the reference dataset.
You can call the function with comand: model_pipeline(passed_df=fixed_coding, coding="CODE",
layer="General Unit", threshold=0.1)
"""
import os
import pandas as pd
import numpy as np
import matplotlib.pyplot as plt
from scipy.stats import gaussian_kde
from scipy.integrate import simpson
from scipy.stats import gmean
from collections import Counter
base_dir = os.path.dirname(os.path.abspath(__file__))  # Get the absolute path of the current script
training_data_dir = os.path.join(base_dir, 'training_data', """"YOUR COMPARISON DATASET"""") #
Finish the path with your dataset
outputs_dir = os.path.join(base_dir, 'outputs')
def model_pipeline(user_data_path=None, data_sheet=None, passed_df=None, coding=None,
layer=None,
                grouping_column=None, threshold=None, title="Artifacts"):
    if user_data_path:
        user_data_df = pd.read_excel(user_data_path, sheet_name=data_sheet)
    elif passed_df is not None:
        user_data_df = passed_df
    else:
        raise ValueError("Either 'user_data_path' or 'passed_df' must be provided.")  # Added for
robustness.
    user_data_df.columns = user_data_df.columns.str.strip()
    if coding in user_data_df.columns:
        user_data_df.rename(columns={coding: "CODE"}, inplace=True)
    else:
        print(f"Warning: Column '{coding}' does not exist in the dataset, 'CODE' will not be updated.")
    all_data = pd.read_excel(training_data_dir, sheet_name="SHEET NAME") # The name of the sheet
you are working with
    all_data = all_data.iloc[:, 2:]
    if grouping_column:
        unique_groups = user_data_df[grouping_column].dropna().unique()
    else:
        unique_groups = [None]  # Placeholder for no grouping
    all_results_table = []
    for grouping_value in unique_groups:
        if grouping_value is not None:
            print(f"\nProcessing group: {grouping_value}")
            group_data = user_data_df[user_data_df[grouping_column] == grouping_value]
        else:
            print("\nProcessing the entire dataset")
```

```
    group_data = user_data_df
# Reset variables for each group
# Stored for optional downstream analysis
probability_data = []
mean_results = []
group_names_all = []
all_results = []
for index, row in group_data.iterrows():
    x_206 = row["206Pb/204Pb"]
    x_207 = row["207Pb/204Pb"]
    x_208 = row["208Pb/204Pb"]

    # Ensure numeric data and filter finite values
    columns_to_numeric = ['206Pb/204Pb', '207Pb/204Pb', '208Pb/204Pb']
    for col in columns_to_numeric:
        all_data[col] = pd.to_numeric(all_data[col], errors='coerce')
    all_data = all_data.dropna(subset=columns_to_numeric)
    # Filter for each region
    regions = all_data[layer].dropna().unique()
    region_data = {region: all_data[all_data[layer] == region] for region in regions}
    # Apply length condition
    region_data = {region: data for region, data in region_data.items() if len(data) >= 20}
    # Integrate KDE over specific ranges
    def integrate_density(data, column, bw, x_range):
        if data.empty:
            return 0
        kde = gaussian_kde(data[column], bw_method=bw)
        x = np.linspace(x_range[0], x_range[1], 500)
        y = kde(x)
        return simpson(y, x=x)
    integration_ranges = {
        "206Pb/204Pb": (x_206 - 0.01, x_206 + 0.01),
        "207Pb/204Pb": (x_207 - 0.01, x_207 + 0.01),
        "208Pb/204Pb": (x_208 - 0.03, x_208 + 0.03),
    }
    percentages = {}
    for isotope, x_range in integration_ranges.items():
        percentages[isotope] = [
            (region, integrate_density(region_data[region], isotope,
                                       bw=0.15 if isotope == '206Pb/204Pb' else
                                         (0.008 if isotope == '207Pb/204Pb' else 0.03),
                                       x_range=x_range))
            for region in region_data.keys()
        ]
    # Combine percentages into a table
    valid_regions = list(region_data.keys())
    percentages_table = pd.DataFrame(percentages, index=valid_regions)
    print(percentages_table)
    probability_data.append(percentages)
    CODE = row.get("CODE", f"Obs_{index}")
    all_groups = set()
    isotope_probabilities = {}
```

```
            for isotope, group_probs in percentages.items():
                isotope_probabilities[isotope] = dict(group_probs)
                all_groups.update(group for group, _ in group_probs)
            filtered_groups = []
            for group in all_groups:
                if all(isotope_probabilities[isotope].get(group, 0) > threshold for isotope in
isotope_probabilities):
                    filtered_groups.append(group)
            if not filtered_groups:
                continue
            group_means = []
            for group in filtered_groups:
                probs = [isotope_probabilities[isotope][group] for isotope in isotope_probabilities]
                geom_mean = gmean(probs)
                group_means.append((group, geom_mean))
            best_group, max_mean = max(group_means, key=lambda x: x[1])
            mean_results.append(max_mean)
            group_names_all.append(filtered_groups)
            all_results.append((best_group, CODE))
            all_results_table.append((best_group, CODE))
        group_counts = Counter([group for group, _ in all_results])
        # Illustrative visualization of assignment counts
        for group, code in all_results:
            print(f"Group: {group}, Observation: {code}")

        plt.figure(figsize=(10, 6))
        bars = plt.bar(group_counts.keys(), group_counts.values(), color='skyblue')
        for bar in bars:
            height = bar.get_height()
            plt.text(bar.get_x() + bar.get_width() / 2, height, str(int(height)),
                    ha='center', va='bottom', fontsize=10, color='black')
        plt.xlabel('Group')
        plt.ylabel('Number of Assignments')
        plt.title(f'{layer} per {grouping_column} - {grouping_value}' if title == "" else title)
        plt.xticks(rotation=90)
        plt.tight_layout()
        plt.show()
    results_df = pd.DataFrame(all_results_table, columns=["Estimated Group", "CODE"])
    results_df.to_csv(os.path.join(outputs_dir, "model_results.csv"), index=False, encoding="utf-8")
    print("Results saved to 'model_results.csv'.")
```
