# Supplementary material for: Tracing socioeconomic change and emerging elites in 4th–3rd century BCE: Metalwork from the Amber Road Corridor
Source: PLoS One. 2026 Sep 2;21(9):e0352885. doi: 10.1371/journal.pone.0352885 (PMC13537607; doi:10.1371/journal.pone.0352885)
Supplement: Supplementary file 2 [file pone.0352885.s001.docx]

Tracing Socioeconomic Change and Emerging Elites in 4th–3rd Century BCE: Metalwork from the Amber Road Corridor

Supplementary Information 2: PCA and K-means cluster analyses

Table of contents

[Elemental correlations	2](#_Toc216795314)

[Summary of PCA observations	3](#_Toc216795315)

[All cemeteries in the 4^th^ and the 3^rd^ century BCE from Bohemia and Moravia	6](#_Toc216795316)

[Bohemia – 4^th^ - 3^rd^ century BCE – cemeteries	12](#_Toc216795317)

[Moravia – 4^th^ - 3^rd^ century BCE – cemeteries	17](#_Toc216795318)

[Bohemia and Moravia cemeteries – 4^th^ century BCE	21](#_Toc216795319)

[Bohemia and Moravia cemeteries – 3^rd^ century BCE	26](#_Toc216795320)

[Moravia – 3^rd^ century BCE– cemeteries + agglomeration	32](#_Toc216795321)

[Moravia – 3^rd^ – 2^nd^ century BCE – agglomeration + coins	37](#_Toc216795322)

## Elemental correlations

Correlation matrices (Tab. S1-S4) show the trace elements that are characteristic of copper alloys in the La Tène period within the study area. These correlations appear more or less significantly throughout PCA runs performed for different combinations of assemblages, expressing individual chronological periods, or sites. From the correlation matrices alone it appears that certain non-specificity of the alloys typical for earlier periods is pushed in favour of fewer copper types that are expressed in the correlation matrices by higher specificity of the elements that correlate and their fewer numbers. It is visible especially for the 3^rd^ century BCE in Moravia (Tab. S4).

| Variable | Correlations (cemeteries) Marked correlations are significant at **p < .05000** N=326 (Casewise deletion of missing data) | | | | | | | |
| --- | --- | --- | --- | --- | --- | --- | --- | --- |
|  | **Fe** | **Co** | **Ni** | **Zn** | **As** | **Ag** | **Sb** | **Bi** |
| **Fe** | 1 | 0.0885 | -0.0462 | -0.0049 | 0.006 | -0.1186 | -0.0412 | -0.0797 |
| *p* |  | *p=.111* | *p=.406* | *p=.930* | *p=.915* | *p=.032* | *p=.459* | *p=.151* |
| **Co** | 0.0885 | 1 | 0.3734 | 0.0836 | 0.2864 | 0.1102 | 0.3275 | 0.0916 |
| *p* | *p=.111* |  | *p=.000* | *p=.132* | *p=.000* | *p=.047* | *p=.000* | *p=.099* |
| **Ni** | -0.0462 | 0.3734 | 1 | -0.0105 | 0.5595 | 0.1914 | 0.3631 | 0.0498 |
| *p* | *p=.406* | *p=.000* |  | *p=.851* | *p=0.00* | *p=.001* | *p=.000* | *p=.370* |
| **Zn** | -0.0049 | 0.0836 | -0.0105 | 1 | -0.0499 | -0.0654 | -0.0161 | -0.0169 |
| *p* | *p=.930* | *p=.132* | *p=.851* |  | *p=.369* | *p=.239* | *p=.772* | *p=.762* |
| **As** | 0.006 | 0.2864 | 0.5595 | -0.0499 | 1 | 0.2778 | 0.4233 | 0.1027 |
| *p* | *p=.915* | *p=.000* | *p=0.00* | *p=.369* |  | *p=.000* | *p=.000* | *p=.064* |
| **Ag** | -0.1186 | 0.1102 | 0.1914 | -0.0654 | 0.2778 | 1 | 0.303 | 0.1264 |
| *p* | *p=.032* | *p=.047* | *p=.001* | *p=.239* | *p=.000* |  | *p=.000* | *p=.022* |
| **Sb** | -0.0412 | 0.3275 | 0.3631 | -0.0161 | 0.4233 | 0.303 | 1 | 0.126 |
| *p* | *p=.459* | *p=.000* | *p=.000* | *p=.772* | *p=.000* | *p=.000* |  | *p=.023* |
| **Bi** | -0.0797 | 0.0916 | 0.0498 | -0.0169 | 0.1027 | 0.1264 | 0.126 | 1 |
| *p* | *p=.151* | *p=.099* | *p=.370* | *p=.762* | *p=.064* | *p=.022* | *p=.023* |  |

*Tab. S1. Correlation analysis and p-values of the samples from all the cemeteries in the 4^th^ and the 3^rd^ centuries BCE in Bohemia and Moravia.*

| Variable | Correlations (4th_BohMor) Marked correlations are significant at **p < .05000** N=219 (Casewise deletion of missing data) | | | | | | | | | |
| --- | --- | --- | --- | --- | --- | --- | --- | --- | --- | --- |
|  | Means | Std.Dev. | **Fe** | **Co** | **Ni** | **Zn** | **As** | **Ag** | **Sb** | **Bi** |
| **Fe** | 0.4189 | 0.6998 | 1.0000 | 0.0753 | -0.0496 | -0.0316 | 0.0063 | -0.1240 | -0.0595 | -0.1017 |
| **Co** | 0.0153 | 0.0153 | 0.0753 | 1.0000 | 0.4618 | -0.0222 | 0.3874 | 0.2608 | 0.3039 | 0.1250 |
| **Ni** | 0.1101 | 0.1910 | -0.0496 | 0.4618 | 1.0000 | -0.0058 | 0.6098 | 0.2748 | 0.3684 | 0.0269 |
| **Zn** | 0.1597 | 0.8927 | -0.0316 | -0.0222 | -0.0058 | 1.0000 | -0.0677 | -0.0648 | -0.0019 | -0.0088 |
| **As** | 0.1726 | 0.1800 | 0.0063 | 0.3874 | 0.6098 | -0.0677 | 1.0000 | 0.2966 | 0.4510 | 0.0954 |
| **Ag** | 0.0248 | 0.0170 | -0.1240 | 0.2608 | 0.2748 | -0.0648 | 0.2966 | 1.0000 | 0.3894 | 0.1346 |
| **Sb** | 0.1167 | 0.1303 | -0.0595 | 0.3039 | 0.3684 | -0.0019 | 0.4510 | 0.3894 | 1.0000 | 0.1102 |
| **Bi** | 0.0114 | 0.0255 | -0.1017 | 0.1250 | 0.0269 | -0.0088 | 0.0954 | 0.1346 | 0.1102 | 1.0000 |

*Tab. S2. Correlation analysis of the samples from all the cemeteries in the 4^th^ century BCE in Bohemia and Moravia.*

| Variable | Correlations (3rd_BohMor) Marked correlations are significant at **p < .05000** N=107 (Casewise deletion of missing data) | | | | | | | | | |
| --- | --- | --- | --- | --- | --- | --- | --- | --- | --- | --- |
|  | Means | Std.Dev. | **Fe** | **Co** | **Ni** | **Zn** | **As** | **Ag** | **Sb** | **Bi** |
| **Fe** | 0.3963 | 0.5134 | 1.0000 | 0.1273 | -0.0560 | 0.0724 | -0.0047 | -0.0992 | 0.0283 | 0.0758 |
| **Co** | 0.0154 | 0.0213 | 0.1273 | 1.0000 | 0.3666 | 0.2497 | 0.1049 | -0.1301 | 0.4251 | 0.0283 |
| **Ni** | 0.0719 | 0.0561 | -0.0560 | 0.3666 | 1.0000 | -0.0586 | -0.2573 | -0.0779 | 0.4471 | 0.4040 |
| **Zn** | 0.1472 | 0.8583 | 0.0724 | 0.2497 | -0.0586 | 1.0000 | 0.0202 | -0.0675 | -0.0594 | -0.0844 |
| **As** | 0.1487 | 0.0788 | -0.0047 | 0.1049 | -0.2573 | 0.0202 | 1.0000 | 0.3881 | 0.2849 | 0.1519 |
| **Ag** | 0.0324 | 0.0148 | -0.0992 | -0.1301 | -0.0779 | -0.0675 | 0.3881 | 1.0000 | 0.0776 | 0.2742 |
| **Sb** | 0.1108 | 0.0937 | 0.0283 | 0.4251 | 0.4471 | -0.0594 | 0.2849 | 0.0776 | 1.0000 | 0.2950 |

*Tab. S3. Correlation analysis of the samples from all the cemeteries in the 3^rd^ century BCE in Bohemia and Moravia.*

| Variable | Correlations (3rd_Mor) Marked correlations are significant at **p < .05000** N=74 (Casewise deletion of missing data) | | | | | | | | | |
| --- | --- | --- | --- | --- | --- | --- | --- | --- | --- | --- |
|  | Means | Std.Dev. | **Fe** | **Ni** | **Co** | **Zn** | **As** | **Ag** | **Bi** | **Sb** |
| **Fe** | 0.4028 | 0.5177 | 1.0000 | -0.0826 | 0.0550 | 0.0871 | -0.0523 | -0.0968 | -0.0170 | -0.0134 |
| **Ni** | 0.0751 | 0.0566 | -0.0826 | 1.0000 | 0.1223 | -0.0796 | -0.4377 | -0.1329 | 0.4667 | 0.1549 |
| **Co** | 0.0161 | 0.0193 | 0.0550 | 0.1223 | 1.0000 | 0.3355 | -0.0020 | -0.0357 | -0.0451 | -0.1456 |
| **Zn** | 0.1825 | 1.0247 | 0.0871 | -0.0796 | 0.3355 | 1.0000 | 0.0221 | -0.0802 | -0.1296 | -0.1076 |
| **As** | 0.1530 | 0.0850 | -0.0523 | -0.4377 | -0.0020 | 0.0221 | 1.0000 | 0.4740 | 0.1270 | 0.2685 |
| **Ag** | 0.0336 | 0.0138 | -0.0968 | -0.1329 | -0.0357 | -0.0802 | 0.4740 | 1.0000 | 0.2609 | 0.1869 |
| **Bi** | 0.0107 | 0.0082 | -0.0170 | 0.4667 | -0.0451 | -0.1296 | 0.1270 | 0.2609 | 1.0000 | 0.4194 |
| **Sb** | 0.1156 | 0.0647 | -0.0134 | 0.1549 | -0.1456 | -0.1076 | 0.2685 | 0.1869 | 0.4194 | 1.0000 |

*Tab. S4. Correlation analysis of the samples from all the cemeteries in the 3^rd^ century BCE in Moravia.*

## Summary of PCA observations

**Round 1: Pb Isotopes + Trace Elements**

Recurrent Patterns

- Sb–As–Ag groupings are consistent across contexts, especially in Moravian cemeteries.
- Zn and Bi occasionally load with PC2 in 3rd-century contexts, potentially linked to low-melting additions or recycling.
- Ni–Co co-load moderately in 3rd-century datasets, implying technological variability (possibly technology, or mineral provenance, or slag interaction).

| **Context** | **Century** | **Region** | **Observed Pattern** |
| --- | --- | --- | --- |
| 4th c. Bohemia | 4th BCE | Bohemia | Scattered Pb signatures, wide range of trace element expressions |
| 4th c. Moravia | 4th BCE | Moravia | Similar scatter, with some distinct high Sb/As samples |
| 3rd c. Moravia | 3rd BCE | Moravia | Tighter Pb clusters, more compositional structure in PC2 |
| 3rd c. Moravia + Němčice | 3rd BCE | Moravia | Increased clustering, clearer patterning of trace elements (Sb–As–Zn) |

*Interpretation*:
Round 1 shows that 4th-century cemetery metals were derived from multiple sources and likely reflect non-specialised, localised production.
In the 3rd century BCE, Moravian cemeteries show increasing clustering — especially in trace element chemistry — indicating a shift toward more consistent alloying practices or shared material pools.

**Round 2: Trace Elements Only**

Key Components

- PC1: Reflects Sb–As–Ag variation — linked to ore types (fahlore) or alloy choices.
- PC2: Often highlights Zn–Co–Ni differences — the same observation as PC1

Recurrent Patterns

- 4th-century samples show high spread across both PCs, lacking clear groupings.
- 3rd-century samples, particularly in Moravia, begin to form tighter compositional clusters, with repeatable patterns of Sb-rich or Zn-rich alloys.

| **Context** | **Century** | **Region** | **Observed Pattern** |
| --- | --- | --- | --- |
| 4th c. Bohemia | 4th BCE | Bohemia | Wide scatter, no dominant elemental trend |
| 4th c. Moravia | 4th BCE | Moravia | Dispersed signals, |
| 3rd c. Moravia | 3rd BCE | Moravia | Clustering emerges: |
| 3rd c. Moravia + Němčice | 3rd BCE | Moravia | Clearer clusters, |

*Interpretation*:
Round 2 confirms the lack of standardised alloying in the 4th century BCE. Instead, we see a variety of recipes and metal sources, likely shaped by local decisions.
In the 3rd century, cemetery metals become more compositionally cohesive, pointing to emerging standardisation — even before full agglomeration production centres dominate.

*4th Century BCE Production - Not homogeneous — but decentralised and uncoordinated.*

- The spread of 4th-century samples in both PCA rounds supports a model of community-based, non-specialised production.
- Instead of being “local and stable,” the Pb isotope scatter implies variable ore inputs, possibly scavenged or traded in small quantities.
- The lack of clustering in trace elements suggests inconsistent alloying practices — not yet professionalised or standardised.

This is not technological underdevelopment — but a sign of:

- Household-level or itinerant craft,
- Limited oversight or redistribution systems,
- Broad access to a variety of ores, without tight metallurgical control.

*Contrast: 3rd Century BCE Production*

In the 3rd century BCE, by contrast:

- PCA shows tighter clustering, especially in Moravian cemeteries and coinage.
- Clusters reflect more structured choices: e.g., increased presence of Sb–As–Ag-Ni alloys and regional patterning (e.g., Zn, Ni, Ag).
- While sources remain diverse, their compositional signatures become more predictable, likely reflecting:
  - Consolidation of production in workshops or agglomerations,
  - More selective recycling or alloying,
  - Possibly elite or institutional control of bronze circulation.

| **Aspect** | **4th Century BCE** | | **3rd Century BCE** | |  |
| --- | --- | --- | --- | --- | --- |
| Organisation | Dispersed, local | | Centralising, regionalised | |  |
| Craft scale | Household, mobile | | Workshop, specialised | |  |
| Alloy consistency | Low, non-standard | | Moderate–high, standardising | |  |
| Pb isotope pattern | Dispersed, uncoordinated sourcing | | More structured — suggests mixing, reuse | |  |
| Trace element pattern | Unstructured Sb–As–Zn | | Clustered profiles tied to sites/regions | |  |
| **Feature** | | **4th Century BCE** | | **3rd Century BCE** | |
| Production mode | | Dispersed, non-specialised | | Increasingly centralised or workshop-based | |
| Isotopic signatures | | Scattered, multi-source | | Tighter clusters, suggesting ore selection/recycling | |
| Trace element diversity | | High but unstructured | | High, but more patterned by site and region | |
| Alloying indicators | | Inconsistent patterns, many combinations: Sb-Ag, Ni-As, Zn-Bi, As-Co, Ni-Sb-Ag, Ni-As-Co | | Clear clusters: Ni, Co, Bi, As, Ag e.g., Sb–Ag (Boh), Co-Ni, Ni-As (LT B2b sluster) in cemeteries, Zn–Bi, Sb-Ag-Zn, Co-Fe in agglomerations | |
| Regional coherence | | Low: Bohemia and Moravia both scattered | | Moderate: Moravian sites show more structure | |
| Technological character | | Low specialisation, opportunistic mixtures | | Emerging technical profiles, possible recipes | |

**Summary of Most Recurrent Element Combinations**

| **Element Group** | **Interpretation** |
| --- | --- |
| **Pb isotopes** | Provenance indicator (Round 1 PC1) |
| **Sb–As–Ag** | Alloy type, fahlore ores, or recycled material |
| **Zn–Bi** | Possibly linked to lower-temperature casting, secondary additions, Pb ore traces |
| **Ni–Co** | Reflective of smelting context, mineral provenance, impurity |

**Overall Conclusion**

The 4th century BCE samples are more dispersed, especially in Pb isotopic space (PC1), indicating multiple ore sources, and trace element space (Round 2 PCA), with moderate but clear divergence in Sb, As, Co, Zn — even among objects from the same site or region. No clear compositional signatures or clustering was observed. Rather than reflecting homogeneous, centralised production, the 4th century BCE material pattern more plausibly points to non-specialised, decentralised production units, likely small-scale and locally managed, diverse sourcing strategies — including multiple primary ore sources (reflected in isotopic spread) and possibly varying access to alloying recipes or materials, and a lack of standardisation. The data reflect rather a fluid, flexible, and low-hierarchy system of metal use. It reflects a landscape of experimentation and local independence, with no strong control over production inputs and clear centres of alloying authority. In other words, the 4th-century pattern aligns more with technological plurality than with coordinated networks.

While Bohemian cemeteries maintain this relatively traditional metallurgical signature, Moravian cemeteries in the 3rd century begin to reflect the metallic diversity and alloying innovations seen later in agglomeration sites. The apparent “order” of the 3rd century emerges *not from the absence of variation*, but from the systematic structuring of diversity — through specialisation, recycling, and technological innovation. The 3rd century BCE, especially in Moravia, marks the onset of compositional discipline, observable through both Pb isotope convergence and trace element patterning. These trends precede the full emergence of agglomerations and suggest that the cemetery populations were agents of innovation, active transmitters of technological transformation, not just its passive consumers.

## All cemeteries in the 4^th^ and the 3^rd^ century BCE from Bohemia and Moravia

**Round 1: Pb Isotopes + Trace Elements**

Variables:
*²⁰⁶Pb/²⁰⁴Pb, ²⁰⁷Pb/²⁰⁴Pb, ²⁰⁸Pb/²⁰⁴Pb, logFe, logCo, logNi, logZn, logAg, logAs, logSb, logBi*

Explained Variance:

- **PC1: 28.1%** - dominated by ²⁰⁶Pb/²⁰⁴Pb, ²⁰⁷Pb/²⁰⁴Pb, ²⁰⁸Pb/²⁰⁴Pb → reflects ore source differences
- **PC2: 17.0%** - shaped by logSb, logAg, logAs → indicates mineral resources (fahlores)

|  | **PC1** | **PC2** |
| --- | --- | --- |
| **206Pb/204Pb** | -0.471 | 0.289 |
| **207Pb/204Pb** | -0.48 | 0.248 |
| **208Pb/204Pb** | -0.501 | 0.303 |
| **logFe** | 0.03 | -0.038 |
| **logCo** | -0.144 | -0.366 |
| **logNi** | -0.272 | -0.391 |
| **logZn** | 0.047 | 0.04 |
| **logAg** | -0.298 | -0.315 |
| **logAs** | -0.031 | -0.43 |
| **logSb** | -0.314 | -0.434 |
| **logBi** | 0.098 | 0.036 |

*PCA loadings for the Round 1*

Fig. 1

Fig. 2

Fig. 3

Fig. 4

**PCA Round 2: Trace Elements Only**

Variables:
*logFe, logCo, logNi, logZn, logAg, logAs, logSb*

- **PC1: 30.9%** - indicates mineral resources
- **PC2: 16.3%** - indicates mineral resources

|  | **PC1** | **PC2** |
| --- | --- | --- |
| **logFe** | 0.008 | -0.588 |
| **logCo** | -0.374 | -0.319 |
| **logNi** | -0.489 | -0.172 |
| **logZn** | 0.056 | -0.427 |
| **logAg** | -0.455 | 0.392 |
| **logAs** | -0.32 | -0.377 |
| **logSb** | -0.556 | 0.211 |

*PCA loadings for the Round 2*

Fig. 5

Fig. 6

Fig. 7

Fig. 8

**K-means clustering**

Interpretation of Cluster Patterns

**PCA Round 1 (Pb + Trace Elements, 3 Clusters)**

- Cluster 0 - Higher PC1 scores, dominated by lead isotope ratios → points to distinct ore provenance. Tighter grouping suggests controlled material. Dominates in LT B1- LT B2a, possibly linked to early lead-sources and more homogeneous alloy recipes.
- Cluster 1 - Higher PC2 scores, strongly influenced by logSb and logAs. Indicates mineral resources (fahlores). Increases significantly by LT B2b–C1 and C1.
- Cluster 2 - Intermediate PC1 and PC2 values; diverse trace element profiles. Likely blended or regionally variable input, pointing to distributed production networks or trade exchange. Remains moderate throughout, possibly representing transitional, mixed or non-distinctive sources.

**PCA Round 2 (Trace Elements Only, 2 Clusters)**

- Not a good result

Fig. 9

## Bohemia – 4^th^ - 3^rd^ century BCE – cemeteries

**Round 1: Pb Isotopes + Trace Elements**

Variables used:
*²⁰⁶Pb/²⁰⁴Pb, ²⁰⁷Pb/²⁰⁴Pb, ²⁰⁸Pb/²⁰⁴Pb, logFe, logCo, logNi, logZn, logAg, logAs, logSb, logBi*

Explained Variance:

- **PC1 = 30.0%** — strongly influenced by Pb isotopes (²⁰⁶Pb/²⁰⁴Pb, ²⁰⁷Pb/²⁰⁴Pb, ²⁰⁸Pb/²⁰⁴Pb) → reflects ore provenance differences.
- **PC2 = 17.9%** — driven by logSb, logAs, and logAg → indicates mineral resources

**Pattern Observations:**

- Later La Tène phases (LT B2b–C1, LT C1) align more with PC2, consistent with complex alloying or workshop traditions.
- Clustering by century confirms a temporal trend in technology or provenance.
- Chronological trends.

|  | **PC1** | **PC2** |
| --- | --- | --- |
| **206Pb/204Pb** | -0.431 | -0.29 |
| **207Pb/204Pb** | -0.431 | -0.26 |
| **208Pb/204Pb** | -0.46 | -0.311 |
| **logFe** | 0.018 | -0.119 |
| **logCo** | -0.132 | 0.445 |
| **logNi** | -0.343 | 0.389 |
| **logZn** | 0.069 | 0.078 |
| **logAg** | -0.333 | 0.209 |
| **logAs** | -0.099 | 0.404 |
| **logSb** | -0.368 | 0.326 |
| **logBi** | 0.146 | 0.262 |

*PCA loadings for the Round 1*

Fig. 10

Fig. 11

Fig. 12

**PCA Round 2: Trace Elements Only**

Variables used:
*logFe, logCo, logNi, logZn, logAg, logAs, logSb, logBi*

Explained Variance:

- **PC1 = 30.4% —** defined by logSb, logAg, logNi, and then logCo, logAs → mineral resources and technology.
- **PC2 = 20.9%** —influenced by logBi and logZn,

Pattern Observations:

- Sites with high PC2 scores often align with potentially polymetallic ores and Pb sources (but not aligning entirely with late phases).
- Low PC1 + low PC2 sites show cleaner alloy signals, or non-distinctive mineral signal. Maybe reuse, mixing.
- Chronological trends.

These results show that in both rounds, the first two principal components explain around half of the total variation—suggesting strong underlying patterns in the isotopic and elemental composition data.

|  | **PC1** | **PC2** |
| --- | --- | --- |
| **logFe** | -0.061 | -0.121 |
| **logCo** | 0.342 | 0.3 |
| **logNi** | 0.533 | 0.059 |
| **logZn** | -0.112 | 0.559 |
| **logAg** | 0.449 | -0.189 |
| **logAs** | 0.275 | 0.371 |
| **logSb** | 0.547 | -0.088 |
| **logBi** | -0.076 | 0.631 |

*PCA loadings for the Round 2*

Fig. 13

Fig. 14

Fig. 15

**K-means clustering**

Interpretation of Clusters

**PCA Round 1 (2 Clusters – Pb Isotopes + Trace Elements)**

- Cluster 0: especially prevalent in 4th-century BCE.
- Cluster 1: Characterised by greater isotopic and elemental variability, later phases only in this cluster.

**PCA Round 2 (3 Clusters – Trace Elements Only)**

Not a good result.

Fig. 16

## Moravia – 4^th^ - 3^rd^ century BCE – cemeteries

**PCA Round 1: Pb Isotopes + Trace Elements**

*Variables used:*

- ²⁰⁶Pb/²⁰⁴Pb, ²⁰⁷Pb/²⁰⁴Pb, ²⁰⁸Pb/²⁰⁴Pb
- logFe, logCo, logNi, logZn, logAg, logAs, logSb, logBi

*Explained Variance:*

- PC1: 27.5% dominated by Pb isotopes + trace metal mixture)
- PC2: 14.6% driven more by trace elements: Co, Ni, Ag, As, Sb > mineral types

*Clustering Patterns:*

- Clear grouping by SITE in some quadrants, sites tend to cluster differently.
- DATING ITEM shows structuring; earlier vs later phases show different chemical signatures.

|  | **PC1** | **PC2** |
| --- | --- | --- |
| **206Pb/204Pb** | -0.508 | 0.198 |
| **207Pb/204Pb** | -0.526 | 0.12 |
| **208Pb/204Pb** | -0.538 | 0.167 |
| **logFe** | 0.078 | -0.085 |
| **logCo** | -0.146 | -0.365 |
| **logNi** | -0.152 | -0.353 |
| **logZn** | 0.027 | 0.202 |
| **logAg** | -0.23 | -0.341 |
| **logAs** | 0.117 | -0.45 |
| **logSb** | -0.213 | -0.539 |
| **logBi** | -0.116 | -0.061 |

*PCA loadings for the Round 1*

Fig. 17

Fig. 18

**PCA Round 2: Trace Elements**

*Variables used:*

- logFe, logCo, logNi, logZn, logAg, logAs, logSb

*Explained Variance:*

- PC1: 25.1% > balanced contributions from logCo, logNi, logAg, logSb
- PC2: 21.3% > stronger influence from logAs, logNi, logFe

*Clustering Patterns:*

- SITE clustering is maintained even without Pb isotopes, suggesting trace element composition alone can differentiate technological or regional traditions.
- Variation by DATING ITEM remains, though less pronounced, implying trace element fingerprints may capture production techniques during time and capture the chronological change.

|  | **PC1** | **PC2** |
| --- | --- | --- |
| **logFe** | 0.154 | -0.482 |
| **logCo** | -0.376 | -0.173 |
| **logNi** | -0.254 | -0.495 |
| **logZn** | 0.1 | 0.292 |
| **logAg** | -0.567 | 0.34 |
| **logAs** | -0.143 | -0.538 |
| **logSb** | -0.647 | 0.045 |

*PCA loadings for the Round 2*

Fig. 19

Fig. 20

**K-means clustering**

Interpretation of Clusters

**By Site**

- Holubice, Maloměřice, Mistřín, and Nechvalín show a strong concentration in Cluster 0 for both PCA rounds.
- Sites like Tikovice and Pustiměřské Prusy have more balanced membership across clusters, suggesting technological or compositional diversity.
- The site Němčice is small in sample size but shows membership in both clusters, indicating variability.

**By Dating Item**

- LT B2b and LT B2a phases dominate both clusters, but Cluster 1 is slightly more prevalent in LT B1 and LT B2b.

Later phases (LT C1) are almost exclusively in Cluster 0, hinting at either more standardised material inputs or/and organised practices.

Fig. 21

Fig. 22

## Bohemia and Moravia cemeteries – 4^th^ century BCE

**PCA Round 1 (Pb Isotopes + Trace Elements)**

Variables:
*²⁰⁶Pb/²⁰⁴Pb, ²⁰⁷Pb/²⁰⁴Pb, ²⁰⁸Pb/²⁰⁴Pb, logFe, logCo, logNi, logZn, logAg, logAs, logSb, logBi*

Explained Variance:

- PC1: 30.8% - is driven primarily by lead isotope ratios (²⁰⁶Pb/²⁰⁴Pb, ²⁰⁷Pb/²⁰⁴Pb, ²⁰⁸Pb/²⁰⁴Pb), also logNi, logAg, logSb (negatively)- suggesting that it reflects differences in ore source or metallurgical provenance
- PC2: 17.7% - most influenced by LogNi, logCo, logSb, logAs, and logAg (positively) → indicates mineral resources (fahlores).

The clusters differentiate - indicating that trace element composition (especially Sb, As, Ag, Co, Ni) is a key driver of metallurgical grouping. Bi always cluster with Zn.

Spatial and chronological biplots show partial separation by region and period, supporting the interpretation of evolving production and alloying systems.

|  | **PC1** | **PC2** |
| --- | --- | --- |
| **206Pb/204Pb** | -0.42 | -0.332 |
| **207Pb/204Pb** | -0.439 | -0.298 |
| **208Pb/204Pb** | -0.453 | -0.348 |
| **logFe** | 0.029 | 0.041 |
| **logCo** | -0.164 | 0.389 |
| **logNi** | -0.309 | 0.366 |
| **logZn** | 0.064 | -0.016 |
| **logAg** | -0.384 | 0.243 |
| **logAs** | -0.1 | 0.446 |
| **logSb** | -0.349 | 0.357 |
| **logBi** | 0.136 | 0.084 |

*PCA loadings for the Round 1*

Fig. 23

Fig. 24

Fig. 25

**PCA Round 2 (Trace Elements Only)**

Variables:
*logFe, logCo, logNi, logZn, logAg, logAs, logSb, logBi*

Explained Variance: these values suggest a well structured PCA space with trace elements showing slightly stronger dimensional separation in Round 2.

- PC1: 35.2% - correlates with logAs, logSb, logCo, and logNi (negatively), reflecting mineral resources and regionality.
- PC2: 15.5% - reflects high influence from logFe, logCo, logZn (negatively), reflecting mineral resources and regionality.

The clustering cleanly separates samples with higher antimony/arsenic from those with cleaner alloy signatures. This suggests the presence of at least two technological traditions or resource access patterns.

|  | **PC1** | **PC2** |
| --- | --- | --- |
| **logFe** | 0.007 | -0.609 |
| **logCo** | -0.366 | -0.363 |
| **logNi** | -0.487 | -0.083 |
| **logZn** | 0.073 | -0.635 |
| **logAg** | -0.48 | 0.21 |
| **logAs** | -0.341 | -0.151 |
| **logSb** | -0.526 | 0.141 |

*PCA loadings for the Round 2*

Fig. 26

Fig. 27

Fig. 28

**K-means clustering**

Optimal number of clusters for both PCA rounds is 2, based on silhouette score analysis.

Interpretation of Cluster Patterns

**PCA Round 1 (Pb + Trace Elements)**

- Cluster 0: Samples with distinct Pb isotopic signatures (high ²⁰⁶Pb/²⁰⁴Pb, etc.), suggesting consistent Pb ore provenance.
- Cluster 1: Greater variability in Co, Ni, Fe, Sb, As, and Ag → likely reflecting mineral resources.

**PCA Round 2 (Trace Elements Only) > not good patterning results.**

- However, comparison of the two rounds reveals that some samples are consistently grouped, suggesting robust metallurgical identity.
- Others shift between clusters, highlighting differences in isotopic vs compositional clustering, useful for identifying mixed or hybrid practices.

Fig. 29

## Bohemia and Moravia cemeteries – 3^rd^ century BCE

**PCA Round 1: Pb Isotopes + Trace Elements**

Variables used:
*²⁰⁶Pb/²⁰⁴Pb, ²⁰⁷Pb/²⁰⁴Pb, ²⁰⁸Pb/²⁰⁴Pb, logFe, logCo, logNi, logZn, logAg, logAs, logSb, logBi*

- **PC1 (27.2%):** Primarily influenced by ²⁰⁶Pb/²⁰⁴Pb, ²⁰⁷Pb/²⁰⁴Pb, ²⁰⁸Pb/²⁰⁴Pb, reflecting differences in ore source/provenance.
- **PC2 (15.4%):** Dominated by logSb, logAs, logCo, logNi, logFe, (positively) and logAg (negatively)→ likely linked to provenance, or recycling practices or technological differentiation.

Key Observations:

- Bohemian and Moravian samples partially separate in PCA space, suggesting regional alloying preferences.
- Later-phase samples (since B2b-C1) cluster in the centre suggesting homogeneity, while LT B2b phase is dispersed, suggesting material and technological preferences.

|  | **PC1** | **PC2** |
| --- | --- | --- |
| **206Pb/204Pb** | -0.512 | 0.013 |
| **207Pb/204Pb** | -0.508 | 0.143 |
| **208Pb/204Pb** | -0.546 | 0.094 |
| **logFe** | 0.05 | 0.274 |
| **logCo** | -0.13 | 0.327 |
| **logNi** | -0.09 | 0.5 |
| **logZn** | -0.001 | 0.283 |
| **logAg** | -0.199 | -0.4 |
| **logAs** | 0.174 | 0.42 |
| **logSb** | -0.268 | -0.05 |
| **logBi** | 0.112 | 0.348 |

*PCA loadings for the Round 1*

Fig. 30

Fig. 31

Fig. 32

**PCA Round 2: Trace Elements Only**

Variables used:
*logFe, logCo, logNi, logZn, logAg, logAs, logSb, logBi*

- **PC1 (21.7%):** Shaped by logAs, logBi, logNi, logZn (positively) and logSb, logAg (negatively) – likely indicating base alloy composition – material preference.
- **PC2 (17.9%):** Dominated by logCo, logNi and logSb, reinforcing the importance of workshop-level suggesting material preference.

**Key Observations**:

- Clearer separation by region and century, suggesting temporal and regional evolution of alloying methods.
- Not so much differences in phases. Same since LT B2b. But there is a cluster with mostly LT B2b data, consistent with 4^th^ centuryBCE– traditional older production?

Overall results indicate that both PCAs effectively reduce dimensionality while capturing key differences in alloy composition and provenance.

|  | **PC1** | **PC2** |
| --- | --- | --- |
| **logFe** | 0.3 | -0.093 |
| **logCo** | 0.135 | 0.665 |
| **logNi** | 0.362 | 0.437 |
| **logZn** | 0.299 | -0.133 |
| **logAg** | -0.474 | -0.002 |
| **logAs** | 0.482 | -0.017 |
| **logSb** | -0.275 | 0.583 |
| **logBi** | 0.372 | -0.035 |

*PCA loadings for the Round 2*

Fig. 33

Fig. 33

Fig. 34

**K-means clustering**

**PCA Round 1** (3 Clusters: Pb + Trace Elements)

- Cluster 0: Common at Bohemian sites, associated with more homogeneous isotopic signatures → may represent locally sourced, standard alloy recipes.
- Cluster 1: Increases in 3rd centuryBCEcontexts, notably at Moravian sites, and includes more diverse compositions.
- Cluster 2: Appears at cross-period sites (B2b, C1) and is often linked to transitional or traditional alloy profiles.

**PCA Round 2** (2 Clusters: Trace Elements Only)

- Cluster 0: diverse contexts in both regions.
- Cluster 1: Found mainly in Moravian and LT B2b and moderately LT C1 contexts, continuity from the 4^th^ centuryBCE– tradition?

**Cross-Round Summary**

- Some samples consistently group together, highlighting a stable metallurgical identity across both PCA methods.
- Shifts in cluster membership often track chronological and regional transitions.

Fig. 35

Fig. 36

## Moravia – 3^rd^ century BCE– cemeteries + agglomeration

**PCA Round 1 (Pb Isotopes + Trace Elements)**

Variables used:
*²⁰⁶Pb/²⁰⁴Pb, ²⁰⁷Pb/²⁰⁴Pb, ²⁰⁸Pb/²⁰⁴Pb, logFe, logCo, logNi, logZn, logAg, logAs, logSb, logBi*

- **PC1 (28.3%) is driven strongly by:**
  - ²⁰⁶Pb/²⁰⁴Pb, ²⁰⁷Pb/²⁰⁴Pb, ²⁰⁸Pb/²⁰⁴Pb (negative) → reflects Pb isotopic provenance
  - Moderate contributions from logBi (negative), logAs (positive), suggesting compositional links to copper minerals.
- **PC2 (13.0%) is shaped mainly by:**
  - logNi, logAs, (positive), possibly related to mineral types or specific workshops.

|  | **PC1** | **PC2** |
| --- | --- | --- |
| **206Pb/204Pb** | -0.523 | 0.148 |
| **207Pb/204Pb** | -0.522 | 0.135 |
| **208Pb/204Pb** | -0.539 | 0.178 |
| **logFe** | 0.019 | 0.277 |
| **logCo** | -0.11 | 0.095 |
| **logNi** | 0.025 | 0.563 |
| **logZn** | -0.144 | -0.221 |
| **logAg** | -0.112 | -0.376 |
| **logAs** | 0.237 | 0.568 |
| **logSb** | 0.032 | -0.034 |
| **logBi** | -0.244 | -0.11 |

*PCA loadings for the Round 1*

Fig. 37

Fig. 38

**PCA Round 2 (Trace Elements Only)**

Variables used:
*logFe, logCo, logNi, logZn, logAg, logAs, logSb, logBi*

- **PC1 (21.7%):**
  - Balanced influence from logFe, logAs, and logNi, potentially identifying copper sources
- **PC2 (18.7%):**
  - Dominated by equally by LogNi, logSb, and logAg, related to mineral types.

|  | **PC1** | **PC2** |
| --- | --- | --- |
| **logFe** | 0.3 | -0.33 |
| **logCo** | -0.035 | -0.062 |
| **logNi** | 0.499 | 0.466 |
| **logZn** | -0.26 | 0.273 |
| **logAg** | -0.366 | 0.444 |
| **logAs** | 0.677 | 0.169 |
| **logSb** | -0.028 | 0.608 |

*PCA loadings for the Round 2*

Fig. 39

Fig. 40

**K-means clustering**

Interpretation of Cluster Patterns

**🔹 PCA Round 1 (Pb Isotopes + Trace Elements)**

- Cluster 1: Characterised by high PC1 scores, primarily influenced by ²⁰⁶Pb/²⁰⁴Pb, ²⁰⁷Pb/²⁰⁴Pb, ²⁰⁸Pb/²⁰⁴Pb. Likely represents objects made from distinct lead ore sources. These samples tend to cluster tightly, suggesting consistency in provenance.
- Cluster 2: Intermediate PC1 and PC2 scores, with moderate variance. Shows some influence from trace elements like logBi , logZn, logFe, logCo, logAg (stronger), but not extreme in any dimension. This may represent blended sources or objects combining new and recycled metal. A mixed-use group, reflecting either alloying flexibility or redistribution from multiple sites (e.g., through trade or reuse).
- Cluster 0: Strongly shaped by logAs, logNi. Suggests objects with high levels of distinct mineral ore elements. This group is more dispersed, consistent maybe with less controlled inputs.

**🔸 PCA Round 2 (Trace Elements Only)**

- Clusters separate clearly along logAs and logNi, highlighting mineral types, or ore elements.
- Cluster 1: prevailing objects from the LT B2b phase, only limited objects from LT C1 phase.
- Cluster 0: all chronological phases represented. Youngest objects (LT C2) tend to cluster in contrast to LT C1.

Fig. 41

Fig. 42

## Moravia – 3^rd^ – 2^nd^ century BCE – agglomeration + coins

**PCA Round 1: Pb Isotopes + Trace Elements**

Variables used:
*²⁰⁶Pb/²⁰⁴Pb, ²⁰⁷Pb/²⁰⁴Pb, ²⁰⁸Pb/²⁰⁴Pb, logFe, logCo, logNi, logZn, logAg, logAs, logSb, logBi*

- **PC1:** 36.7% , negative correlation with Pb isotopes, moderate positive influence of logZn, logAg, logSb, and logBi. Foreign samples like Syracuse, Alexandria, Carthage group separately along PC1 → consistent with different metal sources.
- **PC2:** 14.6%, influenced by logNi, logAg, logAs, logSb. Mostly indikative of local production.

|  | **PC1** | **PC2** |
| --- | --- | --- |
| **206Pb/204Pb** | -0.467 | 0.041 |
| **207Pb/204Pb** | -0.421 | 0.052 |
| **208Pb/204Pb** | -0.433 | 0.052 |
| **logFe** | -0.112 | 0.229 |
| **logCo** | -0.338 | 0.015 |
| **logNi** | -0.015 | 0.403 |
| **logZn** | 0.295 | 0.024 |
| **logAg** | 0.238 | 0.359 |
| **logAs** | -0.128 | 0.62 |
| **logSb** | 0.237 | 0.5 |
| **logBi** | 0.272 | -0.117 |

*PCA loadings for the Round 1*

Fig. 43

Fig. 44

Fig. 45

**PCA Round 2: Trace Elements Only**

Variables used:
*logFe, logCo, logNi, logZn, logAg, logAs, logSb, logBi*

- **PC1:** 29.4%: negative influence of logSb, logAg, logZn, logBi, positive influence of logCo, and logAs
- **PC2:** 19.9%, positive influence of logNi, logAs, logSb, logAg, indicating local production.

Distinction between coins with controlled alloys and those enriched in trace elements. Foreign samples are again offset from the local cluster, affirming their distinct metallurgical histories.

|  | **PC1** | **PC2** |
| --- | --- | --- |
| **logFe** | 0.046 | 0.192 |
| **logCo** | 0.519 | 0.097 |
| **logNi** | 0.037 | 0.431 |
| **logZn** | -0.501 | -0.062 |
| **logAg** | -0.381 | 0.299 |
| **logAs** | 0.185 | 0.664 |
| **logSb** | -0.391 | 0.455 |
| **logBi** | -0.381 | -0.164 |

*PCA loadings for the Round 2*

Fig. 46

Fig. 47

Fig. 48

**K-means clustering**

Interpretation of Cluster Patterns

**PCA Round 1 (2 Clusters – Pb + Trace Elements)**

- Cluster 0: Dominated by local production, most samples show tightly grouped isotopic compositions, consistent with shared source or controlled alloying.
- Cluster 1: Includes foreign imports like Syracuse, Carthage, Alexandria. Exhibits distinct Pb isotope signatures and trace element variation → likely reflects different ore sources and technologies.

**PCA Round 2 (3 Clusters – Trace Elements Only)**

Bad result – 2 clusters is optimal

Fig. 49

### Site-level variability in ternary trace-element classification

To facilitate site-level interpretation of the ternary classification shown in Fig. 4, Supplementary Figs. 50–51 provide the same ternary system plotted separately for each site, with the overall distribution shown in grey and the respective site highlighted in colour.

**Fig. 50.** Site-level ternary plots of ore-indicator trace-element groups in **4th‑century BCE** copper-alloy artefacts (Bohemia and Moravia). The ternary coordinates are defined as **T = Σ(Co+Ni)** (chalcopyrite-associated indicators), **L = Σ(Ag+As+Sb)** (fahlore-associated indicators), and **R = Zn** (galena-associated indicator), expressed as wt.% (see Tab. S1–S4 for correlation matrices and element selection). Grey points show the distribution of all 4th‑century samples; coloured points highlight the respective site in each panel.

**Fig. 51.** Site-level ternary plots of ore-indicator trace-element groups in **3rd‑century BCE** copper-alloy artefacts (Bohemia and Moravia), including the Němčice nad Hanou agglomeration; a separate panel shows Mediterranean coins from Němčice for comparison. Ternary coordinates as in Fig. 50: **T = Σ(Co+Ni)**, **L = Σ(Ag+As+Sb)**, **R = Zn**. Grey points show the overall 3rd‑century distribution; coloured points highlight the respective site in each panel

**Fig. 52:** Site-level PCA plots of trace-element patterning in 4th-century BCE copper-alloy artefacts from Bohemia and Moravia. Grey points show the overall distribution of the 4th-century cemetery dataset; coloured points highlight the assemblage of the respective site in each panel. The plots correspond to the PCA dataset discussed in the main text for the 4th-century cemeteries (cf. Fig. 6).

**Fig. 53:** Site-level PCA plots of trace-element patterning in 3rd-century BCE copper-alloy artefacts from Bohemia and Moravia. Grey points show the overall distribution of the 3rd-century cemetery dataset; coloured points highlight the assemblage of the respective site in each panel. The plots correspond to the PCA dataset discussed in the main text for the 3rd-century cemeteries (cf. Fig. 7).
