# Supplementary material for: Tracing socioeconomic change and emerging elites in 4th–3rd century BCE: Metalwork from the Amber Road Corridor
Source: PLoS One. 2026 Sep 2;21(9):e0352885. doi: 10.1371/journal.pone.0352885 (PMC13537607; doi:10.1371/journal.pone.0352885)
Supplement: Supplementary file 3 [file pone.0352885.s002.docx]

## Tracing Socioeconomic Change and Emerging Elites in 4th–3rd Century BCE: Metalwork from the Amber Road Corridor

## Supplementary Information 3: Isotopic analysis

Content:

[Tracing Socioeconomic Change and Emerging Elites in 4th–3rd Century BCE: Metalwork from the Amber Road Corridor	1](#_Toc216787478)

[Supplementary Information 3: Isotopic analysis	1](#_Toc216787479)

[1. General Patterns in the Data	1](#_Toc216787480)

[1.1 Diachronic Trends: 4th–2nd Century BCE	1](#_Toc216787481)

[1.2 Correlation with Lead Content	3](#_Toc216787482)

[2. Lead isotope systematics in relation to trace element chemistry	3](#_Toc216787483)

[2.1 Diachronic shifts in isotopic–chemical relationships	4](#_Toc216787484)

[2.2 Correlation between isotope values and alloying elements	4](#_Toc216787485)

[2.2 Pb‑isotope distribution of trace‑element PCA clusters (3rd century BCE Moravia)	9](#_Toc216787486)

[3. Site-based variation in lead isotope systematics	9](#_Toc216787487)

[3.1 The 4th century BCE: broad dispersion and limited structure	10](#_Toc216787488)

[3.2 The 3rd century BCE: increasing consistency, persistent outliers	10](#_Toc216787489)

[4. The Němčice Agglomeration: Internal Isotopic Trends	15](#_Toc216787490)

[4.1 Phase-wise isotope variation and relationship to Cluster	15](#_Toc216787491)

[Table 1. Isotopic and geochemical parameters by phase (Němčice)	15](#_Toc216787492)

[5. Comparative biplots of artefact Pb isotopes against ore‑region datasets	17](#_Toc216787493)

[Summary of observations (by century)	18](#_Toc216787494)

[Limitations	18](#_Toc216787495)

[Figures:	18](#_Toc216787496)

## 1. General Patterns in the Data

### 1.1 Diachronic Trends: 4th–2nd Century BCE

In the **4th century BCE**, the artefacts show a broad scatter of lead isotope ratios (Fig. 1:A, C), with:

- ²⁰⁶Pb/²⁰⁴Pb ranging from ~18.14 to 18.83,
- ²⁰⁷Pb/²⁰⁴Pb between 15.55 and 15.68.

This variability in lead isotope systematics suggests a decentralised system of metal acquisition and production involving:

- multiple geological sources,
- frequent mixing or recycling,
- and local, unspecialised workshop practices.

By the **3rd century BCE**, and especially in the later part of this period, a clear cluster emerges (Fig. 1:B, D):

- ²⁰⁶Pb/²⁰⁴Pb between **18.4–18.55**,
- ²⁰⁷Pb/²⁰⁴Pb around **15.61–15.63**.

**Fig. 1.** Lead isotope (²⁰⁷Pb/²⁰⁴Pb vs. ²⁰⁶Pb/²⁰⁴Pb) and model age vs. Th/U (κ) systematics of studied artefacts. A–B: Lead isotope ratios for the 4th (A) and 3rd and 2nd (B) centuries BCE; C–D: Corresponding κ–Model Age diagrams for the 4th (C) and 3rd (D) centuries BCE. Colours indicate region (Bohemia vs. Moravia), symbols distinguish site categories (cemeteries, Němčice, and Mediterranean coins from Němčice). Purple-highlighted fields mark compositional clusters discussed in the Results section in the main article.

This grouping (“Cluster”; cf. Fig. 1B, 1D) reflects a shift in lead isotope systematics towards greater uniformity, indicating the emergence of more centralised metallurgical traditions and increased reliance on a specific ore province. Artefacts from the **2nd century BCE**, especially from Němčice, display signs of renewed diversification in lead isotope systematics, partially reverting to the more radiogenic compositions typical of the earlier 4th century BCE.

**Fig. 2. Lead isotope (²⁰⁷Pb/²⁰⁴Pb vs. ²⁰⁶Pb/²⁰⁴Pb) and model age vs. Th/U (κ) systematics of studied artefacts.** A–B: Lead isotope ratios for the 4th (A) and 3rd (B) centuries BCE;
C–D: Model age vs. κ (Th/U) plots for the same periods. Colours indicate lead content in wt.% (< 5 %, 5–15 %, > 15 %); symbol types distinguish site categories (cemeteries, Němčice, and Hellenistic coins from Němčice).

### 1.2 Correlation with Lead Content

Lead isotope systematics correlate with lead content (Fig. 2):

- Artefacts with **Pb < 5 wt. %** exhibit widely distributed lead isotope signatures, consistent with unalloyed or mixed-source copper.
- **Pb > 5 wt. %** corresponds increasingly with less radiogenic values in the 3rd century BCE (Cluster), suggesting intentional alloying using galena-type lead ores.
- **2nd-century BCE coins** from Němčice show high Pb contents (up to >40 %) and distinct lead isotope systematics, particularly in Carthaginian material.

## 2. Lead isotope systematics in relation to trace element chemistry

To explore the relationship between isotopic provenance and alloying practices, we examined how lead isotope ratios (²⁰⁶Pb/²⁰⁴Pb, ²⁰⁷Pb/²⁰⁴Pb, ²⁰⁸Pb/²⁰⁴Pb) correspond to the concentration of selected trace elements (Fe, Co, Ni, Zn, Ag, As, Sb, Bi). Bivariate diagrams combining isotopic and chemical data reveal several diachronic and site-specific trends in the 4th and 3rd centuries BCE (Fig. 3-6).

### 2.1 Diachronic shifts in isotopic–chemical relationships

During the 4th century BCE, artefacts show broad dispersion in isotope values, notably along ²⁰⁷Pb/²⁰⁴Pb and ²⁰⁶Pb/²⁰⁴Pb axes (ranging approximately from 18.3 to 18.7 and from 15.55 to 15.70), without any clear clustering. This pattern indicates the use of a diverse range of metal sources, potentially involving mixing, recycling, or informal trade practices. Trace elements such as Sb, As, and Ag—typical indicators of fahlores—are associated with more radiogenic isotope values, suggesting that the use of such ores was closer to the 4th century more radiogenic fields(Fig. 3, Fig. 4).

In contrast, the 3rd century BCE marks a notable compositional shift: a tighter isotopic clustering emerges, especially in artefacts with elevated Pb content (>5 wt. %), forming a coherent field centred around ²⁰⁶Pb/²⁰⁴Pb ≈ 18.4–18.55 and ²⁰⁷Pb/²⁰⁴Pb ≈ 15.61–15.63 (Cluster). This pattern likely reflects the addition of lead from a geologically distinct source, as indicated by isotopic clustering, although this is not accompanied by any distinctive trace element signal in the analysed elements (Fig. 5, Fig. 6).

### 2.2 Correlation between isotope values and alloying elements

Crossplots of isotope ratios with individual trace elements show several recurring patterns:

- **Sb, As, Ag** exhibit higher concentrations in the more radiogenic range, particularly in 4th-century samples, which may suggest the use of fahlores.
- **Ni**, often associated with chalcopyrite-type ores, begins to form more distinct groupings in the 3rd century BCE, aligned with tighter isotopic clusters. **Co**, by contrast, remains more diffusely distributed.

**Fig. 3:** Lead isotope systematics (²⁰⁷Pb/²⁰⁴Pb vs. ²⁰⁶Pb/²⁰⁴Pb) of the 4th century BCE artefacts categorised by selected trace element concentrations (As, Zn, Ag, Ni, Sb, Co, Bi, Fe).

**Fig. 4:** Lead isotope systematics (²⁰⁸Pb/²⁰⁴Pb vs. ²⁰⁶Pb/²⁰⁷Pb) of the 4th century BCE artefacts categorised by selected trace element concentrations (As, Zn, Ag, Ni, Sb, Co, Bi, Fe).

**Fig. 5:** Lead isotope systematics (²⁰⁷Pb/²⁰⁴Pb vs. ²⁰⁶Pb/²⁰⁴Pb) of the 3rd and 2nd century BCE artefacts categorised by selected trace element concentrations (As, Zn, Ag, Ni, Sb, Co, Bi, Fe).

**Fig. 6:** Lead isotope systematics (²⁰⁸Pb/²⁰⁴Pb vs. ²⁰⁶Pb/²⁰⁷Pb) of the 3rd and 2nd century BCE artefacts categorised by selected trace element concentrations (As, Zn, Ag, Ni, Sb, Co, Bi, Fe).

### 2.2 Pb‑isotope distribution of trace‑element PCA clusters (3rd century BCE Moravia)

To further test whether multivariate trace‑element groupings correspond to distinct sources of the lead component, we examined Pb‑isotope ratios (²⁰⁷Pb/²⁰⁴Pb vs. ²⁰⁶Pb/²⁰⁴Pb) for the 3rd‑century BCE Moravian cemeteries and the Němčice nad Hanou agglomeration, coding each artefact by its K‑means cluster derived from the trace‑element PCA (Fig. 8 in main text).

In Pb‑isotope space, the two trace‑element clusters show only partial separation (Fig. 7). Cluster 2 (blue) is concentrated in the less radiogenic part of the dataset (i.e., lower ²⁰⁶Pb/²⁰⁴Pb, broadly < ~18.6), overlapping strongly with the 3rd‑century isotopic field defined in the main text (Cluster). Cluster 1 (red) overlaps with this field but extends more clearly into more radiogenic values (higher ²⁰⁶Pb/²⁰⁴Pb and ²⁰⁷Pb/²⁰⁴Pb). The overlap confirms that trace‑element clustering does not translate into a discrete isotopic clustering, consistent with the interpretation advanced above: the 3rd‑century tightening in Pb‑isotope values primarily reflects the isotopic character of added lead, whereas trace‑element structure continues to capture variability in the copper component, mixing, and/or recycling practices rather than uniquely defining the provenance of lead.

**Fig. 7:** Pb‑isotope ratios (²⁰⁷Pb/²⁰⁴Pb vs. ²⁰⁶Pb/²⁰⁴Pb) for copper‑alloy artefacts from 3rd‑century BCE Moravian cemeteries and the Němčice nad Hanou agglomeration, classified by K‑means clusters (k = 2) obtained from PCA of log‑transformed trace elements (Fe, Co, Ni, Zn, Ag, As, Sb, Bi). Red squares = Cluster 1; blue circles = Cluster 2.

## 3. Site-based variation in lead isotope systematics

To further understand regional patterns in material sourcing and alloying practices, we visualised the lead isotope data by archaeological site. In addition to general clustering (Fig. 8), Figs. 9–12 show isotopic and model-age parameters for individual sites, highlighting their internal variability in contrast to the full dataset, which is shown in grey.

### 3.1 The 4th century BCE: broad dispersion and limited structure

In the **4th century BCE** (Fig. 9 and Fig. 10), most sites exhibit considerable internal spread in both lead isotope ratios (²⁰⁷Pb/²⁰⁴Pb vs. ²⁰⁶Pb/²⁰⁴Pb) and model age parameters (κ vs. MA). Localities such as **Maloměřice**, **Prosmyky**, **Jenišův Újezd**, **Vliněves**, and **Prague** cover almost the full isotopic range observed in the dataset. However, this pattern is at least partly influenced by sample volume—sites with more samples (e.g. Maloměřice, Prosmyky) naturally capture a wider spectrum of values.

Only limited signs of more compact internal behaviour are visible, notably at **Lovčičky**, though this may be primarily a result of the smaller number of available samples rather than evidence of more homogeneous sourcing. The overall picture of the 4th century remains one of diversity, likely reflecting multiple ore sources, alloying traditions, and minimal regional production oragnisation.

### 3.2 The 3rd century BCE: increasing consistency, persistent outliers

In the **3rd century BCE** (Fig. 11 and Fig. 12), the isotopic pattern becomes more structured. Many sites now fall into the previously defined **Cluster**, centred around ²⁰⁶Pb/²⁰⁴Pb ≈ 18.4–18.55 and ²⁰⁷Pb/²⁰⁴Pb ≈ 15.61–15.63. Sites like **Nechvalín**, **Lovčičky**, and **Ponetovice** show relatively narrow isotopic spreads, suggesting more standardised sourcing or workshop-level practices.

Still, significant internal variability persists at some sites—especially **Němčice**, **Mistřín**, which retain artefacts with both more and less radiogenic signatures. This suggests parallel supply systems or recycling practices within more complex economic zones.

The κ–MA diagrams (Fig. 12) largely support this trend. Most 3rd-century sites show clustering around κ ≈ 3.825–3.857 and MA ≈ 150–250 Ma, but outliers are still present—especially at **Mistřín** and **Němčice**, indicating either technological flexibility or continued access to diverse material inputs.

**Fig. 8:** Lead isotope systematics and model age parameters by site.
Panels A and B show ²⁰⁷Pb/²⁰⁴Pb vs. ²⁰⁶Pb/²⁰⁴Pb for artefacts from the 4th century BCE (A) and the 3rd–2nd centuries BCE (B). Panels C and D display κ (Th/U) vs. model age (MA).

**Fig. 9:** Lead isotope systematics (²⁰⁷Pb/²⁰⁴Pb vs. ²⁰⁶Pb/²⁰⁴Pb) of individual sites in the 4th century BCE.

**Fig. 10:** Model age (MA) vs. κ (Th/U) values of individual sites in the 4th century BCE.

**Fig. 11:** Lead isotope systematics (²⁰⁷Pb/²⁰⁴Pb vs. ²⁰⁶Pb/²⁰⁴Pb) of individual sites in the 3rd century BCE.

**Fig. 12:** Model age (MA) vs. κ (Th/U) values of individual sites in the 3rd century BCE.

## 4. The Němčice Agglomeration: Internal Isotopic Trends

The site of Němčice nad Hanou provides a valuable case study for exploring how shifts in metal provenance and alloying practices unfolded across the Middle La Tène period. As a large, well-contextualised settlement and production site, it also offers a critical reference point for comparison with funerary assemblages across Moravia and Bohemia. Phase-by-phase analysis of lead isotope systematics and model parameters reveals a trend from diversity toward increasing standardisation, followed by renewed complexity (Fig. 13, 14).

### 4.1 Phase-wise isotope variation and relationship to Cluster

The **LT B2b–C1 phase** shows isotopic signatures that are not consistent with Cluster, with radiogenic ²⁰⁶Pb/²⁰⁴Pb values (**18.61 ± 0.153**) and a broader range of ²⁰⁷Pb/²⁰⁴Pb values (**15.65 ± 0.024**). These data indicate continued use of diverse or non-standardised sources at the onset of the 3rd century BCE.

The **LT C1 phase** marks a notable convergence in lead isotope ratios, tightly clustering around **18.48 ± 0.058** (²⁰⁶Pb/²⁰⁴Pb) and **15.64 ± 0.021** (²⁰⁷Pb/²⁰⁴Pb). This isotopic homogeneity is mirrored in the model age (**193.8 ± 15.8 Ma**) and κ (**3.88 ± 0.038**), pointing to increasingly standardised sourcing—likely from geologically coherent galena-rich deposits.

The **LT C2 phase** maintains this Cluster alignment (²⁰⁶Pb/²⁰⁴Pb: **18.59 ± 0.185**, ²⁰⁷Pb/²⁰⁴Pb: **15.65 ± 0.022**), but its wide representation across the isotopic field, along with elevated model age variability (**127.0 ± 95.3 Ma**), suggests a **reintroduction of heterogeneity or coexistence of multiple sources**. This may reflect parallel supply systems or less tightly controlled resource flows.

### Table 1. Isotopic and geochemical parameters by phase (Němčice)

| **Phase** | **²⁰⁶Pb/²⁰⁴Pb (avg ± SD)** | **²⁰⁷Pb/²⁰⁴Pb (avg ± SD)** | **Model Age (Ma ± SD)** | **κ (avg ± SD)** | **n** |
| --- | --- | --- | --- | --- | --- |
| LT B2b–C1 | 18.61 ± 0.153 | 15.65 ± 0.024 | 109.5 (n=1) | 3.90 (n=1) | 4 |
| LT B2–C2 | 18.55 ± 0.158 | 15.65 ± 0.024 | 141.2 ± 89.2 | 3.87 ± 0.033 | 17 |
| LT C1 | 18.48 ± 0.058 | 15.64 ± 0.021 | 193.8 ± 15.8 | 3.88 ± 0.038 | 5 |
| LT C2 | 18.59 ± 0.185 | 15.65 ± 0.022 | 127.0 ± 95.3 | 3.85 ± 0.026 | 12 |

**Fig. 13:** Lead isotope systematics (²⁰⁷Pb/²⁰⁴Pb vs. ²⁰⁶Pb/²⁰⁴Pb) of artefacts from the agglomeration of Němčice, categorised by chronological phase.

**Fig. 14:** Model age (MA) vs. Th/U ratio (κ) of artefacts from the agglomeration of Němčice, categorised by chronological phase.

# 5. Comparative biplots of artefact Pb isotopes against ore‑region datasets

**Aim and rationale**
As a complement to the KDE‑based provenance workflow in the main text, we provide traditional biplots that juxtapose artefact Pb‑isotope compositions with reference ore datasets at the scale of major geological units. The plots compare artefacts from the 4th, 3rd and 2nd centuries BCE with ore‑region fields in ²⁰⁷Pb/²⁰⁴Pb vs. ²⁰⁶Pb/²⁰⁴Pb space.
Figs 15–17 show the full set of ore provinces for each century. On this basis, Figs 18–19 zoom in on the Harz and Rhenish Massif ore fields, which provide the closest isotopic matches to the less radiogenic “Cluster” artefacts of the 3rd century BCE.

**Reading guide**

- **Overlap** – extent to which artefacts fall within the dispersion envelope of a given ore field.
- **Trend concordance** – similarity between the orientation of the artefact cloud and the per‑region trend line (regression through ore‑field data).

### Summary of observations (by century)

**4th century BCE (Fig. 15).** Less radiogenic artefacts commonly overlap with the Bohemian Massif and, among nearby provinces, with the Rhenish Massif and the Harz. More radiogenic artefacts intersect Alpine and Carpathian fields.

**3rd century BCE (Fig. 16).** Biplots reflect tighter clustering; many artefacts occupy less radiogenic fields in good agreement with Rhenish Massif/Harz trends, while residual radiogenic items continue to intersect Alpine/Carpathian fields.

**2nd century BCE (Fig. 17).** The overall structure persists, with pockets of renewed diversification at some sites.

### Limitations

Biplots are diagnostic but non-decisive: isotopic overlaps are not unique, ore districts are internally heterogeneous, and mixing/recycling can mimic regional trends. These graphics are intended as a transparent visual audit to be used alongside the probabilistic results.

### Figures:

**(see next page)**

**Fig. 15a.** Lead isotope systematics (²⁰⁷Pb/²⁰⁴Pb vs. ²⁰⁶Pb/²⁰⁴Pb) of the **4th century BCE** artefacts (black) vs various ore fields (green), with trendlines for both datasets – part A.

**Fig. 15b.** Lead isotope systematics (²⁰⁷Pb/²⁰⁴Pb vs. ²⁰⁶Pb/²⁰⁴Pb) of the **4th century BCE** artefacts (black) vs various ore fields (green), with trendlines for both datasets – part B.

**Fig. 15c.** Lead isotope systematics (²⁰⁷Pb/²⁰⁴Pb vs. ²⁰⁶Pb/²⁰⁴Pb) of the **4th century BCE** artefacts (black) vs various ore fields (green), with trendlines for both datasets – part C.

**Fig. 16a.** Lead isotope systematics (²⁰⁷Pb/²⁰⁴Pb vs. ²⁰⁶Pb/²⁰⁴Pb) of the **3rd century BCE** artefacts (black) vs various ore fields (green), with trendlines for both datasets – part A.

**Fig. 16b.** Lead isotope systematics (²⁰⁷Pb/²⁰⁴Pb vs. ²⁰⁶Pb/²⁰⁴Pb) of the **3rd century BCE** artefacts (black) vs various ore fields (green), with trendlines for both datasets – part B.

**Fig. 16c.** Lead isotope systematics (²⁰⁷Pb/²⁰⁴Pb vs. ²⁰⁶Pb/²⁰⁴Pb) of the **3rd century BCE** artefacts (black) vs various ore fields (green), with trendlines for both datasets – part C.

**Fig. 17a.** Lead isotope systematics (²⁰⁷Pb/²⁰⁴Pb vs. ²⁰⁶Pb/²⁰⁴Pb) of the **2nd century BCE** artefacts (black) vs various ore fields (green), with trendlines for both datasets – part A.

**Fig. 17b.** Lead isotope systematics (²⁰⁷Pb/²⁰⁴Pb vs. ²⁰⁶Pb/²⁰⁴Pb) of the **2nd century BCE** artefacts (black) vs various ore fields (green), with trendlines for both datasets – part B.

**Fig. 17c.** Lead isotope systematics (²⁰⁷Pb/²⁰⁴Pb vs. ²⁰⁶Pb/²⁰⁴Pb) of the **2nd century BCE** artefacts (black) vs various ore fields (green), with trendlines for both datasets – part C.

**Fig. 18.** Lead isotope systematics (²⁰⁷Pb/²⁰⁴Pb vs. ²⁰⁶Pb/²⁰⁴Pb) of 3rd-century BCE artefacts (black) compared to ore-field datasets from the Harz Mountains (coloured fields), with trendlines shown for both datasets.

**Fig. 19a.** Lead isotope systematics (²⁰⁷Pb/²⁰⁴Pb vs. ²⁰⁶Pb/²⁰⁴Pb) of 3rd-century BCE artefacts (black) compared with ore-field datasets from the various regions of the Rhenish Massif (coloured fields), with trendlines shown for both datasets – part A.

**Fig. 19b.** Lead isotope systematics (²⁰⁷Pb/²⁰⁴Pb vs. ²⁰⁶Pb/²⁰⁴Pb) of 3rd-century BCE artefacts (black) compared with ore-field datasets from the various regions of the Rhenish Massif (coloured fields), with trendlines shown for both datasets – part B.
