# Supplementary material for: Tracing socioeconomic change and emerging elites in 4th–3rd century BCE: Metalwork from the Amber Road Corridor
Source: PLoS One. 2026 Sep 2;21(9):e0352885. doi: 10.1371/journal.pone.0352885 (PMC13537607; doi:10.1371/journal.pone.0352885)
Supplement: Peer review history

# Peer review: Decision Letter 0

**DOI:** 10.1371/journal.pone.0352885.r001

**Authors:** Carlos Odriozola

10 Apr 2026

Dear Dr. Danielisová,

Thank you for submitting your manuscript to PLOS ONE. After careful consideration, we feel that it has merit but does not fully meet PLOS ONE’s publication criteria as it currently stands. Therefore, we invite you to submit a revised version of the manuscript that addresses the points raised during the review process.

plosone@plos.org. When you're ready to submit your revision, log on to https://www.editorialmanager.com/pone/ and select the 'Submissions Needing Revision' folder to locate your manuscript file.

- A letter that responds to each point raised by the academic editor and reviewer(s). You should upload this letter as a separate file labeled 'Response to Reviewers'.
- A marked-up copy of your manuscript that highlights changes made to the original version. You should upload this as a separate file labeled 'Revised Manuscript with Track Changes'.
- An unmarked version of your revised paper without tracked changes. You should upload this as a separate file labeled 'Manuscript'.

If you would like to make changes to your financial disclosure, please include your updated statement in your cover letter. Guidelines for resubmitting your figure files are available below the reviewer comments at the end of this letter.

If applicable, we recommend that you deposit your laboratory protocols in protocols.io to enhance the reproducibility of your results. Protocols.io assigns your protocol its own identifier (DOI) so that it can be cited independently in the future. For instructions see: https://journals.plos.org/plosone/s/submission-guidelines#loc-laboratory-protocols. Additionally, PLOS ONE offers an option for publishing peer-reviewed Lab Protocol articles, which describe protocols hosted on protocols.io. Read more information on sharing protocols at https://plos.org/protocols?utm_medium=editorial-email&utm_source=authorletters&utm_campaign=protocols.

As the corresponding author, your ORCID iD is verified in the submission system and will appear in the published article. PLOS supports the use of ORCID, and we encourage all coauthors to register for an ORCID iD and use it as well. Please encourage your coauthors to verify their ORCID iD within the submission system before final acceptance, as unverified ORCID iDs will not appear in the published article. *Only*  the individual author can complete the verification step; PLOS staff *cannot*  verify ORCID iDs on behalf of authors.

We look forward to receiving your revised manuscript.

Kind regards,

Carlos P. Odriozola, Ph.D

Academic Editor

PLOS One

Journal Requirements:

When submitting your revision, we need you to address these additional requirements.

1. Please ensure that your manuscript meets PLOS ONE's style requirements, including those for file naming. The PLOS ONE style templates can be found at

https://journals.plos.org/plosone/s/file?id=wjVg/PLOSOne_formatting_sample_main_body.pdf and

https://journals.plos.org/plosone/s/file?id=ba62/PLOSOne_formatting_sample_title_authors_affiliations.pdf

2. Please include a complete copy of PLOS’ questionnaire on inclusivity in global research in your revised manuscript. Our policy for research in this area aims to improve transparency in the reporting of research performed outside of researchers’ own country or community. The policy applies to researchers who have travelled to a different country to conduct research, research with Indigenous populations or their lands, and research on cultural artefacts. The questionnaire can also be requested at the journal’s discretion for any other submissions, even if these conditions are not met.  Please find more information on the policy and a link to download a blank copy of the questionnaire here: https://journals.plos.org/plosone/s/best-practices-in-research-reporting. Please upload a completed version of your questionnaire as Supporting Information when you resubmit your manuscript.

3. In your manuscript, please provide additional information regarding the specimens used in your study. Ensure that you have reported human remain specimen numbers and complete repository information, including museum name and geographic location.

If permits were required, please ensure that you have provided details for all permits that were obtained, including the full name of the issuing authority, and add the following statement:

'All necessary permits were obtained for the described study, which complied with all relevant regulations.'

If no permits were required, please include the following statement:

'No permits were required for the described study, which complied with all relevant regulations.'

For more information on PLOS One's requirements for paleontology and archeology research, see https://journals.plos.org/plosone/s/submission-guidelines#loc-paleontology-and-archaeology-research.

3. Please amend your manuscript to include your abstract after the title page.

4. Please ensure that you refer to Figure 2 in your text as, if accepted, production will need this reference to link the reader to the figure.

5. We note that Figures 1 and 13 in your submission contain map images which may be copyrighted. All PLOS content is published under the Creative Commons Attribution License (CC BY 4.0), which means that the manuscript, images, and Supporting Information files will be freely available online, and any third party is permitted to access, download, copy, distribute, and use these materials in any way, even commercially, with proper attribution. For these reasons, we cannot publish previously copyrighted maps or satellite images created using proprietary data, such as Google software (Google Maps, Street View, and Earth). For more information, see our copyright guidelines: http://journals.plos.org/plosone/s/licenses-and-copyright.

We require you to either (1) present written permission from the copyright holder to publish these figures specifically under the CC BY 4.0 license, or (2) remove the figures from your submission:

1. You may seek permission from the original copyright holder of Figure(s) [#] to publish the content specifically under the CC BY 4.0 license.

We recommend that you contact the original copyright holder with the Content Permission Form (http://journals.plos.org/plosone/s/file?id=7c09/content-permission-form.pdf) and the following text:

“I request permission for the open-access journal PLOS ONE to publish XXX under the Creative Commons Attribution License (CCAL) CC BY 4.0 (http://creativecommons.org/licenses/by/4.0/). Please be aware that this license allows unrestricted use and distribution, even commercially, by third parties. Please reply and provide explicit written permission to publish XXX under a CC BY license and complete the attached form.”

Please upload the completed Content Permission Form or other proof of granted permissions as an "Other" file with your submission.

In the figure caption of the copyrighted figure, please include the following text: “Reprinted from [ref] under a CC BY license, with permission from [name of publisher], original copyright [original copyright year].”

2. If you are unable to obtain permission from the original copyright holder to publish these figures under the CC BY 4.0 license or if the copyright holder’s requirements are incompatible with the CC BY 4.0 license, please either i) remove the figure or ii) supply a replacement figure that complies with the CC BY 4.0 license. Please check copyright information on all replacement figures and update the figure caption with source information. If applicable, please specify in the figure caption text when a figure is similar but not identical to the original image and is therefore for illustrative purposes only.

The following resources for replacing copyrighted map figures may be helpful:

USGS National Map Viewer (public domain): http://viewer.nationalmap.gov/viewer/

The Gateway to Astronaut Photography of Earth (public domain): http://eol.jsc.nasa.gov/sseop/clickmap/

Maps at the CIA (public domain): https://www.cia.gov/library/publications/the-world-factbook/index.html and https://www.cia.gov/library/publications/cia-maps-publications/index.html

NASA Earth Observatory (public domain): http://earthobservatory.nasa.gov/

Landsat: http://landsat.visibleearth.nasa.gov/

USGS EROS (Earth Resources Observatory and Science (EROS) Center) (public domain): http://eros.usgs.gov/#

Natural Earth (public domain): http://www.naturalearthdata.com/

6. We note that Figure 2 in your submission contain copyrighted images. All PLOS content is published under the Creative Commons Attribution License (CC BY 4.0), which means that the manuscript, images, and Supporting Information files will be freely available online, and any third party is permitted to access, download, copy, distribute, and use these materials in any way, even commercially, with proper attribution. For more information, see our copyright guidelines: http://journals.plos.org/plosone/s/licenses-and-copyright.

We require you to either (1) present written permission from the copyright holder to publish these figures specifically under the CC BY 4.0 license, or (2) remove the figures from your submission:

1. You may seek permission from the original copyright holder of Figure(s) [#] to publish the content specifically under the CC BY 4.0 license.

We recommend that you contact the original copyright holder with the Content Permission Form (http://journals.plos.org/plosone/s/file?id=7c09/content-permission-form.pdf) and the following text:

“I request permission for the open-access journal PLOS ONE to publish XXX under the Creative Commons Attribution License (CCAL) CC BY 4.0 (http://creativecommons.org/licenses/by/4.0/). Please be aware that this license allows unrestricted use and distribution, even commercially, by third parties. Please reply and provide explicit written permission to publish XXX under a CC BY license and complete the attached form.”

Please upload the completed Content Permission Form or other proof of granted permissions as an "Other" file with your submission.

In the figure caption of the copyrighted figure, please include the following text: “Reprinted from [ref] under a CC BY license, with permission from [name of publisher], original copyright [original copyright year].”

2. If you are unable to obtain permission from the original copyright holder to publish these figures under the CC BY 4.0 license or if the copyright holder’s requirements are incompatible with the CC BY 4.0 license, please either i) remove the figure or ii) supply a replacement figure that complies with the CC BY 4.0 license. Please check copyright information on all replacement figures and update the figure caption with source information. If applicable, please specify in the figure caption text when a figure is similar but not identical to the original image and is therefore for illustrative purposes only.

7. Please include captions for your Supporting Information files at the end of your manuscript, and update any in-text citations to match accordingly. Please see our Supporting Information guidelines for more information: http://journals.plos.org/plosone/s/supporting-information.

8. If the reviewer comments include a recommendation to cite specific previously published works, please review and evaluate these publications to determine whether they are relevant and should be cited. There is no requirement to cite these works unless the editor has indicated otherwise.

9. Please review your reference list to ensure that it is complete and correct. If you have cited papers that have been retracted, please include the rationale for doing so in the manuscript text, or remove these references and replace them with relevant current references. Any changes to the reference list should be mentioned in the rebuttal letter that accompanies your revised manuscript. If you need to cite a retracted article, indicate the article’s retracted status in the References list and also include a citation and full reference for the retraction notice.

[Note: HTML markup is below. Please do not edit.]

Reviewers' comments:

Reviewer's Responses to Questions

**Comments to the Author**

1. Is the manuscript technically sound, and do the data support the conclusions?

Reviewer #1: Yes

Reviewer #2: Yes

**********

2. Has the statistical analysis been performed appropriately and rigorously?

Reviewer #1: Yes

Reviewer #2: Yes

**********

3. Have the authors made all data underlying the findings in their manuscript fully available?

The PLOS Data policy

Reviewer #1: Yes

Reviewer #2: Yes

**********

4. Is the manuscript presented in an intelligible fashion and written in standard English?

Reviewer #1: Yes

Reviewer #2: Yes

**********

Reviewer #1: “Tracing Socioeconomic Change and Emerging and her colleagues Elites in 4th–3rd Century BCE: Metalwork from the Amber Road Corridor” by Danielisová is a well written, well organized, well-reasoned, and very data rich study. As such, in my opinion, it certainly deserves to be published by PLOS One.

I feel that the text and data are exemplary. My only criticism lies within the data presentation graphics. Such large data set are a blessing in the richness of the interpretations that can be extracted. However, they are a curse in terms of presenting them clearly. In general, too much information is being conveyed in figures that are too few and too small. This is particularly true for figures 4-8 and 12. These need to be reworked, and subdivided, and both data points and labels enlarged. I have outlined specific graphic related issues below.

Fig. 4 - It is not clear from the figure alone, what the apices represent. I suggest replacing the true labels (e.g. Co+Ni) rather than L, T, R, which are not intuitive. Also figures should be labelled with wt% rather than just %. What is the difference between the 2nd and 3rd plots, both are labelled as 3rd cent, BCE and have the same legend.

Fig. 5. – Having the same color and symbol scheme for completely different variables in A and B is initially misleading. Different symbols/colors should be used between them. There is simply too many groups plotted in 5C and 5D for the reader to readily see and interpret the patterns. Perhaps they should be split into two each (C: one for Bohemia and one for Moravia). I am not sure how to split D, but at the very least the time periods should be in sequence and one should be able to readily distinguish the similar time periods (e.g. LtB2, B2a, B2b should share a color or symbol).

Fig. 6 – Again too many data points in 5C f9r easy reading. Perhaps split into Boh sites and Mor sites

Fig 8. I had to zoom in on the image extensively to distinguish between groups, and this is made worse by the number of colors used. Break the plot into subplots with fewer sites in each. Note, the following Figs 9, 10, 11, 12 in S3 conveyed far more information to me than the summative figure 8.

Fig. 12 Text is two small to read on X-axis labels. Pb% legend should be inserted into figure.

Lines 247-248 : “the latter showing a tendency toward a more homogeneous pattern which is more evident towards the 3rd century BCE (Fig. 5.B, Fig. 7.A), especially in phases LT B2b-C1 and LT C1 (Fig. 5.D).” I do not see this in the figures. In 7A, Bohemia appears more clustered with the exception of two outliers, and 5B does not distinguish between Moravian and Bohemian artifacts.

Line 259: “The most marked differences are visible among the regions (Fig 6.A)” . I do not see any variation in clustering between the two localities.

Reviewer #2: The authors argue that during the 4th–3rd centuries BCE in Central Europe, there was a diachronic shift from heterogeneous, locally mediated copper-alloying practices to a highly standardized mass production of ‘leaded bronze’. Crucially, the authors claim that this innovation was first adopted by rural communities (visible in cemetery contexts) and only later scaled up by large open agglomeration sites like Němčice nad Hanou. Additionally, their provenance analysis suggests that the raw material (lead) for this surge in production was sourced predominantly from Transalpine Variscan deposits (like the Harz Mountains and Rhenish Massif) rather than Mediterranean imports. Finally, they connect this technological shift to the emergence of ‘bulk luxury’ goods (the voluminous ‘Plastic-Style’ jewellery) that helped form new socio-economic elite configurations.

These claims are highly significant for Iron Age archaeology. The prevailing narrative often assumes a ‘top-down’ model where centralized, wealthy agglomeration sites drove technological and economic innovation before it trickled down to rural areas. This study turns that model on its head, using hard archaeometry to position rural communities as the initial agents of change.

The authors place their findings directly within current debates regarding Iron Age centralization, socio-economic complexity, and technological transitions. They appropriately cite existing literature on Central European agglomeration sites (e.g., Roseldorf, Haselbach, Němčice) and artistic developments like the ‘Plastic-Style’. The literature on lead isotope provenance (e.g., OXALID, GlobaLID) is also treated robustly and fairly utilized.

The data and analyses strongly support the claims. The authors analysed a substantial dataset of 399 samples. Their chronological cross-section effectively demonstrates the shift: 1) Principal Component Analysis (PCA) and trace element ternary graphs clearly show 4th-century alloys were highly dispersed, while 3rd-century alloys tightly clustered. 2) The comparison between early 3rd-century rural graves and later agglomeration materials supports the timeline of rural-first adoption. 3) Isotopic Kernel Density Estimation modelling validates the narrowing of supply chains to specific geological signatures. No additional primary data is required, as the analytical pipeline is exhaustive.

The authors utilize custom pipelines for non-parametric statistical evaluation (Kernel Density Estimations) and multivariate analysis (PCA, K-means clustering). They have adhered to PLOS One's best practices by stating that all individual statistical codes (RStudio and Python) are available in the Supplementary Information 5 (S5 Text). Additionally, they compiled a 12,741-sample lead isotope database (LIDOF), which they appropriately provide in S4 Table.

The manuscript appears highly suitable for publication in its current form, pending only standard peer-review revisions.

The authors explicitly state that ‘All relevant data are within the manuscript and its Supporting Information files,’ fulfilling repository requirements for this type of dataset.

The methodology is described with sufficient technical precision. The authors detail their sampling technique (extracting 30-50 mg from the uncorroded metal core) and instrumentation (NEPTUNE Plus MC-ICP-MS and standard ICP-MS). They also provide exact algorithmic parameters for their computational modelling, such as the predefined integration ranges for isotopic values (±0.01 for ²⁰⁶Pb/²⁰⁴Pb and ²⁰⁷Pb/²⁰⁴Pb, ±0.03 for ²⁰⁸Pb/²⁰⁴Pb) and the 10% probability threshold used for KDE filtering.

The custom R and Python scripts used for evaluating the dataset are provided in the supplementary file "S5 Text".

The manuscript is logically organized and written in excellent English. While sections of the text (like the isotopic provenance analysis and PCA data structuring) are dense and highly technical, the authors do a good job in the Introduction and Discussion sections translating these geochemical findings into clear, accessible socio-economic and historical implications that a general archaeologist can understand.

Finally, I have two queries intended to help the authors expand their Discussion section, encouraging them to complement their strong archaeometric analysis with a more explicit anthropological and socio‑economic framing:

1) Comparing funerary (use/deposition) vs. agglomeration (manufacture) contexts: The study effectively contrasts 69 samples from the open industrial agglomeration at Němčice nad Hanou with samples from rural flat cemeteries. However, this compares two distinct behavioral spheres: ritualized use-deposition (curated grave goods) versus daily settlement/manufacturing discard. Could the observed shift toward standardized leaded bronzes in the 3rd-century BCE cemeteries partly reflect a cultural or ritual preference for burying specific types of objects (e.g., the voluminous 'Plastic-Style' jewelry), rather than a complete picture of the communities' daily metallurgical reality? The authors should discuss more explicitly how the taphonomic and socio-economic behaviors unique to funerary deposition versus industrial production might mediate these isotopic and chemical signals.

2) The socio-economic model, ‘warrior elites’, and amber: The authors propose a compelling socio-economic model regarding the emergence of ‘bulk luxury’ commodities, attributing the coordination of this economy to ‘local warrior elites’. However, the specific mechanisms of this control remain somewhat abstract. Could the authors clarify the role of these elites in relation to the metallurgical specialists? Were these artisans attached directly to elite households, or were they independent specialists operating within community networks? Furthermore, given that the paper is titled 'Metalwork from the Amber Road Corridor', the actual socio-economic integration of amber is underexplored in the text. While sapropelite and glass are effectively used as analogies for bulk luxuries, how did the north-south flow of Baltic amber specifically articulate with the east-west/north-south flows of Rhenohercynian lead and Alpine/Carpathian copper?

**********

what does this mean?). If published, this will include your full peer review and any attached files.

If you choose “no”, your identity will remain anonymous but your review may still be made public.

**Do you want your identity to be public for this peer review?**  For information about this choice, including consent withdrawal, please see our Privacy Policy

Reviewer #1: No

Reviewer #2: No

**********

[NOTE: If reviewer comments were submitted as an attachment file, they will be attached to this email and accessible via the submission site. Please log into your account, locate the manuscript record, and check for the action link "View Attachments". If this link does not appear, there are no attachment files.]

To ensure your figures meet our technical requirements, please review our figure guidelines: https://journals.plos.org/plosone/s/figures

You may also use PLOS’s free figure tool, NAAS, to help you prepare publication quality figures: https://journals.plos.org/plosone/s/figures#loc-tools-for-figure-preparation.

NAAS will assess whether your figures meet our technical requirements by comparing each figure against our figure specifications.

# Author comment: Author response to Decision Letter 1

**DOI:** 10.1371/journal.pone.0352885.r002

9 Jun 2026

Journal Requirements:

When submitting your revision, we need you to address these additional requirements.

1. Please ensure that your manuscript meets PLOS ONE's style requirements, including those for file naming. The PLOS ONE style templates can be found at

https://journals.plos.org/plosone/s/file?id=wjVg/PLOSOne_formatting_sample_main_body.pdf and

https://journals.plos.org/plosone/s/file?id=ba62/PLOSOne_formatting_sample_title_authors_affiliations.pdf

This has been adhered to.

2. Please include a complete copy of PLOS’ questionnaire on inclusivity in global research in your revised manuscript. Our policy for research in this area aims to improve transparency in the reporting of research performed outside of researchers’ own country or community. The policy applies to researchers who have travelled to a different country to conduct research, research with Indigenous populations or their lands, and research on cultural artefacts. The questionnaire can also be requested at the journal’s discretion for any other submissions, even if these conditions are not met. Please find more information on the policy and a link to download a blank copy of the questionnaire here: https://journals.plos.org/plosone/s/best-practices-in-research-reporting. Please upload a completed version of your questionnaire as Supporting Information when you resubmit your manuscript.

The questionnaire was uploaded as requested.

3. In your manuscript, please provide additional information regarding the specimens used in your study. Ensure that you have reported human remain specimen numbers and complete repository information, including museum name and geographic location.

If permits were required, please ensure that you have provided details for all permits that were obtained, including the full name of the issuing authority, and add the following statement:

'All necessary permits were obtained for the described study, which complied with all relevant regulations.'

If no permits were required, please include the following statement:

'No permits were required for the described study, which complied with all relevant regulations.'

Information on museum name and location plus this statement are in the Manuscript on page 4.

For more information on PLOS One's requirements for paleontology and archeology research, see https://journals.plos.org/plosone/s/submission-guidelines#loc-paleontology-and-archaeology-research.

3. Please amend your manuscript to include your abstract after the title page.

Amended.

4. Please ensure that you refer to Figure 2 in your text as, if accepted, production will need this reference to link the reader to the figure.

Corrected

5. We note that Figures 1 and 13 in your submission contain map images which may be copyrighted. All PLOS content is published under the Creative Commons Attribution License (CC BY 4.0), which means that the manuscript, images, and Supporting Information files will be freely available online, and any third party is permitted to access, download, copy, distribute, and use these materials in any way, even commercially, with proper attribution. For these reasons, we cannot publish previously copyrighted maps or satellite images created using proprietary data, such as Google software (Google Maps, Street View, and Earth). For more information, see our copyright guidelines: http://journals.plos.org/plosone/s/licenses-and-copyright.

We require you to either (1) present written permission from the copyright holder to publish these figures specifically under the CC BY 4.0 license, or (2) remove the figures from your submission:

We replaced these figures with new version with underlying maps adhering to CC BY 4.0 license (DMR 5G Map by ČUZK, 2026, www.cuzk.gov.cz, CC-BY 4.0).

6. We note that Figure 2 in your submission contain copyrighted images. All PLOS content is published under the Creative Commons Attribution License (CC BY 4.0), which means that the manuscript, images, and Supporting Information files will be freely available online, and any third party is permitted to access, download, copy, distribute, and use these materials in any way, even commercially, with proper attribution. For more information, see our copyright guidelines: http://journals.plos.org/plosone/s/licenses-and-copyright.

The required permission was obtained, and the form was uploaded together with the revised manuscript.

7. Please include captions for your Supporting Information files at the end of your manuscript, and update any in-text citations to match accordingly. Please see our Supporting Information guidelines for more information: http://journals.plos.org/plosone/s/supporting-information.

Captions were included.

8. If the reviewer comments include a recommendation to cite specific previously published works, please review and evaluate these publications to determine whether they are relevant and should be cited. There is no requirement to cite these works unless the editor has indicated otherwise.

9. Please review your reference list to ensure that it is complete and correct. If you have cited papers that have been retracted, please include the rationale for doing so in the manuscript text, or remove these references and replace them with relevant current references. Any changes to the reference list should be mentioned in the rebuttal letter that accompanies your revised manuscript. If you need to cite a retracted article, indicate the article’s retracted status in the References list and also include a citation and full reference for the retraction notice.

[Note: HTML markup is below. Please do not edit.]

Reviewers' comments:

Reviewer's Responses to Questions

Comments to the Author

1. Is the manuscript technically sound, and do the data support the conclusions?

The manuscript must describe a technically sound piece of scientific research with data that supports the conclusions. Experiments must have been conducted rigorously, with appropriate controls, replication, and sample sizes. The conclusions must be drawn appropriately based on the data presented.

Reviewer #1: Yes

Reviewer #2: Yes

________________________________________

2. Has the statistical analysis been performed appropriately and rigorously?

Reviewer #1: Yes

Reviewer #2: Yes

________________________________________

3. Have the authors made all data underlying the findings in their manuscript fully available?

The PLOS Data policy requires authors to make all data underlying the findings described in their manuscript fully available without restriction, with rare exception (please refer to the Data Availability Statement in the manuscript PDF file). The data should be provided as part of the manuscript or its supporting information, or deposited to a public repository. For example, in addition to summary statistics, the data points behind means, medians and variance measures should be available. If there are restrictions on publicly sharing data—e.g. participant privacy or use of data from a third party—those must be specified.

Reviewer #1: Yes

Reviewer #2: Yes

________________________________________

4. Is the manuscript presented in an intelligible fashion and written in standard English?

PLOS ONE does not copyedit accepted manuscripts, so the language in submitted articles must be clear, correct, and unambiguous. Any typographical or grammatical errors should be corrected at revision, so please note any specific errors here.

Reviewer #1: Yes

Reviewer #2: Yes

________________________________________

5. Review Comments to the Author

Please use the space provided to explain your answers to the questions above. You may also include additional comments for the author, including concerns about dual publication, research ethics, or publication ethics. (Please upload your review as an attachment if it exceeds 20,000 characters)

Reviewer #1: “Tracing Socioeconomic Change and Emerging and her colleagues Elites in 4th–3rd Century BCE: Metalwork from the Amber Road Corridor” by Danielisová is a well written, well organized, well-reasoned, and very data rich study. As such, in my opinion, it certainly deserves to be published by PLOS One.

I feel that the text and data are exemplary. My only criticism lies within the data presentation graphics. Such large data set are a blessing in the richness of the interpretations that can be extracted. However, they are a curse in terms of presenting them clearly. In general, too much information is being conveyed in figures that are too few and too small. This is particularly true for figures 4-8 and 12. These need to be reworked, and subdivided, and both data points and labels enlarged. I have outlined specific graphic related issues below.

Fig. 4 - It is not clear from the figure alone, what the apices represent. I suggest replacing the true labels (e.g. Co+Ni) rather than L, T, R, which are not intuitive. Also figures should be labelled with wt% rather than just %. What is the difference between the 2nd and 3rd plots, both are labelled as 3rd cent, BCE and have the same legend.

The original figure was replaced by a new version adhering to the reviewer´s comment.

Fig. 5. – Having the same color and symbol scheme for completely different variables in A and B is initially misleading. Different symbols/colors should be used between them. There is simply too many groups plotted in 5C and 5D for the reader to readily see and interpret the patterns. Perhaps they should be split into two each (C: one for Bohemia and one for Moravia). I am not sure how to split D, but at the very least the time periods should be in sequence and one should be able to readily distinguish the similar time periods (e.g. LtB2, B2a, B2b should share a color or symbol).

The original figure was replaced by a new version adhering to the reviewer´s comment.

Fig. 6 – Again too many data points in 5C f9r easy reading. Perhaps split into Boh sites and Mor sites

The original figure was replaced by a new version adhering to the reviewer´s comment.

Fig 8. I had to zoom in on the image extensively to distinguish between groups, and this is made worse by the number of colors used. Break the plot into subplots with fewer sites in each. Note, the following Figs 9, 10, 11, 12 in S3 conveyed far more information to me than the summative figure 8.

The original figure was replaced by a new version adhering to the reviewer´s comment. Additional figures were added to the Supplementary Information (SI) 2.

Fig. 12 Text is two small to read on X-axis labels. Pb% legend should be inserted into figure.

The original figure was replaced by a new version adhering to the reviewer´s comment.

Lines 247-248 : “the latter showing a tendency toward a more homogeneous pattern which is more evident towards the 3rd century BCE (Fig. 5.B, Fig. 7.A), especially in phases LT B2b-C1 and LT C1 (Fig. 5.D).” I do not see this in the figures. In 7A, Bohemia appears more clustered with the exception of two outliers, and 5B does not distinguish between Moravian and Bohemian artifacts.

Corrected in the text to make it clearer.

Line 259: “The most marked differences are visible among the regions (Fig 6.A)” . I do not see any variation in clustering between the two localities.

Corrected in the text to make it clearer.

Reviewer #2: The authors argue that during the 4th–3rd centuries BCE in Central Europe, there was a diachronic shift from heterogeneous, locally mediated copper-alloying practices to a highly standardized mass production of ‘leaded bronze’. Crucially, the authors claim that this innovation was first adopted by rural communities (visible in cemetery contexts) and only later scaled up by large open agglomeration sites like Němčice nad Hanou. Additionally, their provenance analysis suggests that the raw material (lead) for this surge in production was sourced predominantly from Transalpine Variscan deposits (like the Harz Mountains and Rhenish Massif) rather than Mediterranean imports. Finally, they connect this technological shift to the emergence of ‘bulk luxury’ goods (the voluminous ‘Plastic-Style’ jewellery) that helped form new socio-economic elite configurations.

These claims are highly significant for Iron Age archaeology. The prevailing narrative often assumes a ‘top-down’ model where centralized, wealthy agglomeration sites drove technological and economic innovation before it trickled down to rural areas. This study turns that model on its head, using hard archaeometry to position rural communities as the initial agents of change.

The authors place their findings directly within current debates regarding Iron Age centralization, socio-economic complexity, and technological transitions. They appropriately cite existing literature on Central European agglomeration sites (e.g., Roseldorf, Haselbach, Němčice) and artistic developments like the ‘Plastic-Style’. The literature on lead isotope provenance (e.g., OXALID, GlobaLID) is also treated robustly and fairly utilized.

The data and analyses strongly support the claims. The authors analysed a substantial dataset of 399 samples. Their chronological cross-section effectively demonstrates the shift: 1) Principal Component Analysis (PCA) and trace element ternary graphs clearly show 4th-century alloys were highly dispersed, while 3rd-century alloys tightly clustered. 2) The comparison between early 3rd-century rural graves and later agglomeration materials supports the timeline of rural-first adoption. 3) Isotopic Kernel Density Estimation modelling validates the narrowing of supply chains to specific geological signatures. No additional primary data is required, as the analytical pipeline is exhaustive.

The authors utilize custom pipelines for non-parametric statistical evaluation (Kernel Density Estimations) and multivariate analysis (PCA, K-means clustering). They have adhered to PLOS One's best practices by stating that all individual statistical codes (RStudio and Python) are available in the Supplementary Information 5 (S5 Text). Additionally, they compiled a 12,741-sample lead isotope database (LIDOF), which they appropriately provide in S4 Table.

The manuscript appears highly suitable for publication in its current form, pending only standard peer-review revisions.

The authors explicitly state that ‘All relevant data are within the manuscript and its Supporting Information files,’ fulfilling repository requirements for this type of dataset.

The methodology is described with sufficient technical precision. The authors detail their sampling technique (extracting 30-50 mg from the uncorroded metal core) and instrumentation (NEPTUNE Plus MC-ICP-MS and standard ICP-MS). They also provide exact algorithmic parameters for their computational modelling, such as the predefined integration ranges for isotopic values (±0.01 for ²⁰⁶Pb/²⁰⁴Pb and ²⁰⁷Pb/²⁰⁴Pb, ±0.03 for ²⁰⁸Pb/²⁰⁴Pb) and the 10% probability threshold used for KDE filtering.

The custom R and Python scripts used for evaluating the dataset are provided in the supplementary file "S5 Text".

The manuscript is logically organized and written in excellent English. While sections of the text (like the isotopic provenance analysis and PCA data structuring) are dense and highly technical, the authors do a good job in the Introduction and Discussion sections translating these geochemical findings into clear, accessible socio-economic and historical implications that a general archaeologist can understand.

Finally, I have two queries intended to help the authors expand their Discussion section, encouraging them to complement their strong archaeometric analysis with a more explicit anthropological and socio-economic framing:

1) Comparing funerary (use/deposition) vs. agglomeration (manufacture) contexts: The study effectively contrasts 69 samples from the open industrial agglomeration at Němčice nad Hanou with samples from rural flat cemeteries. However, this compares two distinct behavioral spheres: ritualized use-deposition (curated grave goods) versus daily settlement/manufacturing discard. Could the observed shift toward standardized leaded bronzes in the 3rd-century BCE cemeteries partly reflect a cultural or ritual preference for burying specific types of objects (e

Attachment Submitted filename: Response to Reviewers.docx

# Editor report: Decision Letter 1

**DOI:** 10.1371/journal.pone.0352885.r003

**Authors:** Carlos Odriozola

17 Jun 2026

Tracing Socioeconomic Change and Emerging Elites in 4th–3rd Century BCE: Metalwork from the Amber Road Corridor

PONE-D-25-67886R1

Dear Dr. Danielisová,

We’re pleased to inform you that your manuscript has been judged scientifically suitable for publication and will be formally accepted for publication once it meets all outstanding technical requirements.

Within one week, you’ll receive an e-mail detailing the required amendments. When these have been addressed, you’ll receive a formal acceptance letter and your manuscript will be scheduled for publication.

An invoice will be generated when your article is formally accepted. Please note, if your institution has a publishing partnership with PLOS and your article meets the relevant criteria, all or part of your publication costs will be covered. Please make sure your user information is up-to-date by logging into Editorial Manager at Editorial Manager® and clicking the ‘Update My Information' link at the top of the page. For questions related to billing, please contact billing support.

If your institution or institutions have a press office, please notify them about your upcoming paper to help maximize its impact. If they’ll be preparing press materials, please inform our press team as soon as possible -- no later than 48 hours after receiving the formal acceptance. Your manuscript will remain under strict press embargo until 2 pm Eastern Time on the date of publication. For more information, please contact onepress@plos.org.

Kind regards,

Carlos P. Odriozola, Ph.D

Academic Editor

PLOS One

Additional Editor Comments (optional):

Reviewers' comments:

# Editor report: Acceptance letter

**DOI:** 10.1371/journal.pone.0352885.r004

**Authors:** Carlos Odriozola

PONE-D-25-67886R1

PLOS One

Dear Dr. Danielisová,

I'm pleased to inform you that your manuscript has been deemed suitable for publication in PLOS One. Congratulations! Your manuscript is now being handed over to our production team.

At this stage, our production department will prepare your paper for publication. This includes ensuring the following:

* All references, tables, and figures are properly cited

* All relevant supporting information is included in the manuscript submission,

* There are no issues that prevent the paper from being properly typeset

You will receive further instructions from the production team, including instructions on how to review your proof when it is ready. Please keep in mind that we are working through a large volume of accepted articles, so please give us a few days to review your paper and let you know the next and final steps.

Lastly, if your institution or institutions have a press office, please let them know about your upcoming paper now to help maximize its impact. If they'll be preparing press materials, please inform our press team within the next 48 hours. Your manuscript will remain under strict press embargo until 2 pm Eastern Time on the date of publication. For more information, please contact onepress@plos.org.

You will receive an invoice from PLOS for your publication fee after your manuscript has reached the completed accept phase. If you receive an email requesting payment before acceptance or for any other service, this may be a phishing scheme. Learn how to identify phishing emails and protect your accounts at https://explore.plos.org/phishing.

If we can help with anything else, please email us at customercare@plos.org.

Thank you for submitting your work to PLOS One and supporting open access.

Kind regards,

PLOS One Editorial Office Staff

on behalf of

Dr. Carlos P. Odriozola

Academic Editor

PLOS One
